# Supplementary material for: Examining geospatial and temporal distribution of invasive non-typhoidal Salmonella disease occurrence in sub-Saharan Africa: a systematic review and modelling study
Source: BMJ Open. 2024 Mar 14;14(3):e080501. doi: 10.1136/bmjopen-2023-080501 (PMC10941155; doi:10.1136/bmjopen-2023-080501)

Supplementary Material

S1. Search terms

1. PubMed

(((nontyph*[Tiab] OR non-typh*[Tiab] OR group[Tiab] OR typhimurium[Tiab] OR enteritidis[Tiab] OR Heidelberg[Tiab] OR Dublin[Tiab] OR Choleraesuis[Tiab] OR Newport[Tiab] OR Virchow[Tiab] OR Concord[Tiab] OR Brancaster[Tiab] OR freetown[Tiab] OR Infantis[Tiab] OR Isangi[Tiab]) AND (salmonella[Tiab] OR salmonellosis[Tiab])) OR iNTS[Tiab])

AND

("Bacteremia"[Mesh] OR bacteremia [Tiab] OR bacteraemia [Tiab] OR "Sepsis"[Mesh] OR sepsis [Tiab] OR septic*[Tiab] OR "Invasive"[Tiab] OR bloodstream*[Tiab] OR “blood culture”[Tiab] OR “blood-culture”[Tiab] OR hemoculture OR incidence OR epidemiolog* OR burden OR case OR infect* OR prevalen*[Tiab])

AND

hasabstract

AND

“Humans”[Mesh]

AND

("2000/01/01"[Dp] : "2020/12/31"[Dp])

AND

("Africa South of the Sahara"[Mesh] OR "sub-saharan Africa"[Tiab] OR "subsaharan Africa"[Tiab] OR "Sub Saharan Africa"[Tiab] OR "Africa, Central"[Mesh] OR "Central Africa"[Tiab] OR "Cameroon"[Mesh] OR "Cameroon"[Tiab] OR "Central African Republic"[Mesh] OR "Central African Republic"[Tiab] OR "Chad"[Mesh] OR "Chad"[Tiab] OR "Congo"[Mesh] OR "Congo"[Tiab] OR "Democratic Republic of the Congo"[Mesh] OR "Democratic Republic of the Congo"[Tiab] OR "Equatorial Guinea"[Mesh] OR "Equatorial Guinea"[Tiab] OR "Gabon"[Mesh] OR "Gabon"[Tiab] OR "Sao Tome and Principe"[Mesh] OR "Sao Tome and Principe"[Tiab] OR "Africa, Eastern"[Mesh] OR "Eastern Africa"[Tiab] OR "East Africa"[Tiab] OR "Burundi"[Mesh] OR "Burundi"[Tiab] OR "Djibouti"[Mesh] OR "Djibouti"[Tiab] OR "Eritrea"[Mesh] OR "Eritrea"[Tiab] OR "Ethiopia"[Mesh] OR "Ethiopia" [Tiab] OR "Kenya"[Mesh] OR "Kenya" [Tiab] OR "Rwanda"[Mesh] OR "Rwanda" [Tiab] OR "Somalia"[Mesh] OR "Somalia"[Tiab] OR "South Sudan"[Mesh] OR "South Sudan"[Tiab] OR "Sudan"[Mesh] OR "Sudan"[Tiab] OR "Tanzania"[Mesh] OR "Tanzania"[Tiab] OR "Uganda"[Mesh] OR "Uganda"[Tiab] OR "Africa,

Southern"[Mesh] OR "Southern Africa"[Tiab] OR "South Africa"[Tiab] OR "Angola"[Mesh] OR "Angola"[Tiab] OR "Botswana"[Mesh] OR "Botswana"[Tiab] OR "Lesotho"[Mesh] OR "Lesotho"[Tiab] OR "Malawi"[Mesh] OR "Malawi"[Tiab] OR "Mozambique"[Mesh] OR "Mozambique"[Tiab] OR "Namibia"[Mesh] OR "Namibia"[Tiab] OR "South Africa"[Mesh] OR "South Africa"[Tiab] OR "Swaziland"[Mesh] OR "Swaziland"[Tiab] OR "Zambia"[Mesh] OR "Zambia"[Tiab] OR "Zimbabwe"[Mesh] OR "Zimbabwe"[Tiab] OR "Africa, Western"[Mesh] OR "Western Africa"[Tiab] OR "West Africa"[Tiab] OR "Benin"[Mesh] OR "Benin"[Tiab] OR "Burkina Faso"[Mesh] OR "Burkina Faso"[Tiab] OR "Cabo Verde"[Mesh] OR "Cabo Verde"[Tiab] OR "Cote d'Ivoire"[Mesh] OR "Cote d'Ivoire"[Tiab] OR "Gambia"[Mesh] OR "Gambia"[Tiab] OR "Ghana"[Mesh] OR "Ghana"[Tiab] OR "Guinea"[Mesh] OR "Guinea"[Tiab] OR "Guinea-Bissau"[Mesh] OR "Guinea-Bissau"[Tiab] OR "Liberia"[Mesh] OR "Liberia"[Tiab] OR "Mali"[Mesh] OR "Mali"[Tiab] OR "Mauritania"[Mesh] OR "Mauritania"[Tiab] OR "Niger"[Mesh] OR "Niger"[Tiab] OR "Nigeria"[Mesh] OR "Nigeria"[Tiab] OR "Senegal"[Mesh] OR "Senegal"[Tiab] OR "Sierra Leone"[Mesh] OR "Sierra Leone"[Tiab] OR "Togo"[Mesh] OR "Togo"[Tiab] OR "Comoros"[Mesh] OR "Comoros"[Tiab] OR "Mayotte"[Tiab] OR "Madagascar"[Mesh] OR "Madagascar"[Tiab] OR "Sahel"[Tiab])

2. Embase

(((‘nontyph*’:ti,ab OR ‘non-typh*’:ti,ab OR ‘group’:ti,ab OR ‘typhimurium’:ti,ab OR ‘enteritidis’:ti,ab OR ‘Heidelberg’:ti,ab OR ‘Dublin’:ti,ab OR ‘Choleraesuis’:ti,ab OR ‘Newport’:ti,ab OR ’Virchow’:ti,ab OR ‘Concord’:ti,ab OR ‘Brancaster’:ti,ab OR ‘freetown’:ti,ab OR ‘Infantis’:ti,ab OR ‘Isangi’:ti,ab) AND (‘salmonella’:ti,ab OR ‘salmonellosis’:ti,ab)) OR ‘iNTS’:ti,ab)

AND

(‘bacteremia’:ti,ab OR ‘sepsis’:ti,ab OR ‘septic*’:ti,ab OR ‘Invasive’:ti,ab OR ‘bloodstream*’:ti,ab OR ‘blood culture’:ti,ab OR ‘blood-culture’:ti,ab OR ‘hemoculture’:ti,ab OR ‘incidence’:ti,ab OR ‘epidemiolog*’:ti,ab OR ‘burden’:ti,ab OR ‘case’:ti,ab OR ‘infect*’:ti,ab OR ‘prevalen*’:ti,ab)

AND

[2000-2020]/py

AND

'Africa south of the Sahara'/exp OR ‘Sub-Saharan Africa’:ti,ab OR ‘Subsaharan Africa’:ti,ab OR ‘Sub Saharan Africa’:ti,ab OR 'Central Africa'/exp OR 'Central Africa':ti,ab OR 'Cameroon'/exp OR 'Cameroon':ti,ab OR 'Central African Republic'/exp OR 'Central African Republic':ti,ab OR 'Chad'/exp OR 'Chad':ti,ab OR 'Congo'/exp OR 'Congo':ti,ab OR 'Democratic Republic Congo'/exp OR 'Democratic Republic Congo':ti,ab OR 'Equatorial Guinea'/exp OR 'Equatorial Guinea':ti,ab OR 'Gabon'/exp OR 'Gabon':ti,ab OR 'Sao Tome and Principe'/exp OR 'Sao Tome and Principe':ti,ab OR ‘Eastern Africa’:ti,ab OR ‘East Africa’:ti,ab OR 'Burundi'/exp OR 'Burundi':ti,ab OR 'Djibouti'/exp OR 'Djibouti':ti,ab OR 'Eritrea'/exp OR 'Eritrea':ti,ab OR 'Ethiopia'/exp OR 'Ethiopia':ti,ab OR 'Kenya'/exp OR 'Kenya':ti,ab OR 'Rwanda'/exp OR 'Rwanda':ti,ab OR 'Somalia'/exp OR 'Somalia':ti,ab OR 'South Sudan'/exp OR 'South

Sudan':ti,ab OR 'Sudan'/exp OR 'Sudan':ti,ab OR 'Tanzania'/exp OR 'Tanzania':ti,ab OR 'Uganda'/exp OR 'Uganda':ti,ab OR 'Southern Africa':ti,ab OR 'South Africa':ti,ab OR 'Angola'/exp OR 'Angola':ti,ab OR 'Botswana'/exp OR 'Botswana':ti,ab OR 'Lesotho'/exp OR 'Lesotho':ti,ab OR 'Malawi'/exp OR 'Malawi':ti,ab OR 'Mozambique'/exp OR 'Mozambique':ti,ab OR 'Namibia'/exp OR 'Namibia':ti,ab OR 'South Africa'/exp OR 'South Africa':ti,ab OR 'Swaziland'/exp OR 'Swaziland':ti,ab OR 'Zambia'/exp OR 'Zambia':ti,ab OR 'Zimbabwe'/exp OR 'Zimbabwe':ti,ab OR 'Western Africa':ti,ab OR 'West Africa':ti,ab OR 'Benin'/exp OR 'Benin':ti,ab OR 'Burkina Faso'/exp OR 'Burkina Faso':ti,ab OR 'Cape Verde'/exp OR 'Cape Verde':ti,ab OR 'Cote d`Ivoire'/exp OR 'Cote d`Ivoire':ti,ab OR 'Gambia'/exp OR 'Gambia':ti,ab OR 'Ghana'/exp OR 'Ghana':ti,ab OR 'Guinea'/exp OR 'Guinea':ti,ab OR 'Guinea-Bissau'/exp OR 'Guinea-Bissau':ti,ab OR 'Liberia'/exp OR 'Liberia':ti,ab OR 'Mali'/exp OR 'Mali':ti,ab OR 'Mauritania'/exp OR 'Mauritania':ti,ab OR 'Niger'/exp OR 'Niger':ti,ab OR 'Nigeria'/exp OR 'Nigeria':ti,ab OR 'Senegal'/exp OR 'Senegal':ti,ab OR 'Sierra Leone'/exp OR 'Sierra Leone':ti,ab OR 'Togo'/exp OR 'Togo':ti,ab OR 'Comoros'/exp OR 'Comoros':ti,ab OR 'Mayotte'/exp OR 'Mayotte':ti,ab OR 'Madagascar'/exp OR 'Madagascar':ti,ab OR 'Sahel'/exp OR 'Sahel':ti,ab

3. Web of Science

(AB=(((nontyph* OR non-typh* OR group OR typhimurium OR enteritidis OR Heidelberg OR Dublin OR Choleraesuis OR Newport OR Virchow OR Concord OR Brancaster OR freetown OR Infantis OR Isangi) AND (salmonella OR salmonellosis)) OR iNTS)

AND

AB=(bacteremia OR sepsis OR septic* OR Invasive OR bloodstream* OR blood culture OR blood-culture OR hemoculture OR incidence OR epidemiolog* OR burden OR case OR infect* OR prevalen*)

AND

AB=("Sub-Saharan Africa" OR "Subsaharan Africa" OR "Sub Saharan Africa" OR "Central Africa" OR Cameroon OR "Central African Republic" OR Chad OR Congo OR "Democratic Republic Congo" OR "Equatorial Guinea" OR Gabon OR "Sao Tome and Principe" OR "Eastern Africa" OR "East Africa" OR Burundi OR Djibouti OR Eritrea OR Ethiopia OR Kenya OR Rwanda OR Somalia OR "South Sudan" OR Sudan OR Tanzania OR Uganda OR "Southern Africa" OR "South Africa" OR Angola OR Botswana OR Lesotho OR Malawi OR Mozambique OR Namibia OR Swaziland OR Zambia OR Zimbabwe OR "Western Africa" OR "West Africa" OR Benin OR Burkina Faso OR "Cape Verde" OR "Cote d`Ivoire" OR Gambia OR Ghana OR Guinea OR "Guinea-Bissau" OR Liberia OR Mali OR Mauritania OR Niger OR Nigeria OR Senegal OR "Sierra Leone" OR Togo OR Comoros OR Mayotte OR Madagascar OR Sahel))

OR

(TI=(((nontyph* OR non-typh* OR group OR typhimurium OR enteritidis OR Heidelberg OR Dublin OR Choleraesuis OR Newport OR Virchow OR Concord OR Brancaster OR freetown OR Infantis OR Isangi) AND (salmonella OR salmonellosis)) OR iNTS)

AND

TI=(bacteremia OR sepsis OR septic* OR Invasive OR bloodstream* OR blood culture OR blood-culture OR hemoculture OR incidence OR epidemiolog* OR burden OR case OR infect* OR prevalen*)

AND
TI=("Sub-Saharan Africa" OR "Subsaharan Africa" OR "Sub Saharan Africa" OR "Central Africa" OR Cameroon OR "Central African Republic" OR Chad OR Congo OR "Democratic Republic Congo" OR "Equatorial Guinea" OR Gabon OR "Sao Tome and Principe" OR "Eastern Africa" OR "East Africa" OR Burundi OR Djibouti OR Eritrea OR Ethiopia OR Kenya OR Rwanda OR Somalia OR "South Sudan" OR Sudan OR Tanzania OR Uganda OR "Southern Africa" OR "South Africa" OR Angola OR Botswana OR Lesotho OR Malawi OR Mozambique OR Namibia OR Swaziland OR Zambia OR Zimbabwe OR "Western Africa" OR "West Africa" OR Benin OR Burkina Faso OR "Cape Verde" OR "Cote d`Ivoire" OR Gambia OR Ghana OR Guinea OR "Guinea-Bissau" OR Liberia OR Mali OR Mauritania OR Niger OR Nigeria OR Senegal OR "Sierra Leone" OR Togo OR Comoros OR Mayotte OR Madagascar OR Sahel))

S2. Explanatory covariates

Figure S1. Mean estimates for the prevalence of HIV infection among 15-45 year olds in sub-Saharan Africa in 2017 [1].

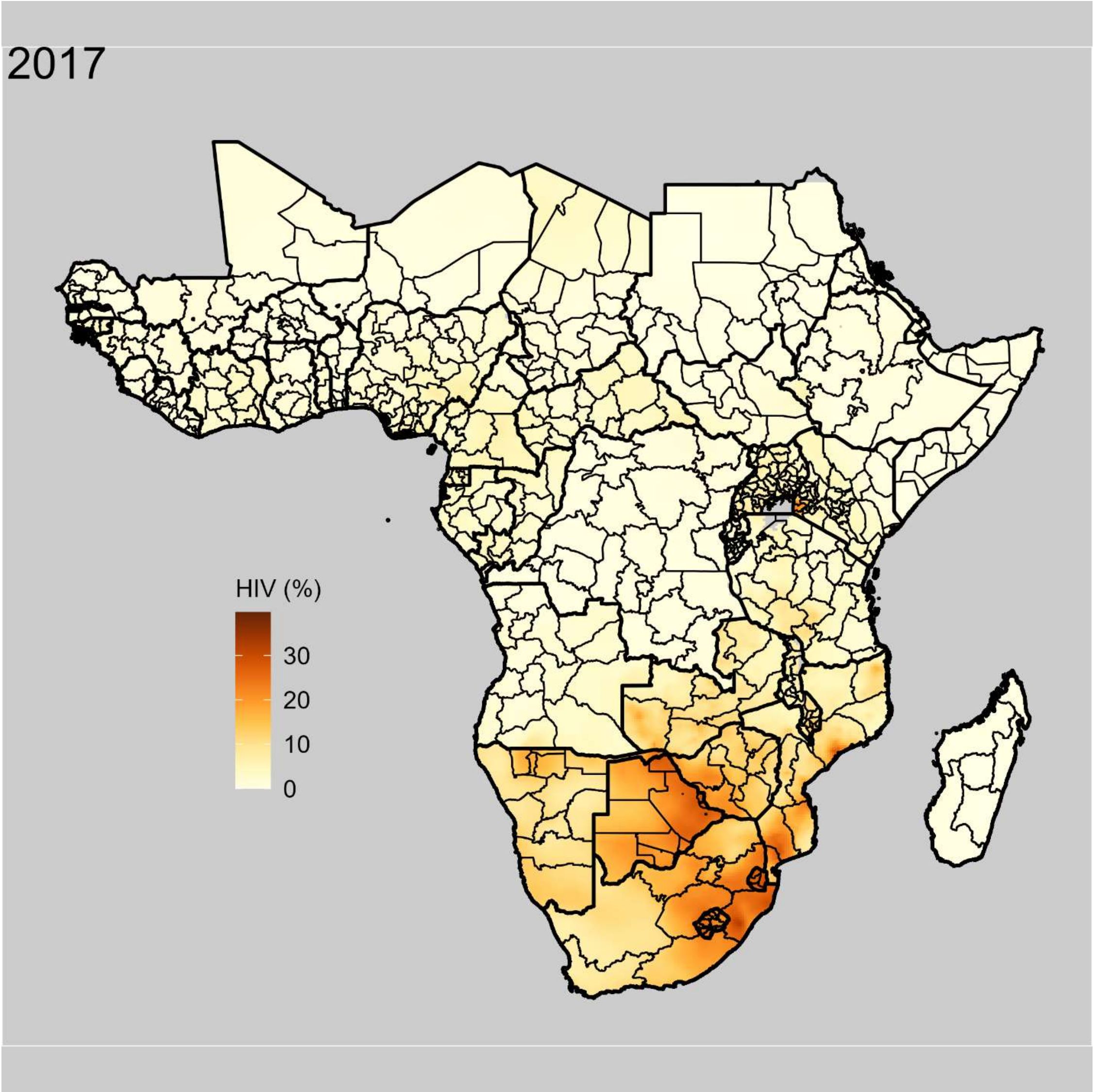

Figure S2. Incidence rate of *Plasmodium Falciparum* [2], 2017. The values indicate the number of newly diagnosed Plasmodium falciparum cases per person in 2017. Areas for which data are not available are not colored.

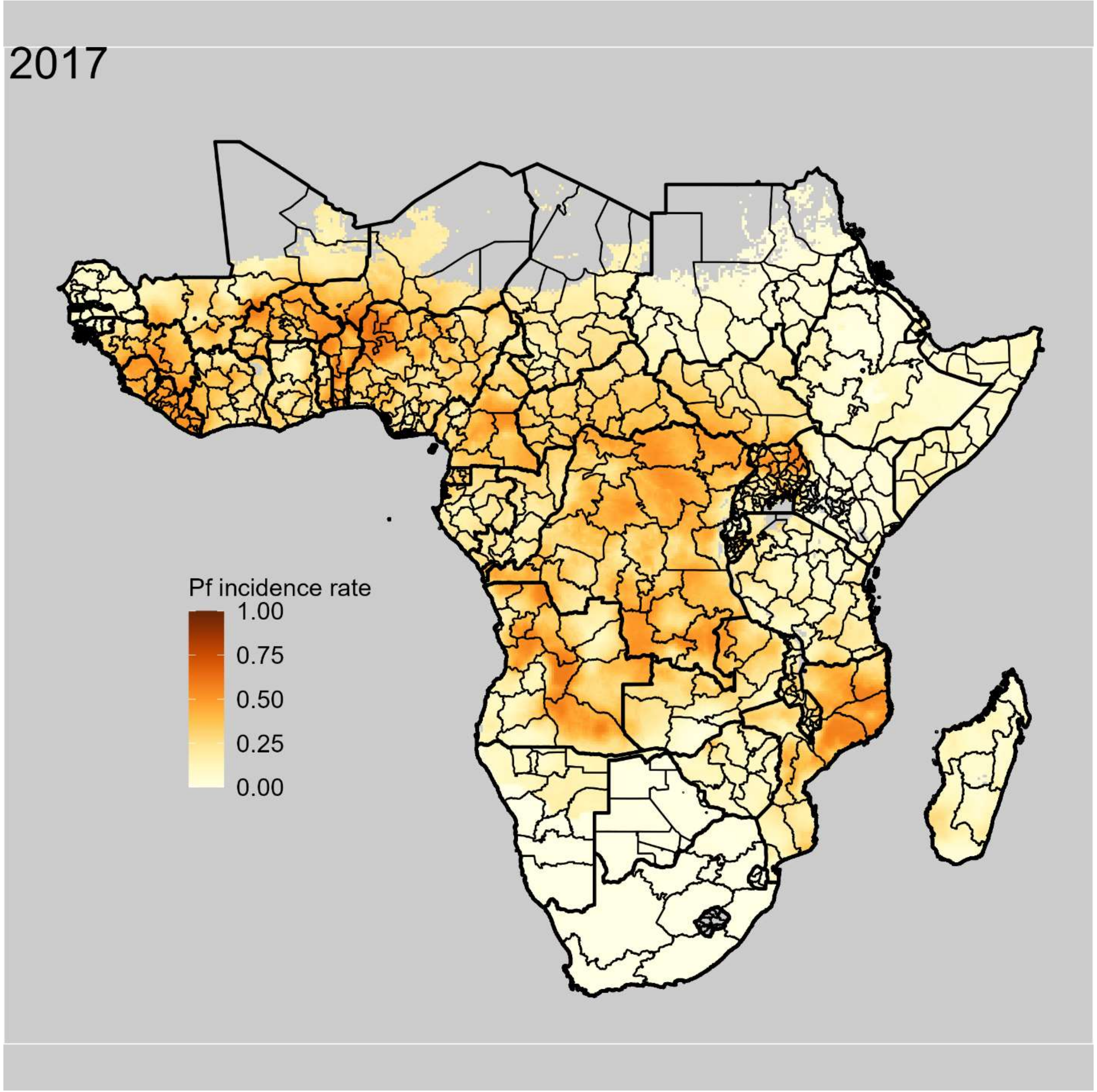

Figure S3. Prevalence of underweight [3].

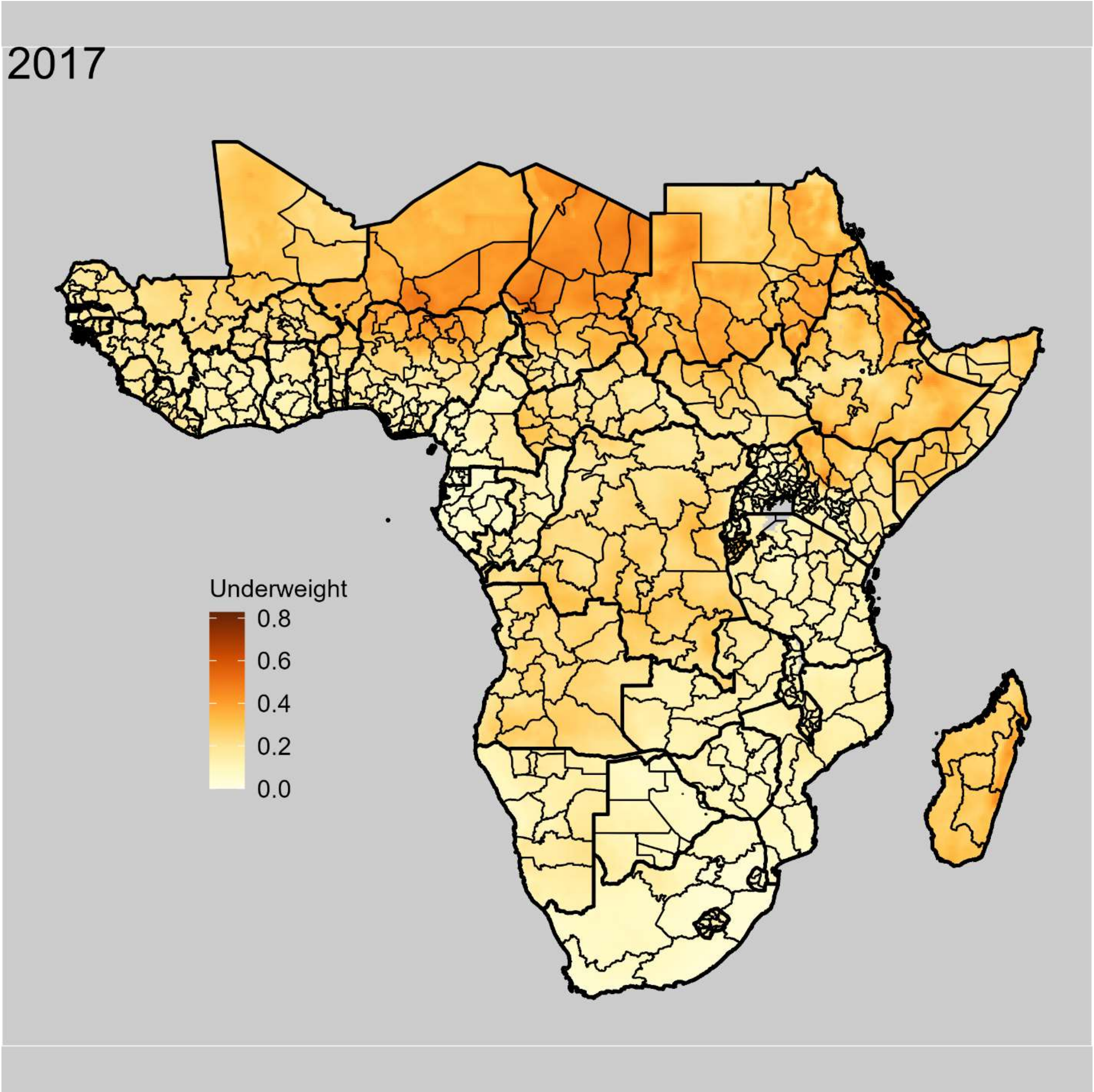

Figure S4. Percentage access to improved drinking water source. The values are based on the estimates on the mean access to any improved water sources on 5 km by 5 km grids [4]. Areas for which data are not available are not colored.

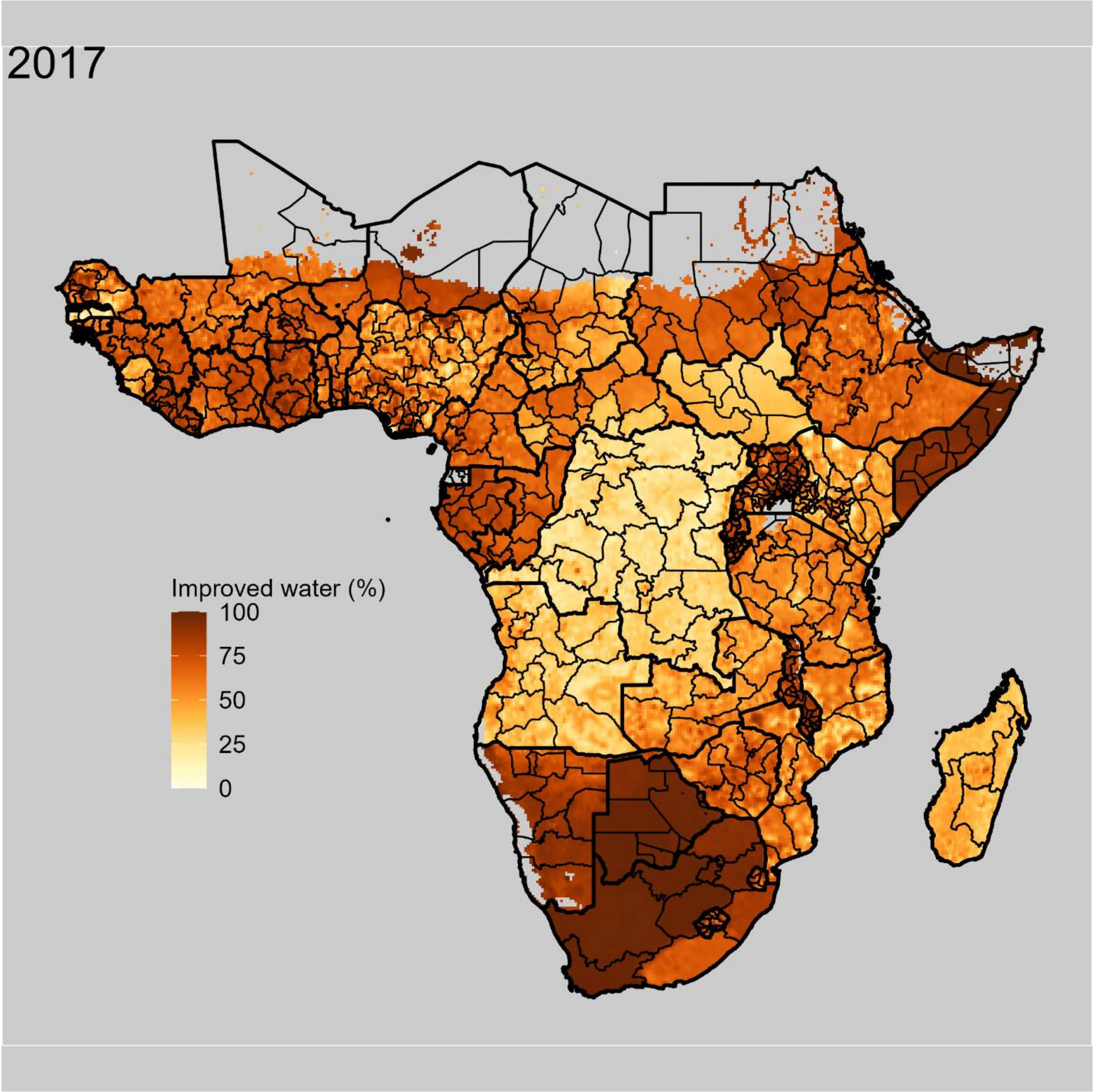

Figure S5. Percentage access to improved sanitation facilities. The values are based on the recent estimates on the mean access to any improved sanitation facilities on 5 km by 5 km grids [4]. Areas for which data are not available are not colored.

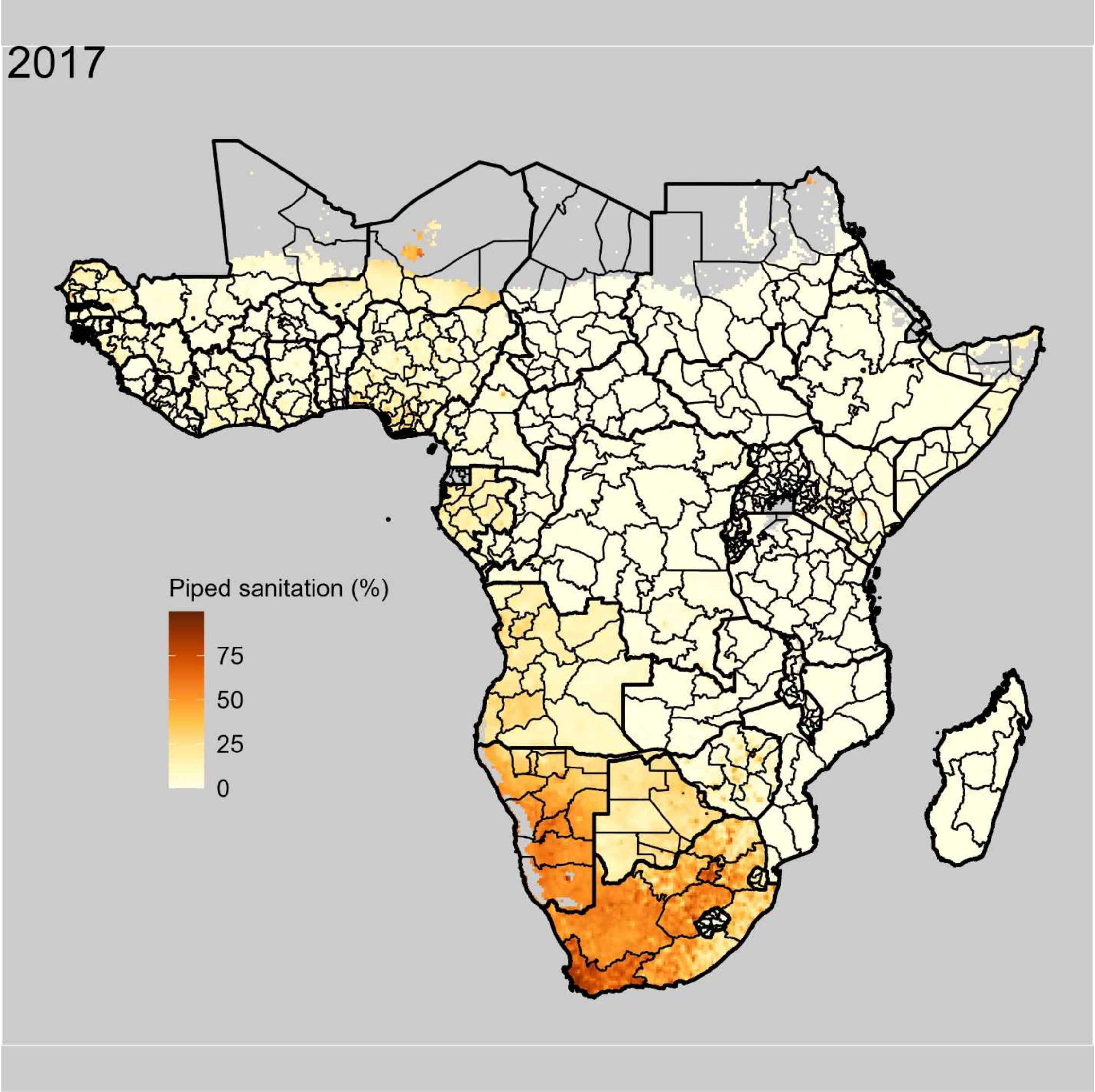

Figure S6. Travel time to the nearest healthcare facility. The values are based on a study that produce maps of travel time without access to motorized transport at 1 km by 1 km grids [5].

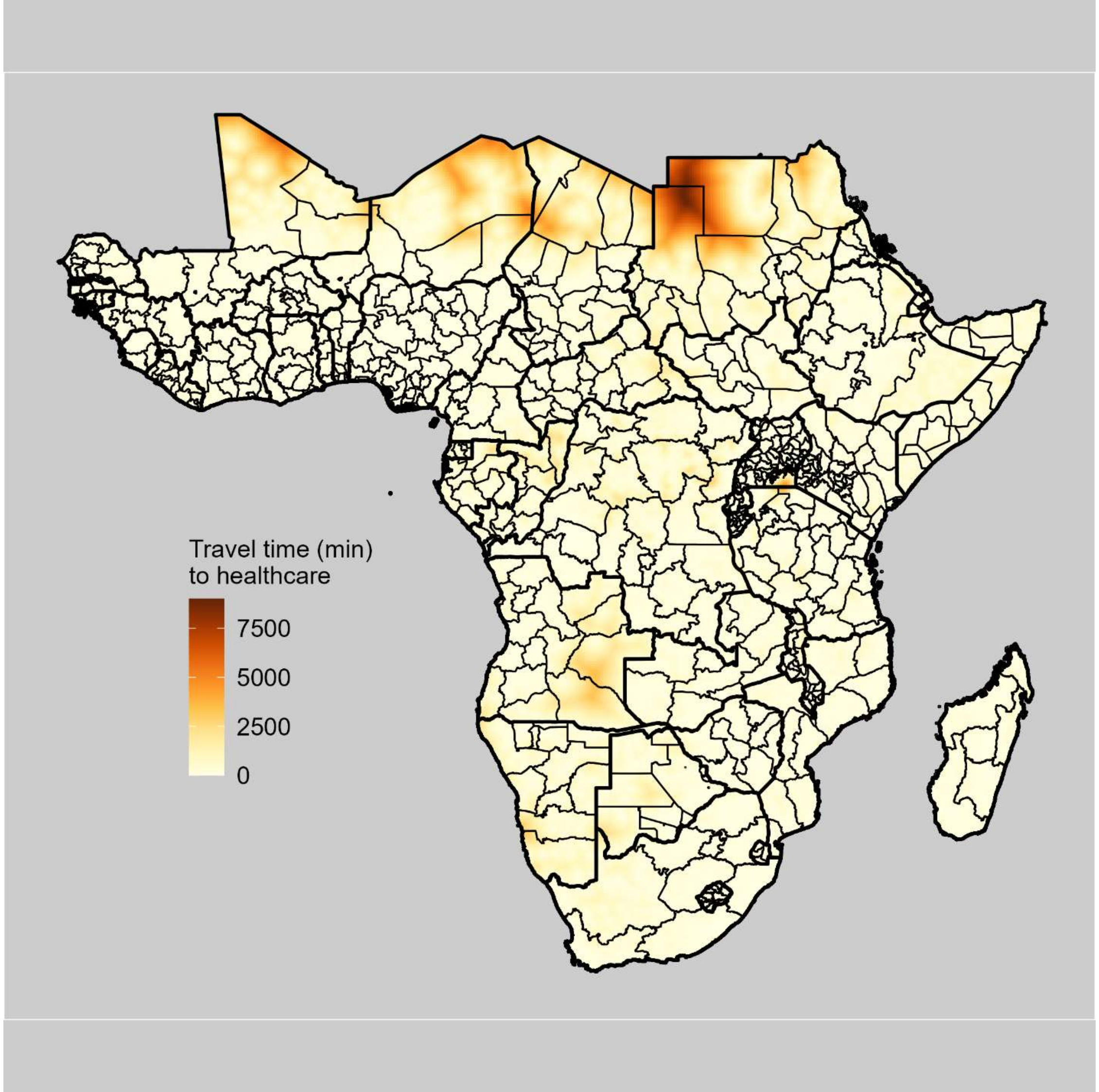

Table S1. Correlation among covariates. Pearson's *r* was computed using a data set of distance decay constant *k*=0.01. Highlighted cells indicate correlation coefficients among variables included in the final model.

	Pf incidence rate	Pf parasite rate	Improved water*	Open defecation	Piped sanitation*	Piped water	HIV*	Stunting	Surface water	Underweight*	Wasting
Pf incidence* rate	1	0.95	-0.35	-0.01	-0.46	-0.44	-0.15	0.3	0.04	0.08	-0.17
Pf parasite rate	0.95	1	-0.31	-0.05	-0.38	-0.36	-0.16	0.25	0.03	0.07	-0.13
Improved water*	-0.35	-0.31	1	-0.14	0.51	0.68	0.37	-0.45	-0.56	-0.39	-0.09
Open defecation	-0.01	-0.05	-0.14	1	-0.21	-0.09	-0.09	0.13	0.16	0.34	0.32
Piped sanitation*	-0.46	-0.38	0.51	-0.21	1	0.68	0.24	-0.29	-0.14	-0.28	-0.1
Piped water	-0.44	-0.36	0.68	-0.09	0.68	1	0.39	-0.35	-0.28	-0.35	-0.13
HIV*	-0.15	-0.16	0.37	-0.09	0.24	0.39	1	-0.17	-0.14	-0.57	-0.47
Stunting	0.3	0.25	-0.45	0.13	-0.29	-0.35	-0.17	1	0.34	0.58	0.08
Surface water	0.04	0.03	-0.56	0.16	-0.14	-0.28	-0.14	0.34	1	0.14	-0.11
Underweight*	0.08	0.07	-0.39	0.34	-0.28	-0.35	-0.57	0.58	0.14	1	0.76
Wasting	-0.17	-0.13	-0.09	0.32	-0.1	-0.13	-0.47	0.08	-0.11	0.76	1

* Covariates included in the final model

Figure S7. Receiver operating curve (ROC) from boosted regression tree model fits. The solid line represents the mean and dashed lines represent lower and upper bounds of the 95% confidence intervals. Area Under the ROC Curve (AUC) is 0.913 [95% CI: 0.911 to 0.915].

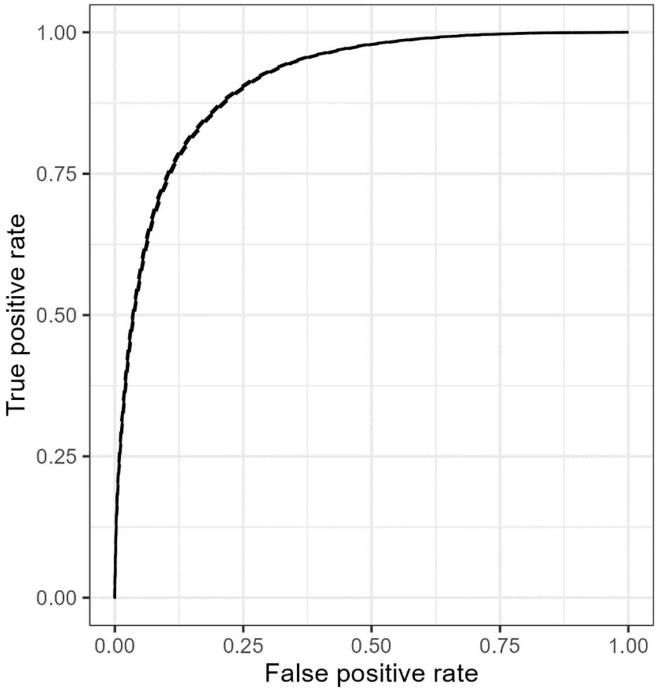

Table S2. Probability of iNTS occurrence for 2017 aggregated at the country level. Mean and the 95% confidence interval of the predicted probability from 400 simulation runs.

Country	Probability of occurrence
Angola	0.14 (95% CI: 0.07 to 0.25)
Burundi	0.32 (95% CI: 0.17 to 0.52)
Benin	0.25 (95% CI: 0.11 to 0.48)
Burkina Faso	0.29 (95% CI: 0.14 to 0.53)
Botswana	0.07 (95% CI: 0.03 to 0.18)
Central African Republic	0.47 (95% CI: 0.30 to 0.64)
Cote d'Ivoire	0.40 (95% CI: 0.24 to 0.61)
Cameroon	0.33 (95% CI: 0.19 to 0.50)
Congo, Democratic Republic of the	0.46 (95% CI: 0.26 to 0.68)
Congo, Republic of the	0.60 (95% CI: 0.40 to 0.78)

Djibouti	0.07 (95% CI: 0.03 to 0.13)
Eritrea	0.01 (95% CI: 0.01 to 0.03)
Ethiopia	0.06 (95% CI: 0.03 to 0.11)
Gabon	0.13 (95% CI: 0.03 to 0.41)
Ghana	0.66 (95% CI: 0.37 to 0.87)
Guinea	0.66 (95% CI: 0.41 to 0.86)
Gambia	0.42 (95% CI: 0.21 to 0.64)
Guinea-Bissau	0.59 (95% CI: 0.39 to 0.79)
Equatorial Guinea	0.02 (95% CI: 0.00 to 0.14)
Kenya	0.34 (95% CI: 0.19 to 0.51)
Liberia	0.48 (95% CI: 0.22 to 0.77)
Lesotho	0.25 (95% CI: 0.10 to 0.48)
Madagascar	0.03 (95% CI: 0.02 to 0.07)
Mali	0.38 (95% CI: 0.24 to 0.55)
Mozambique	0.58 (95% CI: 0.34 to 0.78)
Malawi	0.65 (95% CI: 0.49 to 0.81)
Namibia	0.10 (95% CI: 0.04 to 0.20)
Niger	0.02 (95% CI: 0.01 to 0.04)
Nigeria	0.21 (95% CI: 0.11 to 0.36)
Rwanda	0.38 (95% CI: 0.18 to 0.68)
Sudan	0.01 (95% CI: 0.00 to 0.01)
Senegal	0.30 (95% CI: 0.12 to 0.52)
Sierra Leone	0.23 (95% CI: 0.10 to 0.45)
Somalia	0.03 (95% CI: 0.01 to 0.07)
South Sudan	0.13 (95% CI: 0.07 to 0.25)
Swaziland	0.24 (95% CI: 0.08 to 0.74)
Chad	0.11 (95% CI: 0.06 to 0.19)
Togo	0.65 (95% CI: 0.42 to 0.82)
Tanzania, United Republic of	0.15 (95% CI: 0.06 to 0.33)
Uganda	0.23 (95% CI: 0.09 to 0.47)
South Africa	0.07 (95% CI: 0.03 to 0.18)
Zambia	0.49 (95% CI: 0.29 to 0.69)
Zimbabwe	0.40 (95% CI: 0.20 to 0.63)

Figure S8. Probability of iNTS occurrence from 2000 to 2020 every two years. Estimates represent the mean from 400 simulation runs. Areas for which data are not available are not colored. For animation, see https://github.com/kimfinale/iNTS_Mapping/tree/master/images.

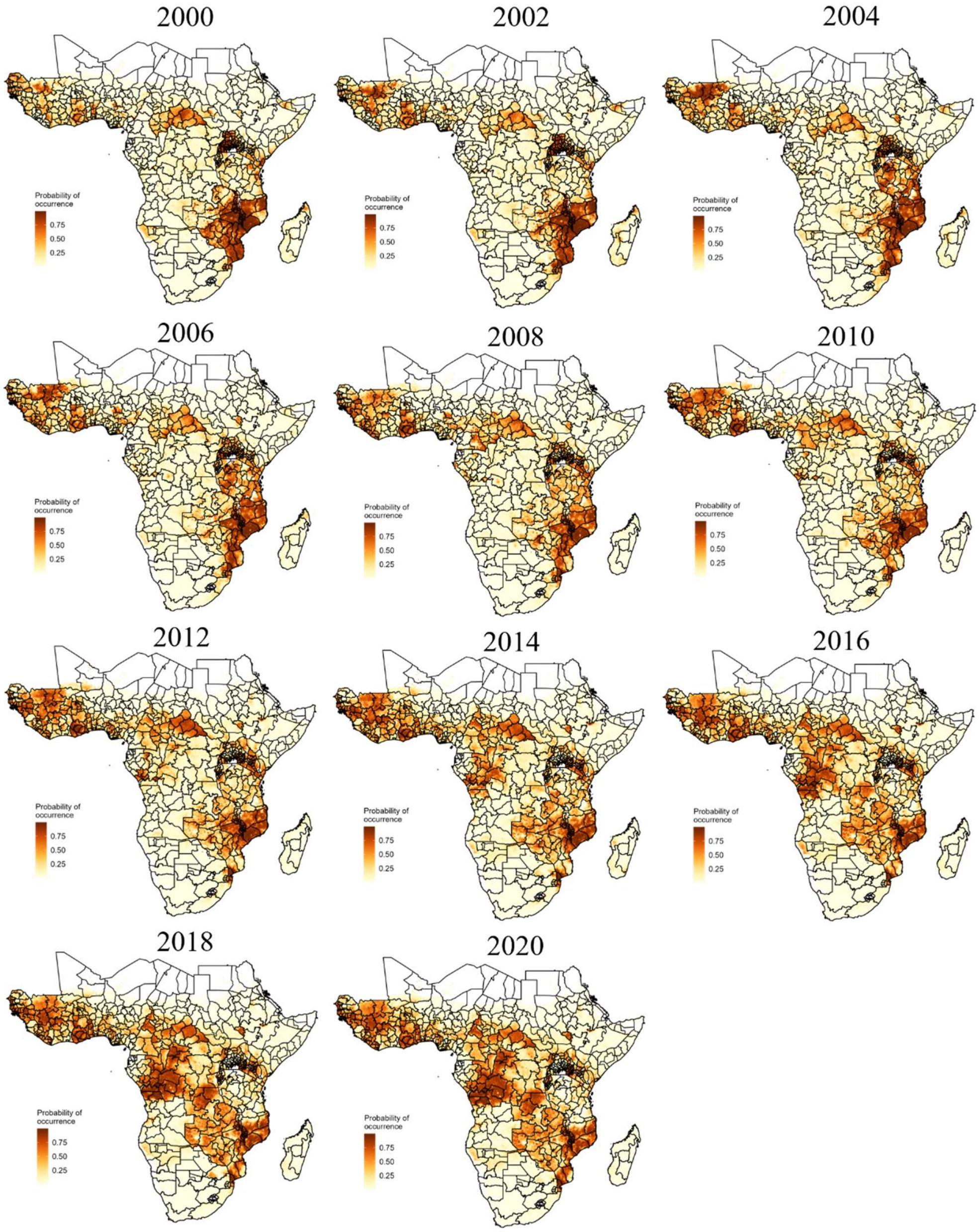

Table S3. Probability of iNTS occurrence aggregated at the regional level over 2000 - 2020. Values represent means from 400 simulation runs.

	Eastern Africa	Middle Africa	Southern Africa	Western Africa
2000	0.27	0.11	0.05	0.19
2001	0.28	0.11	0.06	0.19
2002	0.26	0.11	0.05	0.22
2003	0.27	0.11	0.05	0.24
2004	0.29	0.11	0.06	0.25
2005	0.28	0.12	0.05	0.25
2006	0.27	0.12	0.05	0.27
2007	0.27	0.14	0.05	0.27
2008	0.25	0.14	0.05	0.26
2009	0.25	0.16	0.04	0.28
2010	0.26	0.15	0.04	0.29
2011	0.25	0.16	0.04	0.30
2012	0.26	0.18	0.04	0.32
2013	0.26	0.20	0.04	0.32
2014	0.26	0.22	0.06	0.32
2015	0.25	0.25	0.04	0.33
2016	0.25	0.29	0.05	0.33
2017	0.24	0.34	0.08	0.33
2018	0.24	0.35	0.06	0.33
2019	0.24	0.35	0.05	0.33
2020	0.24	0.35	0.06	0.33

Table S4. Probability of iNTS occurrence aggregated at the country level over 2000 - 2020. Values represent means from 400 simulation runs.

Country	2000	2001	2002	2003	2004	2005	2006	2007	2008	2009	2010	2011	2012	2013	2014	2015	2016	2017	2018	2019	2020
Angola	0.05	0.05	0.05	0.05	0.05	0.05	0.05	0.05	0.05	0.05	0.05	0.05	0.06	0.08	0.09	0.11	0.13	0.14	0.14	0.14	0.14
Burundi	0.30	0.29	0.27	0.24	0.17	0.17	0.19	0.23	0.29	0.33	0.32	0.31	0.29	0.26	0.27	0.27	0.30	0.32	0.35	0.36	0.37
Benin	0.28	0.29	0.30	0.27	0.20	0.19	0.22	0.19	0.19	0.19	0.16	0.19	0.22	0.24	0.24	0.26	0.26	0.25	0.24	0.25	0.25
Burkina Faso	0.08	0.06	0.05	0.05	0.06	0.06	0.06	0.08	0.12	0.15	0.21	0.26	0.31	0.30	0.28	0.24	0.22	0.29	0.29	0.29	0.30
Botswana	0.02	0.02	0.01	0.01	0.01	0.01	0.01	0.01	0.02	0.03	0.04	0.02	0.02	0.02	0.04	0.02	0.04	0.07	0.04	0.03	0.04
CAR	0.45	0.42	0.41	0.42	0.42	0.42	0.44	0.45	0.46	0.50	0.48	0.46	0.55	0.53	0.52	0.50	0.51	0.47	0.46	0.46	0.46
Cote d'Ivoire	0.26	0.25	0.24	0.23	0.24	0.28	0.29	0.26	0.22	0.21	0.23	0.26	0.34	0.44	0.47	0.46	0.45	0.40	0.40	0.39	0.39
Cameroon	0.05	0.05	0.06	0.06	0.08	0.09	0.09	0.16	0.25	0.34	0.29	0.29	0.26	0.27	0.28	0.30	0.32	0.33	0.33	0.34	0.33
DR Congo,	0.09	0.09	0.09	0.10	0.10	0.10	0.10	0.10	0.10	0.11	0.11	0.13	0.15	0.17	0.20	0.25	0.34	0.46	0.48	0.48	0.48
Congo	0.05	0.05	0.05	0.07	0.07	0.08	0.09	0.10	0.12	0.15	0.19	0.23	0.28	0.38	0.52	0.63	0.65	0.60	0.59	0.59	0.59
Djibouti	0.02	0.02	0.03	0.04	0.03	0.05	0.05	0.04	0.03	0.04	0.03	0.03	0.03	0.03	0.04	0.05	0.06	0.07	0.08	0.11	0.12
Eritrea	0.04	0.05	0.04	0.04	0.02	0.02	0.02	0.02	0.01	0.01	0.01	0.01	0.01	0.01	0.01	0.01	0.01	0.01	0.01	0.02	0.02
Ethiopia	0.02	0.02	0.03	0.04	0.04	0.03	0.03	0.03	0.03	0.04	0.05	0.05	0.07	0.08	0.06	0.06	0.06	0.06	0.05	0.05	0.06
Gabon	0.03	0.04	0.06	0.04	0.07	0.08	0.12	0.21	0.17	0.14	0.12	0.11	0.13	0.11	0.11	0.12	0.12	0.13	0.14	0.14	0.13
Ghana	0.43	0.43	0.57	0.55	0.51	0.52	0.58	0.64	0.66	0.66	0.64	0.61	0.61	0.60	0.60	0.62	0.67	0.66	0.64	0.64	0.66
Guinea	0.27	0.33	0.44	0.48	0.42	0.38	0.40	0.45	0.50	0.55	0.56	0.56	0.61	0.64	0.66	0.66	0.66	0.66	0.67	0.68	0.68
Gambia	0.35	0.43	0.51	0.54	0.50	0.48	0.50	0.48	0.48	0.43	0.43	0.46	0.51	0.54	0.53	0.54	0.54	0.42	0.43	0.41	0.43
Guinea-Bissau	0.45	0.41	0.38	0.40	0.46	0.48	0.50	0.52	0.52	0.52	0.52	0.52	0.54	0.54	0.56	0.57	0.59	0.59	0.59	0.57	0.55
Equatorial Guinea	0.20	0.20	0.22	0.17	0.11	0.05	0.03	0.03	0.03	0.04	0.05	0.04	0.03	0.02	0.01	0.01	0.01	0.02	0.02	0.02	0.02
Kenya	0.19	0.20	0.20	0.20	0.19	0.20	0.20	0.22	0.27	0.27	0.30	0.31	0.36	0.34	0.32	0.32	0.33	0.34	0.32	0.30	0.30
Liberia	0.07	0.08	0.10	0.14	0.22	0.34	0.45	0.53	0.57	0.53	0.49	0.49	0.50	0.50	0.49	0.46	0.43	0.48	0.54	0.57	0.59
Lesotho	0.03	0.03	0.04	0.03	0.03	0.04	0.04	0.05	0.06	0.08	0.10	0.12	0.12	0.14	0.18	0.19	0.22	0.25	0.25	0.25	0.25
Madagascar	0.07	0.13	0.11	0.11	0.08	0.09	0.07	0.04	0.03	0.02	0.02	0.02	0.03	0.04	0.05	0.04	0.03	0.03	0.03	0.03	0.03
Mali	0.18	0.24	0.32	0.42	0.46	0.41	0.45	0.42	0.34	0.44	0.47	0.47	0.46	0.46	0.43	0.41	0.41	0.38	0.36	0.36	0.39
Mozambique	0.77	0.80	0.81	0.80	0.79	0.77	0.75	0.73	0.70	0.68	0.67	0.68	0.65	0.63	0.60	0.58	0.59	0.58	0.59	0.59	0.60
Malawi	0.88	0.88	0.87	0.84	0.82	0.80	0.76	0.70	0.65	0.62	0.60	0.64	0.64	0.63	0.64	0.63	0.64	0.65	0.64	0.64	0.66

Namibia	0.08	0.08	0.06	0.05	0.05	0.04	0.05	0.04	0.03	0.02	0.02	0.02	0.02	0.03	0.08	0.07	0.10	0.10	0.10	0.11	0.09
Niger	0.02	0.02	0.02	0.02	0.02	0.02	0.02	0.02	0.02	0.02	0.02	0.02	0.02	0.01	0.01	0.02	0.02	0.02	0.02	0.02	0.02
Nigeria	0.11	0.11	0.12	0.11	0.13	0.14	0.14	0.15	0.14	0.14	0.14	0.15	0.17	0.16	0.18	0.20	0.21	0.21	0.21	0.21	0.21
Rwanda	0.31	0.34	0.37	0.40	0.43	0.43	0.39	0.36	0.30	0.25	0.22	0.25	0.28	0.33	0.45	0.49	0.41	0.38	0.38	0.41	0.59
Sudan	0.00	0.00	0.00	0.00	0.00	0.00	0.00	0.00	0.00	0.00	0.00	0.00	0.00	0.01	0.01	0.01	0.01	0.01	0.01	0.01	0.01
Senegal	0.44	0.26	0.27	0.35	0.31	0.31	0.36	0.35	0.34	0.29	0.35	0.32	0.34	0.31	0.26	0.31	0.27	0.30	0.35	0.29	0.36
Sierra Leone	0.24	0.21	0.17	0.30	0.42	0.47	0.45	0.46	0.39	0.30	0.30	0.23	0.24	0.27	0.29	0.28	0.25	0.23	0.23	0.23	0.22
Somalia	0.12	0.10	0.09	0.09	0.09	0.08	0.07	0.05	0.04	0.03	0.03	0.02	0.02	0.02	0.02	0.02	0.02	0.03	0.03	0.04	0.03
South Sudan	0.07	0.07	0.08	0.07	0.07	0.07	0.08	0.08	0.09	0.11	0.12	0.12	0.12	0.12	0.13	0.13	0.13	0.13	0.13	0.14	0.14
Swaziland	0.53	0.59	0.63	0.66	0.70	0.66	0.64	0.61	0.57	0.54	0.49	0.45	0.42	0.41	0.36	0.31	0.26	0.24	0.25	0.23	0.23
Chad	0.06	0.06	0.05	0.04	0.04	0.04	0.08	0.10	0.09	0.08	0.08	0.08	0.09	0.10	0.11	0.10	0.10	0.11	0.11	0.11	0.11
Togo	0.14	0.17	0.17	0.18	0.19	0.20	0.22	0.23	0.26	0.30	0.37	0.39	0.44	0.45	0.53	0.56	0.60	0.65	0.65	0.65	0.65
Tanzania	0.22	0.21	0.19	0.33	0.51	0.53	0.48	0.40	0.36	0.32	0.30	0.28	0.26	0.28	0.28	0.25	0.20	0.15	0.15	0.15	0.15
Uganda	0.65	0.65	0.63	0.60	0.59	0.56	0.51	0.47	0.46	0.45	0.43	0.41	0.36	0.31	0.27	0.24	0.23	0.23	0.22	0.21	0.23
South Africa	0.03	0.06	0.06	0.07	0.07	0.06	0.06	0.06	0.05	0.05	0.05	0.05	0.05	0.05	0.05	0.03	0.03	0.07	0.05	0.03	0.03
Zambia	0.32	0.33	0.29	0.27	0.25	0.25	0.25	0.26	0.28	0.30	0.32	0.35	0.39	0.43	0.45	0.47	0.49	0.49	0.49	0.48	0.48
Zimbabwe	0.61	0.52	0.44	0.37	0.34	0.29	0.30	0.48	0.36	0.39	0.46	0.33	0.37	0.37	0.38	0.38	0.37	0.40	0.41	0.40	0.37

Figure S9. Probability of iNTS occurrence for 2000, 2010, and 2017 for k=0.01, k=0.02, k=0.005. Average radius for the catchment area for the secondary and tertiary hospital is 100 km (baseline), 50 km, and 200 km, respectively for k=0.01, k=0.02, and k=0.005. Areas for which data are not available are not colored.

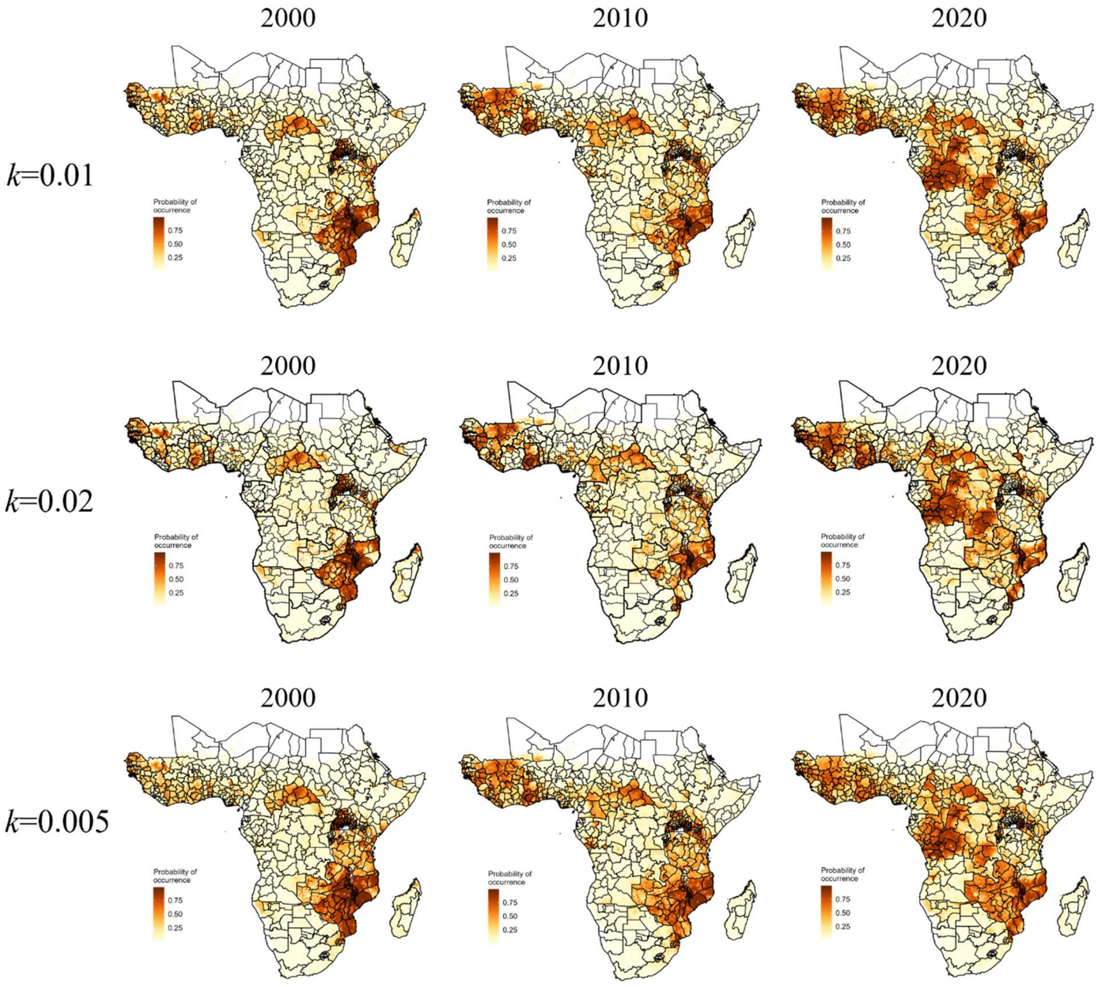

Supplement: Supplementary data [file bmjopen-2023-080501supp001.pdf]
