# Supplementary material for: Examining geospatial and temporal distribution of invasive non-typhoidal Salmonella disease occurrence in sub-Saharan Africa: a systematic review and modelling study
Source: BMJ Open. 2024 Mar 14;14(3):e080501. doi: 10.1136/bmjopen-2023-080501 (PMC10941155; doi:10.1136/bmjopen-2023-080501)
Supplement: Author's manuscript [file bmjopen-2023-080501.draft_revisions.pdf]

BMJ Open

BMJ Open is committed to open peer review. As part of this commitment we make the peer review history of every article we publish publicly available.

When an article is published we post the peer reviewers' comments and the authors' responses online. We also post the versions of the paper that were used during peer review. These are the versions that the peer review comments apply to.

The versions of the paper that follow are the versions that were submitted during the peer review process. They are not the versions of record or the final published versions. They should not be cited or distributed as the published version of this manuscript.

BMJ Open is an open access journal and the full, final, typeset and author-corrected version of record of the manuscript is available on our site with no access controls, subscription charges or pay-per-view fees (http://bmjopen.bmj.com).

If you have any questions on BMJ Open's open peer review process please email info.bmjopen@bmj.com

BMJ Open

Geospatial and temporal distribution of invasive non-typhoidal Salmonella disease occurrence in sub-Saharan Africa

Journal:	*BMJ Open*
Manuscript ID	bmjopen-2023-080501
Article Type:	Original research
Date Submitted by the Author:	04-Oct-2023
Complete List of Authors:	Kim, Jong-Hoon; International Vaccine Institute,<br>Tack, Bieke ; Institute of Tropical Medicine, Clinical Sciences<br>Fiorino, Fabio; University of Siena<br>Pettini, Elena; University of Siena<br>Marchello, Christian; New Zealand Ministry of Health<br>Jacobs, Jan; Institute of Tropical Medicine, Department of Clinical Sciences<br>Crump, John; University of Otago, Centre for International Health<br>Marks, Florian ; International Vaccine Institute, Epidemiology Unit; University of Cambridge, Department of Medicine
Keywords:	Epidemiology < TROPICAL MEDICINE, Epidemiology < INFECTIOUS DISEASES, Gastroduodenal disease < GASTROENTEROLOGY

SCHOLARONE™
Manuscripts

I, the Submitting Author has the right to grant and does grant on behalf of all authors of the Work (as defined in the below author licence), an exclusive licence and/or a non-exclusive licence for contributions from authors who are: i) UK Crown employees; ii) where BMJ has agreed a CC-BY licence shall apply, and/or iii) in accordance with the terms applicable for US Federal Government officers or employees acting as part of their official duties; on a worldwide, perpetual, irrevocable, royalty-free basis to BMJ Publishing Group Ltd ("BMJ") its licensees and where the relevant Journal is co-owned by BMJ to the co-owners of the Journal, to publish the Work in this journal and any other BMJ products and to exploit all rights, as set out in our licence.

The Submitting Author accepts and understands that any supply made under these terms is made by BMJ to the Submitting Author unless you are acting as an employee on behalf of your employer or a postgraduate student of an affiliated institution which is paying any applicable article publishing charge ("APC") for Open Access articles. Where the Submitting Author wishes to make the Work available on an Open Access basis (and intends to pay the relevant APC), the terms of reuse of such Open Access shall be governed by a Creative Commons licence – details of these licences and which Creative Commons licence will apply to this Work are set out in our licence referred to above.

Other than as permitted in any relevant BMJ Author's Self Archiving Policies, I confirm this Work has not been accepted for publication elsewhere, is not being considered for publication elsewhere and does not duplicate material already published. I confirm all authors consent to publication of this Work and authorise the granting of this licence.

For peer review only

Title

Geospatial and temporal distribution of invasive non-typhoidal *Salmonella* disease occurrence in sub-Saharan Africa

Authors

Jong-Hoon Kim*, Bieke Tack, Fabio Fiorino, Elena Pettini, Christian S. Marchello, Jan Jacobs, John A. Crump, Florian Marks, on behalf of the Vacc-iNTS consortium collaborators*

*Corresponding author

Email: jonghoon.kim@ivi.int

Postal address: SNU Research Park, 1 Gwanak-ro, Gwanak-gu, Seoul 08826, Republic of Korea

Word count: 3,954

For peer review only

Abstract

Background: Invasive non-typhoidal *Salmonella* (iNTS) disease is a significant health concern in sub-Saharan Africa. While our knowledge of a larger-scale variation is growing, understanding of the subnational variation in iNTS disease occurrence is lacking, yet crucial for targeted intervention.

Method: We conducted a systematic review of iNTS disease occurrences in sub-Saharan Africa from literature since 2000. We estimated the probability of occurrence of iNTS for sub-Saharan Africa on 20 km × 20 km grids by exploring the association with geospatial covariates such as malaria, HIV, childhood growth failure, access to improved water, and sanitation using a boosted regression tree.

Results: We identified 130 unique references reporting human iNTS infection in 21 countries published from 2000 through 2020. Estimated probability of iNTS occurrence grids showed significant spatial heterogeneity at all levels (20 km by 20 km grids, subnational, country, and subregional levels) and temporal heterogeneity by year. For 2020, the probability of occurrence was higher in Middle Africa (0.34, 95% CI: 0.25 to 0.46), followed by Western Africa (0.33, 95% CI: 0.23 to 0.44), Eastern Africa (0.24, 95% CI: 0.17 to 0.33), and Southern Africa (0.08, 95% CI: 0.03 to 0.11). Temporal heterogeneity indicated that the probability of occurrence increased between 2000 and 2020 in countries such as Republic of the Congo (0.05 to 0.59) and Democratic Republic of the Congo (0.10 to 0.48) whereas it decreased in countries such as Uganda (0.65 to 0.23) or Zimbabwe (0.61 to 0.37).

Conclusion: The iNTS disease occurrence varied greatly across sub-Saharan Africa, with certain regions being disproportionately affected. Identifying high-risk areas enables better surveillance and interventions, aiming to enhance public health outcomes in the region.

Keywords: Epidemiology; *Salmonella* Infections; *Salmonella* typhimurium; *Salmonella* enteritidis; Machine Learning; Sub-Saharan Africa

For peer review only

Introduction

Non-typhoidal *Salmonella* (NTS) are a leading cause of invasive bacterial disease in sub-Saharan Africa. Invasive non-typhoidal *Salmonella* (iNTS) disease was estimated to have caused 535,000 cases and 77,500 deaths in 2017 [1]. More than three-quarters of these cases and deaths occurred in sub-Saharan Africa, where the incidence rates were estimated at 44.8 per 100,000 person years observations and case fatality was estimated at 17.1% [2,3].

The geographic predominance of iNTS disease in sub-Saharan Africa can be partially explained by the prevalence of key host risk factors and the circulation of human-adapted NTS in the region [4,5]. While NTS strains circulating in other settings typically cause self-limiting foodborne diarrheal enterocolitis, genetic adaptations observed in the African strains resulted in increased virulence [6] and possibly in the establishment of a human reservoir [7]. Impaired immunity due to young age, recent or current *Plasmodium falciparum* malaria infection, anemia, malnutrition, and HIV infection renders the sub-Saharan African population susceptible to iNTS disease, in particular among children under the age of five [4]. In addition to these host risk factors, environmental risk factors for iNTS disease such as unimproved water sources contribute to the high incidence of iNTS disease in sub-Saharan Africa [7].

While existing studies provide estimates at the country, regional, and global levels mainly based on the incidence rates from population-based surveillance studies [1,2,8], they fail to provide geospatial heterogeneity of iNTS disease at the subnational level, which is crucial for prioritizing areas for targeted iNTS surveillance and control programs. We aimed to characterize the geospatial variation in probability of iNTS occurrence across sub-Saharan Africa (sSA) at a resolution of 20 km × 20 km using iNTS cases reported in the literature and geospatial data of host and environmental iNTS risk factors.

Methods

For peer review only

iNTS occurrence data

We sought data on iNTS occurrence and incidence rates in the peer-reviewed literature available in PubMed, Embase and Web of Science published from January 1, 2000 through June 30, 2021 in English language. We designed the search strings to account for each database and varying usage of the terms, non-typhoidal *Salmonella*, and to restrict the topic to human infections in sub-Saharan Africa while adapting to the syntax of different databases (Supplementary Material S1). We conducted the review according to the Preferred Reporting Items for Systematic Reviews and Meta Analyses (PRISMA) [9]. We used Rayyan [10] for a collaborative review of the literature and collated the data in Google sheet. The PRISMA flowchart shows an overview of the literature review process (**Figure 1**).

Three authors (BT, FF, and EP) reviewed the literature and collated the data with at least two independent reviewers reviewing each article. Disagreements between the reviewers were resolved after discussion with the reviewers and the first author (JHK). Studies were eligible regardless of the study type (e.g., outbreak investigation, population-based surveillance, or case report) if they reported original findings of human infection of iNTS with subnational location and year as a full text article. Conference proceedings were excluded. We excluded studies that used clinical indication (i.e., symptoms and signs), culture of a non-sterile site (e.g., stool), or serology to classify a case of iNTS. We excluded studies that did not report iNTS separately from *Salmonella* Typhi or Paratyphi A, B, or C. Abstracted variables include year, location, diagnostic method, and the number of cases. We defined the occurrence of iNTS as non-typhoidal *Salmonella* invasive disease confirmed through culture of samples taken from a normally sterile site (e.g., blood or bone marrow) with clear subnational location and year of occurrence. If observations spanned multiple years, the year of occurrence for each iNTS case was selected uniformly at random among observation years, which leads to the same number of cases for each year on average.

Geo-positioning of iNTS occurrence

iNTS occurrence was defined as a 20 km × 20 km grid cell for which the spatial resolution was chosen by taking into account the feasibility to explore subnational heterogeneity, computational cost, and a

For peer review only

previous study of the similar resolution [11]. The position was defined as a grid cell to which longitude and latitude of the area belongs based on the Google Maps (Alphabet Inc., Mountain View, California, US). If the record reported the subnational area in which iNTS was reported was smaller than a grid cell, we assumed that the iNTS disease occurred in the grid cell. On the other hand, if the subnational administrative units included more than one grid cell, we assumed that iNTS disease could occur on any of the grid cells within the boundary and used the covariate value averaged over the grid cells that fall on the area. If the occurrence was reported in a hospital, the grid cell for occurrence was selected randomly assuming that the probability that the iNTS disease appeared at d km from the hospital is de^{-kd}, where the rate of exponential decay k was chosen differentially according to the catchment area of the hospital. The exponential decay of the probability of was based on the idea that the frequency of visits to a hospital decreases with the distance (more generally known as distance decay) from the hospital following an exponential decay [12]. We assumed that the catchment area was 20 km and 100 km for primary and secondary/tertiary hospitals, respectively (i.e., k was 0.05 and 0.01 for primary and secondary/tertiary hospitals, respectively), which implies that 63% of the cases occurred within the boundary of 20 km and 100 km, respectively, and around 86% of the cases occurred within the boundary of 40 km and 200 km, respectively. We conducted a sensitivity analysis by assuming average distance of 50 km and 200 km for the iNTS occurrence location from the tertiary hospital (i.e., 0.02 or 0.005 for k), respectively.

<u>Geospatial covariates</u>

We explored the explanatory capacity for the iNTS occurrence of variables that may influence the risk of iNTS and cover the sub-Saharan African continent with at least 20 km × 20 km resolution. Covariates we explored included the prevalence of HIV [13] (**Figure S1**), *Plasmodium falciparum* [14] (**Figure S2**), prevalence of child growth failure [15,16] (e.g., underweight in **Figure S3**), and access to improved water sources (**Figure S4**) and sanitation facilities (**Figure S5**) [17]. This set of covariates was similar to that used to model the global burden of iNTS disease [1].

The HIV prevalence represented estimates of HIV prevalence among adults aged 15-45 years in sub-Saharan Africa, annually from 2000 through 2017, based on the review of various surveys including AIDS Indicator Survey, Demographic and Health Survey, Multiple Indicator Cluster Survey, Population-based HIV Impact Assessment Survey, and sentinel surveillance of women attending antenatal care clinics. The mean estimates were downloaded from the website for the Global Health Data Exchange (GHDx) [18]. The *P falciparum* parasite rate represents the proportion of children aged 2-10 years showing detectable *P. falciparum* parasite on a given year from 2000 through 2020, and estimated based on national surveys such as the Demographic and Health Survey data and the literature. The mean estimates were downloaded from the website for the Malaria Atlas Project [19]. Childhood growth failure measured the proportion of children with height-for-age, weight-for-height, and weight-for-age (expressed as stunting, wasting, and underweight, respectively) *z* score that was more than two standard deviations below the World Health Organization's median growth reference standards for a healthy population. The mean estimates for 2000-2017 were downloaded from the GHDx [20]. Access to improved drinking water source and access to sanitation facilities represent the proportion of population who have access to different levels of water and sanitation (e.g., access to piped water or access to improved water) from 2000 to 2017. The mean estimates were downloaded from GHDx [21]. All covariates were available at the resolution of 5 km by 5-km grids and were aggregated to 20-km by 20-km grids by computing the mean.

We matched the year of data collection and the year for geospatial covariates as closely as possible. All covariates were available for each year from 2000 to 2017 and were matched to occurrences observed in the same year. For occurrences observed before 2000, we used the covariates for the year 2000. For occurrences observed after 2017, we used the covariates for 2017 for water, sanitation, and childhood growth failure, as these covariates were only available up to 2017. However, we were able to use the covariates for Malaria and HIV through 2020, so we matched the years between the occurrences and

For peer review only

covariates for those covariates. Additionally, we matched the location of the data collection and the covariates at 20-km by 20-km grids.

Probability of occurrence

A boosted regression tree (BRT) modeling framework [22] was used to estimate probability of occurrence for iNTS based on the iNTS occurrence data, background data, and explanatory covariates. The BRT model integrates strengths of regression trees, where relationship between a response and predictors is modeled through recursive binary splits, and boosting, where many models are combined to improve predictive performance. The BRT model does not assume a linear relationship between predictor and response variables and can characterize complex interactions between covariates and response variables. Background data represented random grid cells which were sampled such that probability of being selected increased with decreasing travel time to the healthcare facilities based on the travel time to healthcare facilities [23]. This choice of background sampling strategy may mitigate a potential bias in our dataset that may overrepresent occurrences in areas near healthcare facilities. In the BRT, each regression tree includes only explanatory covariates that improve predictions when added over the existing regression trees.

We measured the relative importance of a covariate as proposed in the previous study [22] by measuring how often a variables is selected for splitting individuals trees, weighted by the squared improvement to the model as a result of each split, and averaged over all trees.

We grouped the covariates into five categories: HIV, malaria, child growth failure, water, and sanitation. Several variants (e.g., access to improved water source vs. access to piped water) were available for all categories except for HIV (**Table S1).** We chose one variable from each category that has the highest influence on the prediction of iNTS occurrence based on the relative importance of covariates from the model containing all covariates to improve interpretability considering that covariates in the same

For peer review only

category often have high correlation (e.g., *Plasmodium falciparum* parasite prevalence and incidence rate has Pearson's *r* of 0.95, see **Table S1** for details).

Model fitting and validation

Key parameters in the BRT model include learning rate (LR), tree complexity (TC), and the number of trees. LR, also known as the shrinkage parameter, determines the contribution of each tree to the growing model. The TC controls whether interactions are fitted with a model with a TC of one fitting an additive model, and a TC of two fitting a model with up to two-way interactions, and so on [22]. We determined the values of these two parameters (i.e., LR of 0.05 and TC of 5) through a grid search in which the number of tree was optimized through 10-fold cross-validation for each set of TC and LR by taking the model performance and computation time into account. For example, we chose TC of 5 over TC of 10 by considering increasing the TC to 10 improved prediction by 2% but the computation time increased by a factor of two. In the 10-fold cross-validation the dataset was randomly divided into 10 subsets and models were trained using 9 subsets and were tested against the 10th subset. This process was repeated for each of the 10 subsets and the number of trees was optimized by minimizing average prediction deviance. The set of hyperparameters with optimized number of regression trees that produced the lowest deviance was selected as the final model. We identified the final model for each of 400 different data sets.

The predictive capacity of the final model was evaluated by calculating the receiver operating characteristic (ROC) curve and an area under the ROC curve (AUC). The ROC curve shows the true positive ratio (i.e., proportion of correct predictions in predictions of positive class) against the false positive ratio (i.e., proportion of wrong predictions in predictions of negative class). The higher ROC curves indicate the model that made better prediction. The AUC is a one-number summary index indicating the area under ROC curve and in general is considered a better metric than the accuracy to measure the performance of a classifying model [24]. The models were implemented in statistical software R (version 4.1.3). Raster images were processed for modeling using raster package and the BRT

For peer review only

model were implemented using gbm and dismo packages. All figures were prepared using ggplot2 package. All the codes and datasets are available on GitHub [25].

Summarizing results

Because of the random nature of the occurrence data (e.g., location for the occurrence reported in the hospital was randomly assigned by the decay function), we created 20 occurrence data sets. For each of the occurrence data set, we created 20 different background data sets. Probability of occurrence (mean, 2.5th percentile, and 97.5th percentile) was predicted by summarizing these 400 BRT model simulations based on 400 different data set for every 20 km by 20 km grid cell. The probability of occurrence was aggregated to the administrative units (e.g., districts and provinces) as well as the 20 km by 20 km grid cells. The administrative unit areas were defined based on shapefiles from the Global Administrative Areas (GADM) website [26]. Estimates for the probability of occurrence was generated for each year from 2000 through 2017 using covariates for the corresponding year. We also generated predictions for years from 2018 through 2020 in which the malaria parasite rate was the only variable that have varying values by year while the other four variables were held constant at values observed in 2017.

Patient and Public Involvement

This study does not involve any human participation.

Results

We identified 131 unique articles reporting human iNTS infection confirmed through isolating NTS from blood, bone marrow, or cerebrospinal fluid. Years of publication ranged from 2000 to 2020 (**Figure 1**) while the study years ranged from 1979 through 2020. The number of reports were heterogeneous across year and country with overall 31,016 iNTS occurrences reported from 19 countries, and no report of iNTS occurrence in the rest 26 countries of sub-Saharan Africa, which does not necessarily mean that no occurrence (**Figure 2A**). Of iNTS occurrence reports, 25 (19%) were from Kenya, 25 (19%) from Malawi, 13 (10%) from Ghana, 10 (8%) from Tanzania, and 58 (44%) from other African countries.

For peer review only

Overall, 42,699 cases for which year and location were clearly identified were reported from 2000 through 2020 with variation by year and country (**Figure 2B**). Malawi was the most substantial contributor (*n*=19,075, 62%) followed by Kenya (*n*=3539, 11%), Mali (*n*=2215, 7%), and Mozambique (*n*=1947, 6%). A majority of studies (*n*=123, 94%) included blood culture for confirmation of iNTS and the next common diagnostic methods were culture of cerebrospinal fluid culture (*n*=14, 11%) followed by the less common diagnostic methods included culture of pleural (*n*=2, 2%) or peritoneal (*n*=1, <1%) fluids. Around 67% (*n*=88) of the occurrence reports came from the tertiary hospitals while about 13% (*n*=17) came from the primary hospitals and 20% (*n*=26) of the reports clearly indicated the number of administrative units from which the patients came. These reports led to on average 5,504 iNTS occurrences on 20 km by 20 km grids, representing around 3% of 180,490 grid cells comprising sub-Saharan Africa. A majority of occurrence focused on the same set of countries (i.e., Malawi, Kenya, Mali, and Mozambique) that contributed most in terms of the number of cases (**Figure 3**).

<u>Probability of iNTS occurrence</u>

Out of the 11 geospatial covariates that we explored, we selected five for the final model: *P falciparum* incidence rate, access to improved water, access to piped sanitation, prevalence of HIV, and prevalence of underweight (**Table S1**). The Pearson correlation coefficient (*r*) among those five covariates ranged from 0.08 to 0.57. The BRT model predicted well the occurrence of iNTS with the receiver operating characteristic (ROC) curve indicating an area under the ROC Curve (AUC) score of around 0.9 (**Figure S7**). Estimated probability of iNTS occurrence on 20 km by 20 km grids for 2017 showed significant spatial heterogeneity (**Figure 4A-4C**). The probability of occurrence of iNTS aggregated at the subregional level was highest in Middle Africa (0.34, 95% CI: 0.25 to 0.46) followed by Western Africa (0.33, 95% CI: 0.23 to 0.44), Eastern Africa (0.24, 95% CI: 0.17 to 0.33), and Southern Africa (0.08, 95% CI: 0.03 to 0.11) while high-risk grid cells appear to be scattered across the continent. Aggregated at the country level, probability of occurrence was highest at Malawi and lowest in Niger (**Table S2**).

For peer review only

Probability of occurrence averaged across administrative unit level 1 (i.e., province) showed similar geospatial variations (**Figure 4D**).

Contribution of covariates

Geospatial covariates showed varying contributions with underweight having the highest contribution (27.2% [95% CI: 26.2 to 28.1]) followed by HIV (23.8% [95% CI:23.1 to 24.5]), malaria (20.1% [95% CI:18.4 to 21.6]), sanitation (18.7% [95% CI:17.6 to 20.3]), and drinking water (10.2% [95% CI: 9.6 to 11.0]) (**Figure 5A**). As regression trees do not assume a linear relationship, the relationship between the probability of iNTS occurrence and the covariate can be complex (**Figure 5B**). The overall relationship between the probability of occurrence and the risk factors such as *P falciparum* incidence rate, HIV prevalence, and underweight prevalence appear to have a positive relationship.

Temporal heterogeneity of the probability of occurrence

Spatial distribution of the probability of occurrence varied by year. For 2000, probability of occurrence was predicted to be highest near Malawi with the country-aggregated value to be 0.88 [95% CI: 0.77 to 0.94]. The next was Mozambique (0.77; 95% CI: 0.62 to 0.87) followed by Uganda (0.65; 95% CI: 0.43 to 0.82) and Zimbabwe (0.62; 95% CI: 0.35 to 0.83) (**Figure 6A** and **6D**). In 2010, Mozambique (0.67; 95% CI: 0.47 to 0.83) had the highest probability of occurrence. Countries in Western Africa such as Ghana (0.64; 95% CI: 0.43 to 0.81), Guinea (0.56; ; 95% CI: 0.34 to 0.75), Guinea-Bissau (0.52; 95% CI: 0.33 to 0.71), and Liberia (0.49; 95% CI: 0.29 to 0.71) had high probability of occurrence, with the largest increase between 2000 and 2010 observed in Liberia (0.07 vs. 0.49) (**Figure 6A, 6B,** and **6D**). From 2000 to 2020, probability of occurrence aggregated at the country level increased most in Republic of the Congo (0.05 vs. 0.59) while decreased most in Uganda (0.65 vs. 0.23) (**Figure 6C and 6D**).

Discussion

We examined geospatial and temporal distribution of the probability of occurrence iNTS for sub-Saharan Africa on 20 km by 20 km grids using the data collated from an extensive literature review and high-

For peer review only

resolution geospatial covariates representing potential risk factors for iNTS disease. Estimated probability of iNTS occurrence showed substantial geospatial heterogeneity on 20 km by 20 km grid cells, subnational, and national levels and also temporal variation when measured by year. In 2017 for which most recent estimates for the modeled covariates were available, the estimated probability of occurrence was higher in Middle Africa and Western Africa and lower in Eastern Africa and Southern Africa.

Our study is the first to characterize the probability of iNTS occurrence at the sub-national levels. While predicted incidence rates at the national level are available [1], these predictions were based on a small number of surveillance studies. Having a way to identify sub-national high-risk areas using occurrence data as well as surveillance data is an important addition to the existing knowledge of iNTS epidemiology and particularly for designing effective and efficient intervention programs. Candidate vaccines for iNTS are under active development [27] and deployment strategies are critical for successful intervention and high return on investment. The current study can help identify areas of potential high burden and target for vaccination. Geospatial variation may vary over time (**Figure S8**, **Table S3**, and **Table S3**) and it will necessary to keep updating the estimates using more recent information. In a similar vein, this study can help identify areas for potential surveillance. While surveillance studies provide the most reliable disease occurrence and indidence data, they are resource-intensive, and it is important to conduct surveillance in the area that could provide the most valuable information such as the Middle Africa (e.g., Democratic Republic of the Congo and Republic of the Congo) in which the occurrence data are scarce while the probability of occurrence was high for years since round 2016 (**Figure 6D**, **Figure S8**, and **Table Sble S4**).

Our modeling analyses indicate that geospatial estimates of the host risk factors such as infection with HIV, malaria, or underweight have importance for predicting the geospatial distribution of iNTS occurrence while water and sanitation status may also have some smaller predictive capacity. In this regard, developing a risk index of iNTS based on a similar set of variables to what we have used has been attempted for nine sub-Saharan countries [28].

For peer review only

Our study had a number of limitations. First, the data points were few and especially we lacked data on the absence of iNTS disease. While occurrence data points greatly expanded those available from population-based studies, cumulative occurrences from 2000 through 2020 only represented around 3% of the 20 km by 20 km grids of sub-Saharan Africa. Second, the reported location of iNTS occurrence is restricted to hospital-level or high-level administrative units and does not accurately reflect the location of NTS transmission. Hence, the estimated probability of occurrence might not reflect the true probability of iNTS transmission. Our approach was to assign a catchment area to the hospital and assume that occurrence could happen on any of the grid cells according to a decay function representing decreasing probability of visits with increasing distance from the hospital. This creates a dataset that reflects more frequent occurrences in areas close to the hospital than in areas far away. To mitigate this potential bias, we selected background points biased towards shorter distance to healthcare facilities. While occurrences are randomly located based on the exponential decay function, the geospatial and temporal patterns appear to be robust against different parameterizations for the decay function (**Figure S9**). In addition, iNTS disease represents only a fraction of transmission of the pathogen [29] and thus it's likely that the estimates based on the disease will underestimate the true transmission of the disease. Third, the frequency of occurrence is likely to be influenced by the frequency of publication in that occurrence is only possible when they are reported in the peer-reviewed journal. Therefore, areas in which iNTS was not studied or reported for any reason will be underrepresented. Therefore, studies such as systematic surveillance will need to verify the inferred probability of occurrence. Fourth, while the BRT method is known to perform well in prediction, it is difficult to interpret the association between explanatory covariates and occurrence of iNTS (**Figure 5B**). However, the findings on the variables are generally consistent with our knowledge and we could still combine the knowledge on the probability of occurrence and the potential risk factors to prioritize our resources for more effective control of disease.

Conclusion

For peer review only

Our analysis suggests that the occurrence of iNTS disease varies greatly across sub-Saharan Africa, with certain regions being disproportionately affected. This geospatial heterogeneity is crucial to understand to effectively combat the iNTS disease. By identifying areas with high probability of occurrence, targeted surveillance can be conducted to better understand the incidence and the drivers of the disease in those regions. This information can then be used to develop intervention programs to prevent the spread of iNTS. Ultimately, a deeper understanding of the geographical distribution of iNTS can help to reduce its impact and improve public health outcomes in sub-Saharan Africa.

For peer review only

Funding

This project has received funding from the EU Horizon 2020 research and innovation programme under the project Vacc-iNTS (grant agreement number 815439).

Acknowledgement

We thank Vacc-iNTS team for technical support and continuous encouragement. Vacc-iNTS consortium collaborators include: Francis Agyapong (Kwame Nkrumah University of Science and Technology Kumasi), Francesco Berlanda Scorza (GSK Vaccines Institute for Global Health), Gianluca Breghi (Fondazione Achille Sclavo), Rocío Canals (GSK Vaccines Institute for Global Health), Melita A Gordon (University of Liverpool), Brama Hanumunthadu (University of Oxford), Samuel Kariuki (Kenya Medical Research Institute), Stefano Malvolti (MM Global Health Consulting), Carsten Mantel (MM Global Health Consulting), Donata Medaglini (Università di Siena and Sclavo Vaccines Association), Esther Muthumbi (KEMRI-Wellcome Trust Research Programme), Tonney S. Nyirenda (University of Malawi), Robert Onsare (Kenya Medical Research Institute), Ellis Owusu-Dabo (Kwame Nkrumah University of Science and Technology Kumasi), Maheshi Ramasamy (University of Oxford), J. Anthony Scott (KEMRI-Wellcome Trust Research Programme), Bassiahi Abdramane Soura (University of Ouagadougou), and Tiziana Spadafina (Sclavo Vaccines Association)

References

1. Stanaway JD, Parisi A, Sarkar K, Blacker BF, Reiner RC, Hay SI, et al. The global burden of non-typhoidal salmonella invasive disease: a systematic analysis for the Global Burden of Disease Study 2017. Lancet Infect Dis. 2019;19: 1312–1324. doi:10.1016/S1473-3099(19)30418-9

2. Marchello CS, Fiorino F, Pettini E, Crump JA, Vacc-iNTS Consortium Collaborators. Incidence of non-typhoidal Salmonella invasive disease: A systematic review and meta-analysis. J Infect. 2021;83: 523–532. doi:10.1016/j.jinf.2021.06.029

3. Marchello CS, Birkhold M, Crump JA, Vacc-iNTS consortium collaborators. Complications and mortality of non-typhoidal salmonella invasive disease: a global systematic review and meta-analysis. Lancet Infect Dis. 2022;22: 692–705. doi:10.1016/S1473-3099(21)00615-0

4. Crump JA, Sjölund-Karlsson M, Gordon MA, Parry CM. Epidemiology, Clinical Presentation, Laboratory Diagnosis, Antimicrobial Resistance, and Antimicrobial Management of Invasive Salmonella Infections. Clin Microbiol Rev. 2015;28: 901–937. doi:10.1128/CMR.00002-15

5. Gilchrist JJ, MacLennan CA. Invasive Nontyphoidal Salmonella Disease in Africa. EcoSal Plus. 2019;8. doi:10.1128/ecosalplus.ESP-0007-2018

6. Kingsley RA, Msefula CL, Thomson NR, Kariuki S, Holt KE, Gordon MA, et al. Epidemic multiple drug resistant Salmonella Typhimurium causing invasive disease in sub-Saharan Africa have a distinct genotype. Genome Res. 2009;19: 2279–2287. doi:10.1101/gr.091017.109

7. Chirwa EB, Dale H, Gordon MA, Ashton PM. What is the Source of Infections Causing Invasive Nontyphoidal Salmonella Disease? Open Forum Infectious Diseases. 2023;10: ofad086. doi:10.1093/ofid/ofad086

8. Ao TT, Feasey NA, Gordon MA, Keddy KH, Angulo FJ, Crump JA. Global burden of invasive nontyphoidal Salmonella disease, 2010(1). Emerg Infect Dis. 2015;21. doi:10.3201/eid2106.140999

9. Moher D, Liberati A, Tetzlaff J, Altman DG, PRISMA Group. Preferred reporting items for systematic reviews and meta-analyses: the PRISMA statement. PLoS Med. 2009;6: e1000097. doi:10.1371/journal.pmed.1000097

10. Ouzzani M, Hammady H, Fedorowicz Z, Elmagarmid A. Rayyan-a web and mobile app for systematic reviews. Syst Rev. 2016;5: 210. doi:10.1186/s13643-016-0384-4

11. Lessler J, Moore SM, Luquero FJ, McKay HS, Grais R, Henkens M, et al. Mapping the burden of cholera in sub-Saharan Africa and implications for control: an analysis of data across geographical scales. The Lancet. 2018;391: 1908–1915. doi:10.1016/S0140-6736(17)33050-7

12. Stock R. Distance and the utilization of health facilities in rural Nigeria. Soc Sci Med. 1983;17: 563–570. doi:10.1016/0277-9536(83)90298-8

For peer review only

13. Dwyer-Lindgren L, Cork MA, Sligar A, Steuben KM, Wilson KF, Provost NR, et al. Mapping HIV prevalence in sub-Saharan Africa between 2000 and 2017. Nature. 2019;570: 189–193. doi:10.1038/s41586-019-1200-9

14. Weiss DJ, Lucas TCD, Nguyen M, Nandi AK, Bisanzio D, Battle KE, et al. Mapping the global prevalence, incidence, and mortality of Plasmodium falciparum, 2000-17: a spatial and temporal modelling study. Lancet. 2019;394: 322–331. doi:10.1016/S0140-6736(19)31097-9

15. Uche IV, MacLennan CA, Saul A. A Systematic Review of the Incidence, Risk Factors and Case Fatality Rates of Invasive Nontyphoidal Salmonella (iNTS) Disease in Africa (1966 to 2014). Baker S, editor. PLoS Negl Trop Dis. 2017;11: e0005118. doi:10.1371/journal.pntd.0005118

16. Kinyoki DK, Osgood-Zimmerman AE, Pickering BV, Schaeffer LE, Marczak LB, Lazzar-Atwood A, et al. Mapping child growth failure across low- and middle-income countries. Nature. 2020;577: 231–234. doi:10.1038/s41586-019-1878-8

17. Deshpande A, Miller-Petrie MK, Lindstedt PA, Baumann MM, Johnson KB, Blacker BF, et al. Mapping geographical inequalities in access to drinking water and sanitation facilities in low-income and middle-income countries, 2000–17. Lancet Glob Health. 2020;8: e1162–e1185. doi:10.1016/S2214-109X(20)30278-3

18. Africa HIV Prevalence Geospatial Estimates 2000-2017. Available: https://ghdx.healthdata.org/record/ihme-data/africa-hiv-prevalence-geospatial-estimates-2000-2017

19. Malaria Atlas Project. Available: https://malariaatlas.org/

20. Low- and Middle-Income Country Child Growth Failure Geospatial Estimates 2000-2017. Available: https://ghdx.healthdata.org/record/ihme-data/lmic-child-growth-failure-geospatial-estimates-2000-2017

21. Low- and Middle-Income Country Drinking Water and Sanitation Facilities Access Geospatial Estimates 2000-2017. Available: https://ghdx.healthdata.org/record/ihme-data/lmic-wash-access-geospatial-estimates-2000-2017

22. Elith J, Leathwick JR, Hastie T. A working guide to boosted regression trees. Journal of Animal Ecology. 2008;77: 802–813. doi:10.1111/j.1365-2656.2008.01390.x

23. Weiss DJ, Nelson A, Vargas-Ruiz CA, Gligorić K, Bavadekar S, Gabrilovich E, et al. Global maps of travel time to healthcare facilities. Nat Med. 2020;26: 1835–1838. doi:10.1038/s41591-020-1059-1

24. Bradley AP. The use of the area under the ROC curve in the evaluation of machine learning algorithms. Pattern Recognition. 1997;30: 1145–1159. doi:10.1016/S0031-3203(96)00142-2

25. Kim J-H. Geospatial distribution of invasive non-typhoidal Salmonella (iNTS) disease occurrence in sub-Saharan Africa. Available: https://github.com/kimfinale/iNTS_Mapping

26. Global Administrative Areas. [cited 1 Feb 2022]. Available: www.gadm.org

For peer review only

For peer review only - http://bmjopen.bmj.com/site/about/guidelines.xhtml

27. GlaxoSmithKline. A Phase 1/2a, Observer-blind, Randomized, Controlled, Two-stage, Multi-country Study to Evaluate the Safety, Reactogenicity, and Immune Response of the Trivalent Vaccine Against Invasive Nontyphoidal Salmonella (iNTS) and Typhoid Fever in Healthy European and African Adults. clinicaltrials.gov; 2022 Oct. Report No.: NCT05480800. Available: https://clinicaltrials.gov/ct2/show/NCT05480800

28. Lee J-S, Mogasale V, Marks F, Kim J. Geographical distribution of risk factors for invasive non-typhoidal Salmonella at the subnational boundary level in sub-Saharan Africa. BMC Infectious Diseases. 2021;21: 529. doi:10.1186/s12879-021-06198-1

29. Invasive multidrug-resistant non-typhoidal Salmonella infections in Africa: zoonotic or anthroponotic transmission? In: ScienceOpen [Internet]. [cited 7 Apr 2023]. doi:10.1099/jmm.0.46375-0

For peer review only

Figure 1. PRISMA flow diagram for the systematic review of geospatial distribution of invasive non-typhoidal Salmonella (iNTS) occurrence in sub-Saharan Africa, 2000-2020.

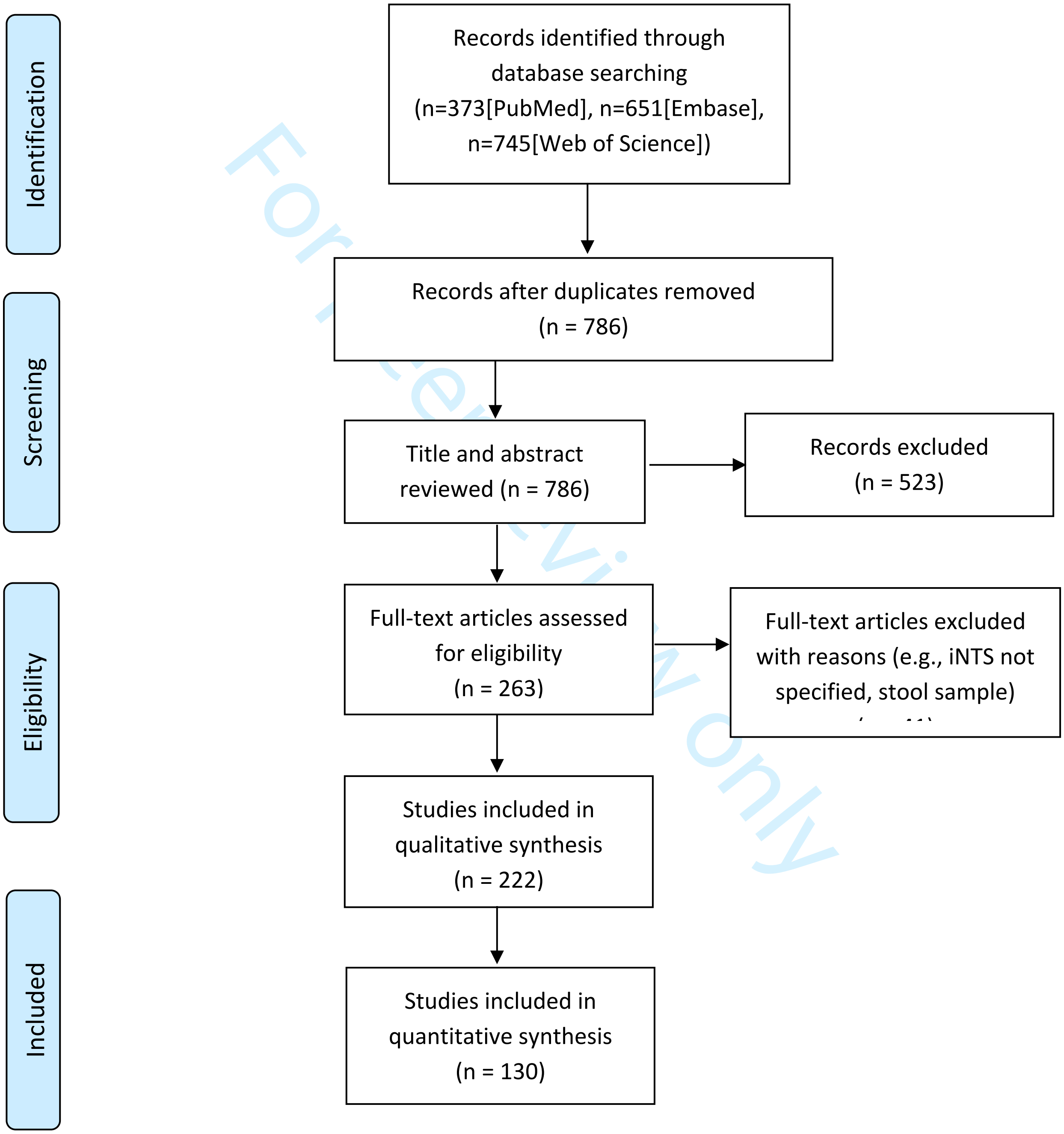

For peer review only

Figure 2. Data of human iNTS infection, sub-Saharan Africa, 2000-2020. (A) represents the number of reports by country and year of publication. (B) represents the number of reported cases by country and year of data collection. CAR = Central African Republic; DR Congo = Democratic Republic of the Congo

A

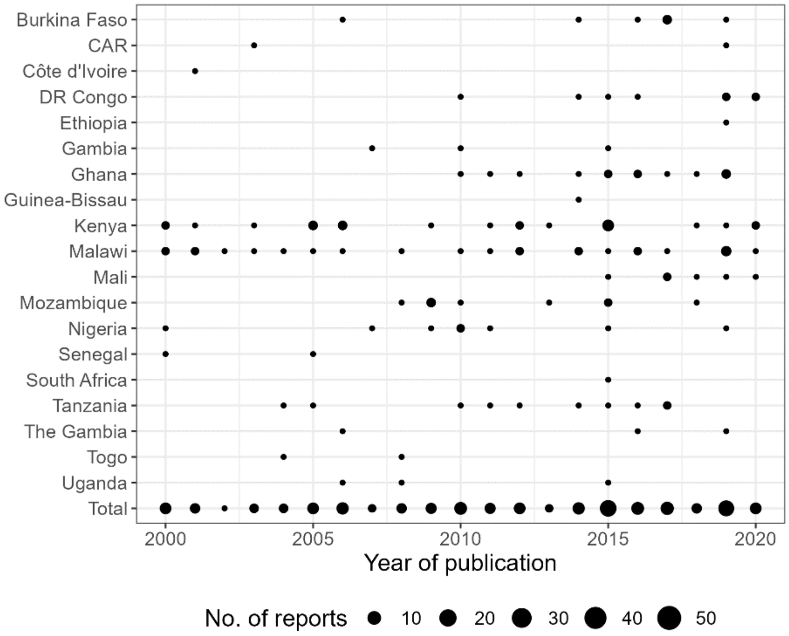

B

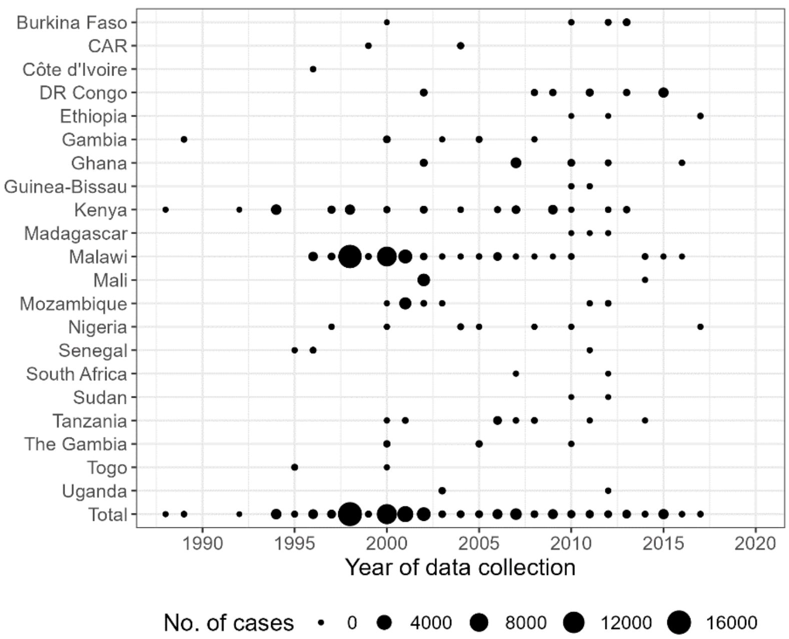

Figure 3. iNTS Occurrence on 20 km by 20 km grids. (A) and (B) represent typical occurrences observed in 2010 and during the entire search period (2000-2020), respectively. Thick and thin lines indicate borders of the administrative unit level 0 (i.e., country) and the administrative unit level 1 (i.e., province).

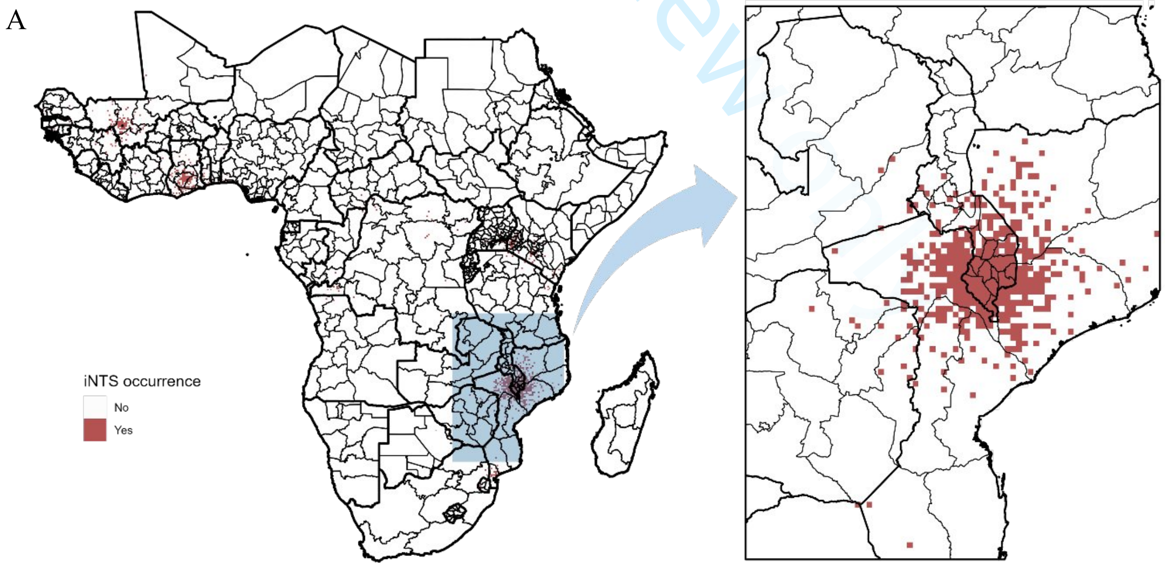

B

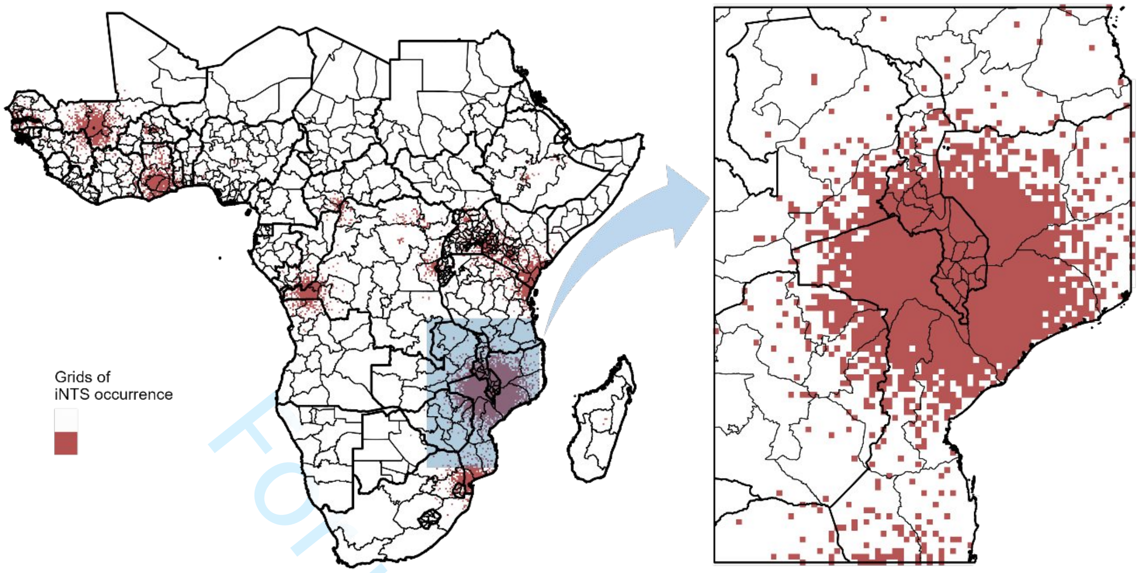

Figure 4. Estimated probability of iNTS disease occurrence, sub-Saharan Africa, 2017. Thick and thin lines indicate borders of country and province, respectively. (A), (B), and (C) show mean, 2.5th and 97.5th values on 20 km by 20 km grids. (D) shows the median values aggregated at the administrative unit level 1 (i.e., province or districts).

A

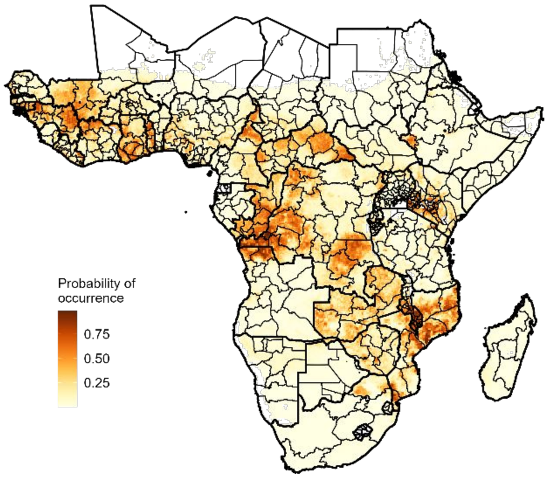

B

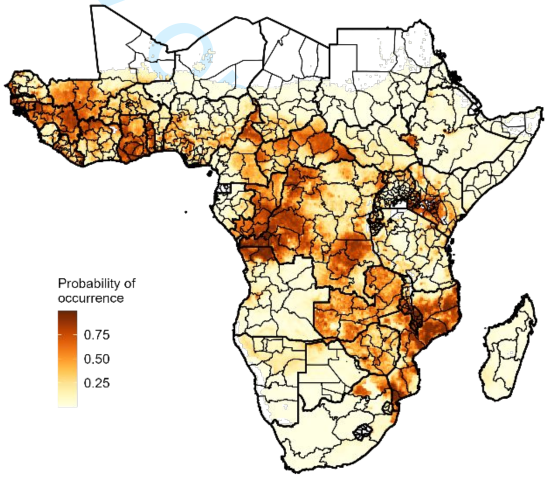

C

D

For peer review only

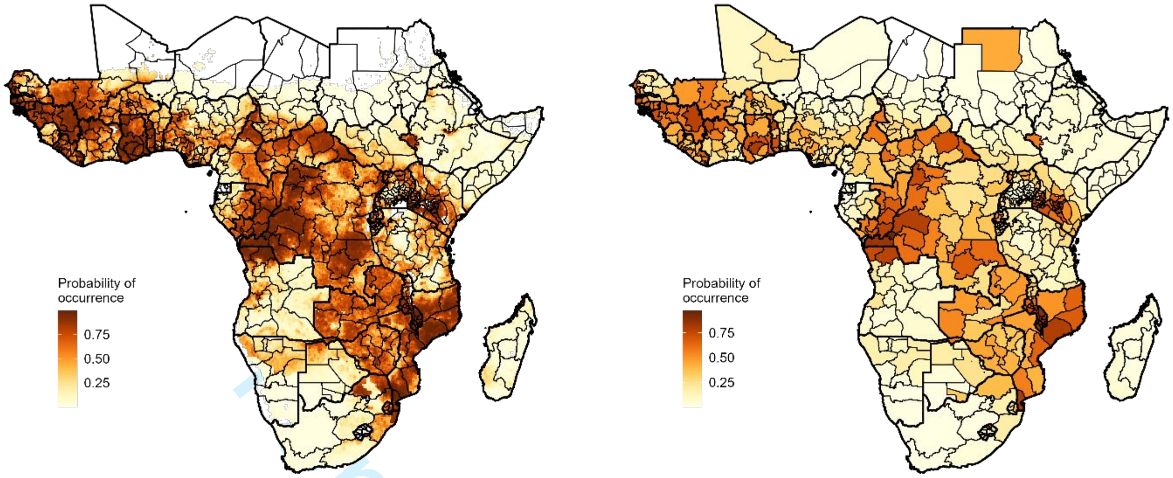

Figure 5. Impact of covariates on the probability of occurrence. Results are based on 400 simulation runs. (A) represents relative importance of covariates on the probability of occurrence. Bar plots and error bars indicate mean with 95% confidence intervals. (B) represents probability of occurrence in response to variables. Smoothed line (blue) and 95% confidence interval (red) bands were based on LOESS (LOcally Estimated Scatterplot Smoothing).

A

For peer review only

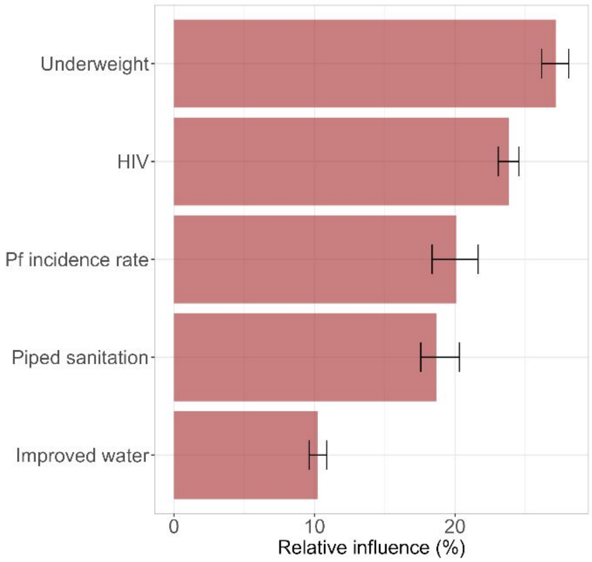
Underweight
HIV
Pf incidence rate
Piped sanitation
Improved water
0
10
20
Relative influence (%)

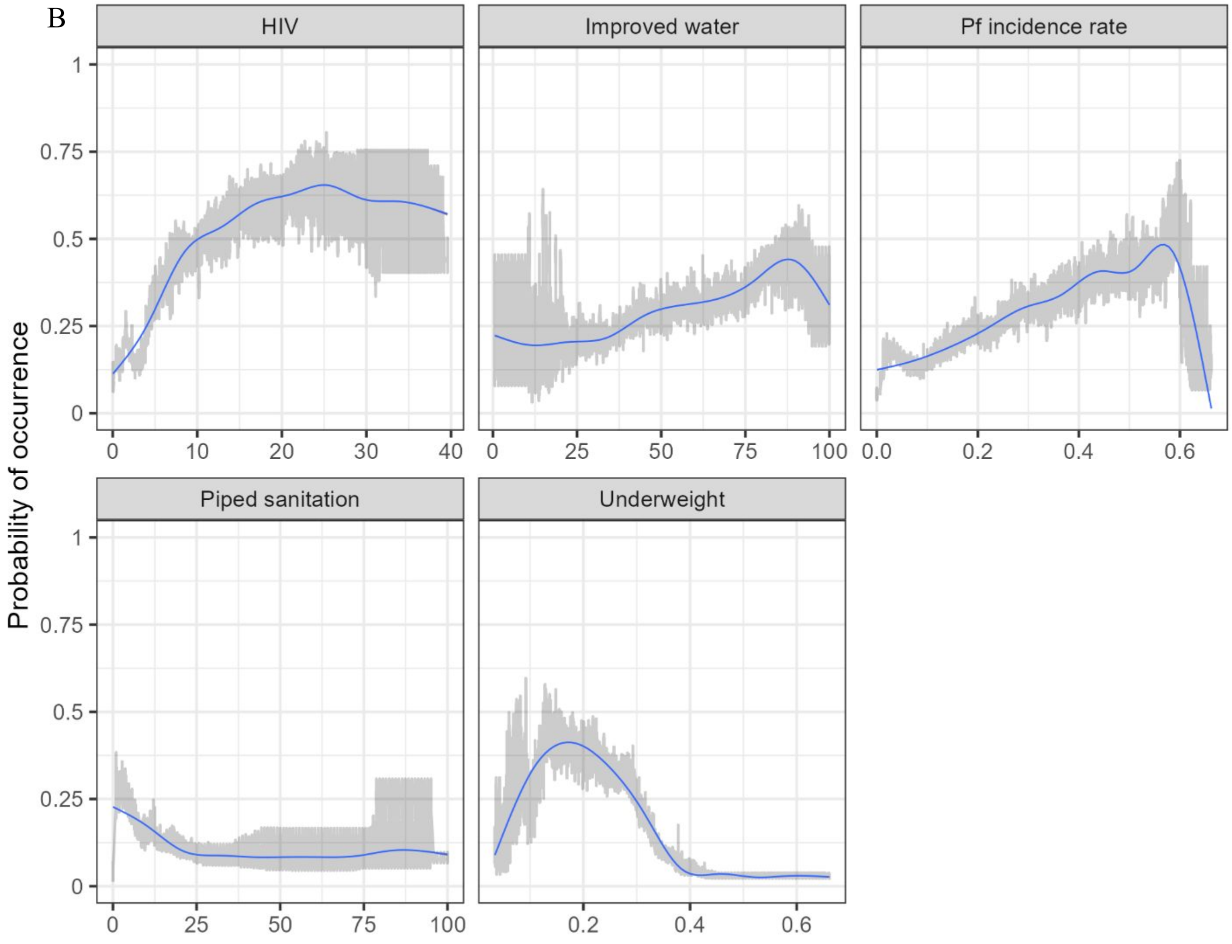
B
HIV
Improved water
Pf incidence rate
Piped sanitation
Underweight
Probability of occurrence
0
0.25
0.5
0.75
1
0
10
20
30
40
25
50
75
100
0.0
0.2
0.4
0.6

Figure 6. Probability of occurrence at the 20 km by 20 km grid level and averaged across the country level. (A), (B), and (C) represent the mean probability of occurrence for 2000, 2010, and 2020, respectively. (D) represents the values aggregated at the country level for 2000-2020. CAR=Central African Republic; DR Congo = Democratic Republic of the Congo; Congo=Republic of the Congo

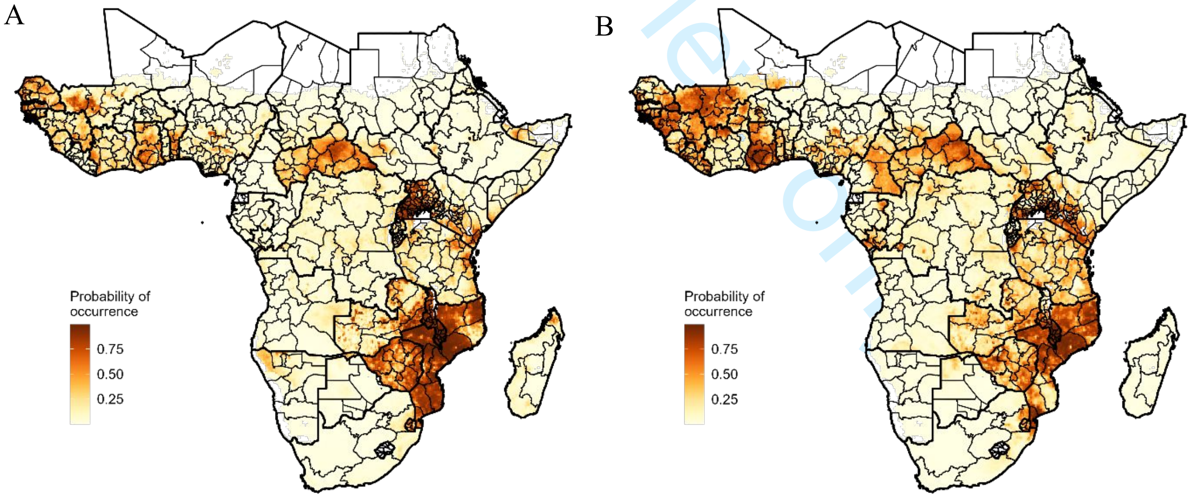

For peer review only

C

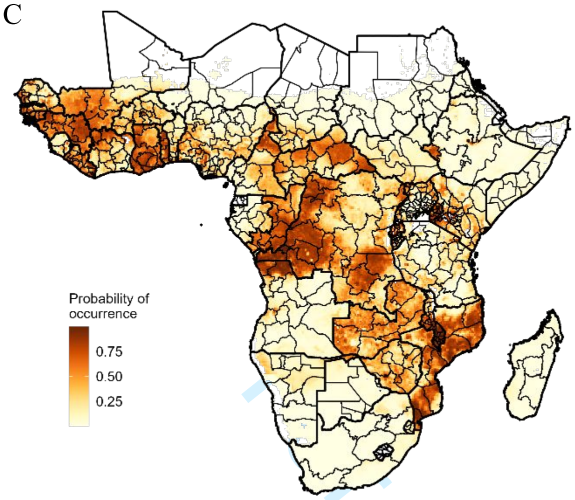

D

475

For peer review only

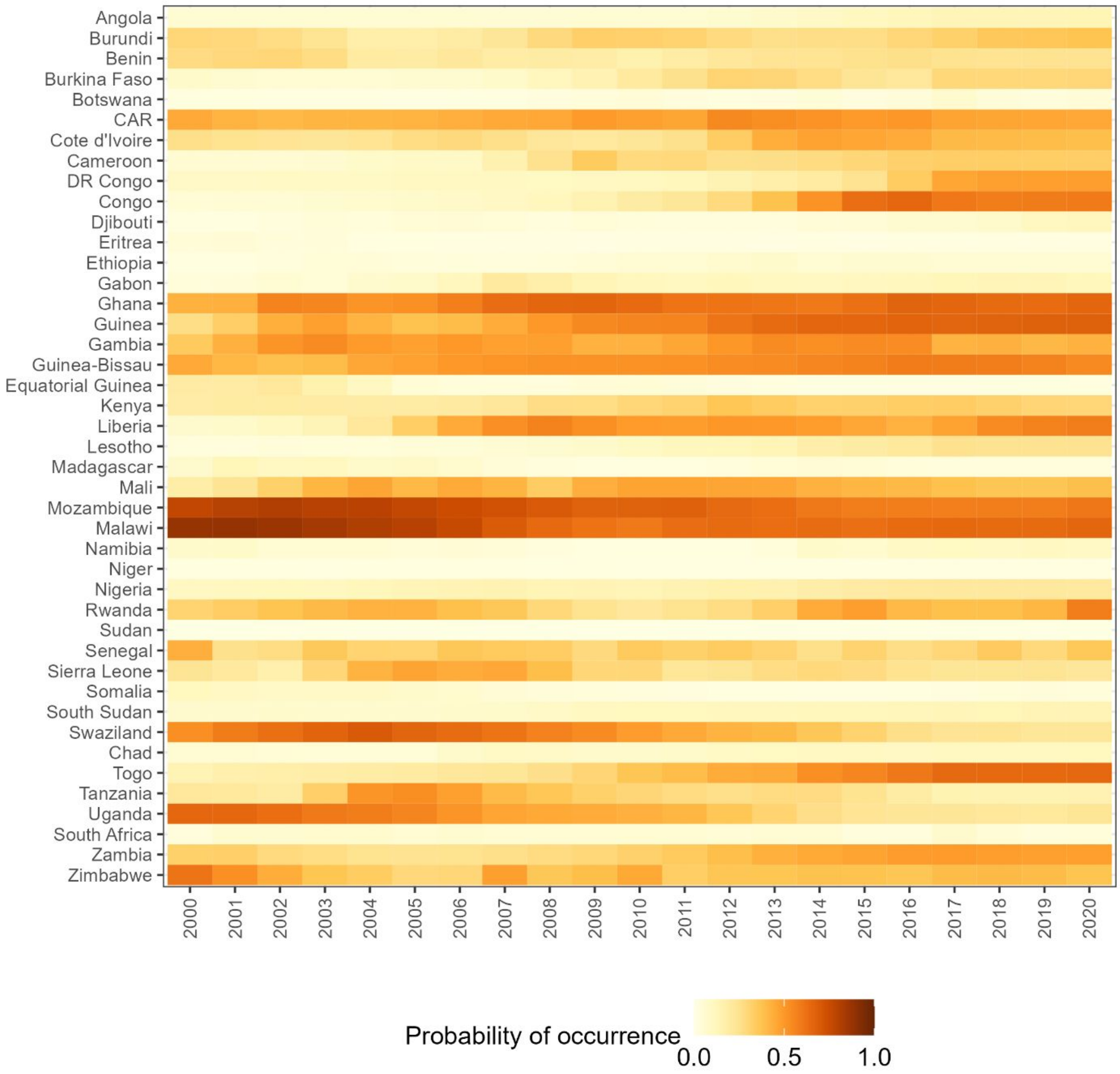
Angola
Burundi
Benin
Burkina Faso
Botswana
CAR
Cote d'Ivoire
Cameroon
DR Congo
Congo
Djibouti
Eritrea
Ethiopia
Gabon
Ghana
Guinea
Gambia
Guinea-Bissau
Equatorial Guinea
Kenya
Liberia
Lesotho
Madagascar
Mali
Mozambique
Malawi
Namibia
Niger
Nigeria
Rwanda
Sudan
Senegal
Sierra Leone
Somalia
South Sudan
Swaziland
Chad
Togo
Tanzania
Uganda
South Africa
Zambia
Zimbabwe
2000 2001 2002 2003 2004 2005 2006 2007 2008 2009 2010 2011 2012 2013 2014 2015 2016 2017 2018 2019 2020
Probability of occurrence
0.0 0.5 1.0

476

477

Supplementary Material

S1. Search terms

1. PubMed

(((nontyph*[Tiab] OR non-typh*[Tiab] OR group[Tiab] OR typhimurium[Tiab] OR enteritidis[Tiab] OR Heidelberg[Tiab] OR Dublin[Tiab] OR Choleraesuis[Tiab] OR Newport[Tiab] OR Virchow[Tiab] OR Concord[Tiab] OR Brancaster[Tiab] OR freetown[Tiab] OR Infantis[Tiab] OR Isangi[Tiab]) AND (salmonella[Tiab] OR salmonellosis[Tiab])) OR iNTS[Tiab])

AND

("Bacteremia"[Mesh] OR bacteremia [Tiab] OR bacteraemia [Tiab] OR "Sepsis"[Mesh] OR sepsis [Tiab] OR septic*[Tiab] OR "Invasive"[Tiab] OR bloodstream*[Tiab] OR “blood culture”[Tiab] OR “blood-culture”[Tiab] OR hemoculture OR incidence OR epidemiolog* OR burden OR case OR infect* OR prevalen*[Tiab])

AND

hasabstract

AND

“Humans”[Mesh]

AND

("2000/01/01"[Dp] : "2020/12/31"[Dp])

AND

("Africa South of the Sahara"[Mesh] OR "sub-saharan Africa"[Tiab] OR "subsaharan Africa"[Tiab] OR "Sub Saharan Africa"[Tiab] OR "Africa, Central"[Mesh] OR "Central Africa"[Tiab] OR "Cameroon"[Mesh] OR "Cameroon"[Tiab] OR "Central African Republic"[Mesh] OR "Central African Republic"[Tiab] OR "Chad"[Mesh] OR "Chad"[Tiab] OR "Congo"[Mesh] OR "Congo"[Tiab] OR "Democratic Republic of the Congo"[Mesh] OR "Democratic Republic of the Congo"[Tiab] OR "Equatorial Guinea"[Mesh] OR "Equatorial Guinea"[Tiab] OR "Gabon"[Mesh] OR "Gabon"[Tiab] OR "Sao Tome and Principe"[Mesh] OR "Sao Tome and Principe"[Tiab] OR "Africa, Eastern"[Mesh] OR "Eastern Africa"[Tiab] OR "East Africa"[Tiab] OR "Burundi"[Mesh] OR "Burundi"[Tiab] OR "Djibouti"[Mesh] OR "Djibouti"[Tiab] OR "Eritrea"[Mesh] OR "Eritrea"[Tiab] OR "Ethiopia"[Mesh] OR "Ethiopia" [Tiab] OR "Kenya"[Mesh] OR "Kenya" [Tiab] OR "Rwanda"[Mesh] OR "Rwanda" [Tiab] OR "Somalia"[Mesh] OR "Somalia"[Tiab] OR "South Sudan"[Mesh] OR "South Sudan"[Tiab] OR "Sudan"[Mesh] OR "Sudan"[Tiab] OR "Tanzania"[Mesh] OR "Tanzania"[Tiab] OR "Uganda"[Mesh] OR "Uganda"[Tiab] OR "Africa,

For peer review only

Southern"[Mesh] OR "Southern Africa"[Tiab] OR "South Africa"[Tiab] OR "Angola"[Mesh] OR "Angola"[Tiab] OR "Botswana"[Mesh] OR "Botswana"[Tiab] OR "Lesotho"[Mesh] OR "Lesotho"[Tiab] OR "Malawi"[Mesh] OR "Malawi"[Tiab] OR "Mozambique"[Mesh] OR "Mozambique"[Tiab] OR "Namibia"[Mesh] OR "Namibia"[Tiab] OR "South Africa"[Mesh] OR "South Africa"[Tiab] OR "Swaziland"[Mesh] OR "Swaziland"[Tiab] OR "Zambia"[Mesh] OR "Zambia"[Tiab] OR "Zimbabwe"[Mesh] OR "Zimbabwe"[Tiab] OR "Africa, Western"[Mesh] OR "Western Africa"[Tiab] OR "West Africa"[Tiab] OR "Benin"[Mesh] OR "Benin"[Tiab] OR "Burkina Faso"[Mesh] OR "Burkina Faso"[Tiab] OR "Cabo Verde"[Mesh] OR "Cabo Verde"[Tiab] OR "Cote d'Ivoire"[Mesh] OR "Cote d'Ivoire"[Tiab] OR "Gambia"[Mesh] OR "Gambia"[Tiab] OR "Ghana"[Mesh] OR "Ghana"[Tiab] OR "Guinea"[Mesh] OR "Guinea"[Tiab] OR "Guinea-Bissau"[Mesh] OR "Guinea-Bissau"[Tiab] OR "Liberia"[Mesh] OR "Liberia"[Tiab] OR "Mali"[Mesh] OR "Mali"[Tiab] OR "Mauritania"[Mesh] OR "Mauritania"[Tiab] OR "Niger"[Mesh] OR "Niger"[Tiab] OR "Nigeria"[Mesh] OR "Nigeria"[Tiab] OR "Senegal"[Mesh] OR "Senegal"[Tiab] OR "Sierra Leone"[Mesh] OR "Sierra Leone"[Tiab] OR "Togo"[Mesh] OR "Togo"[Tiab] OR "Comoros"[Mesh] OR "Comoros"[Tiab] OR "Mayotte"[Tiab] OR "Madagascar"[Mesh] OR "Madagascar"[Tiab] OR "Sahel"[Tiab])

2. Embase

(((‘nontyph*’:ti,ab OR ‘non-typh*’:ti,ab OR ‘group’:ti,ab OR ‘typhimurium’:ti,ab OR ‘enteritidis’:ti,ab OR ‘Heidelberg’:ti,ab OR ‘Dublin’:ti,ab OR ‘Choleraesuis’:ti,ab OR ‘Newport’:ti,ab OR ’Virchow’:ti,ab OR ‘Concord’:ti,ab OR ‘Brancaster’:ti,ab OR ‘freetown’:ti,ab OR ‘Infantis’:ti,ab OR ‘Isangi’:ti,ab) AND (‘salmonella’:ti,ab OR ‘salmonellosis’:ti,ab)) OR ‘iNTS’:ti,ab)

AND

(‘bacteremia’:ti,ab OR ‘sepsis’:ti,ab OR ‘septic*’:ti,ab OR ‘Invasive’:ti,ab OR ‘bloodstream*’:ti,ab OR ‘blood culture’:ti,ab OR ‘blood-culture’:ti,ab OR ‘hemoculture’:ti,ab OR ‘incidence’:ti,ab OR ‘epidemiolog*’:ti,ab OR ‘burden’:ti,ab OR ‘case’:ti,ab OR ‘infect*’:ti,ab OR ‘prevalen*’:ti,ab)

AND

[2000-2020]/py

AND

'Africa south of the Sahara'/exp OR ‘Sub-Saharan Africa’:ti,ab OR ‘Subsaharan Africa’:ti,ab OR ‘Sub Saharan Africa’:ti,ab OR 'Central Africa'/exp OR 'Central Africa':ti,ab OR 'Cameroon'/exp OR 'Cameroon':ti,ab OR 'Central African Republic'/exp OR 'Central African Republic':ti,ab OR 'Chad'/exp OR 'Chad':ti,ab OR 'Congo'/exp OR 'Congo':ti,ab OR 'Democratic Republic Congo'/exp OR 'Democratic Republic Congo':ti,ab OR 'Equatorial Guinea'/exp OR 'Equatorial Guinea':ti,ab OR 'Gabon'/exp OR 'Gabon':ti,ab OR 'Sao Tome and Principe'/exp OR 'Sao Tome and Principe':ti,ab OR ‘Eastern Africa’:ti,ab OR ‘East Africa’:ti,ab OR 'Burundi'/exp OR 'Burundi':ti,ab OR 'Djibouti'/exp OR 'Djibouti':ti,ab OR 'Eritrea'/exp OR 'Eritrea':ti,ab OR 'Ethiopia'/exp OR 'Ethiopia':ti,ab OR 'Kenya'/exp OR 'Kenya':ti,ab OR 'Rwanda'/exp OR 'Rwanda':ti,ab OR 'Somalia'/exp OR 'Somalia':ti,ab OR 'South Sudan'/exp OR 'South

For peer review only

Sudan':ti,ab OR 'Sudan'/exp OR 'Sudan':ti,ab OR 'Tanzania'/exp OR 'Tanzania':ti,ab OR 'Uganda'/exp OR 'Uganda':ti,ab OR ‘Southern Africa’:ti,ab OR ‘South Africa’:ti,ab OR 'Angola'/exp OR 'Angola':ti,ab OR 'Botswana'/exp OR 'Botswana':ti,ab OR 'Lesotho'/exp OR 'Lesotho':ti,ab OR 'Malawi'/exp OR 'Malawi':ti,ab OR 'Mozambique'/exp OR 'Mozambique':ti,ab OR 'Namibia'/exp OR 'Namibia':ti,ab OR 'South Africa'/exp OR 'South Africa':ti,ab OR 'Swaziland'/exp OR 'Swaziland':ti,ab OR 'Zambia'/exp OR 'Zambia':ti,ab OR 'Zimbabwe'/exp OR 'Zimbabwe':ti,ab OR ‘Western Africa’:ti,ab OR ‘West Africa’:ti,ab OR 'Benin'/exp OR 'Benin':ti,ab OR 'Burkina Faso'/exp OR 'Burkina Faso':ti,ab OR 'Cape Verde'/exp OR 'Cape Verde':ti,ab OR 'Cote d`Ivoire'/exp OR 'Cote d`Ivoire':ti,ab OR 'Gambia'/exp OR 'Gambia':ti,ab OR 'Ghana'/exp OR 'Ghana':ti,ab OR 'Guinea'/exp OR 'Guinea':ti,ab OR 'Guinea-Bissau'/exp OR 'Guinea-Bissau':ti,ab OR 'Liberia'/exp OR 'Liberia':ti,ab OR 'Mali'/exp OR 'Mali':ti,ab OR 'Mauritania'/exp OR 'Mauritania':ti,ab OR 'Niger'/exp OR 'Niger':ti,ab OR 'Nigeria'/exp OR 'Nigeria':ti,ab OR 'Senegal'/exp OR 'Senegal':ti,ab OR 'Sierra Leone'/exp OR 'Sierra Leone':ti,ab OR 'Togo'/exp OR 'Togo':ti,ab OR 'Comoros'/exp OR 'Comoros':ti,ab OR 'Mayotte'/exp OR 'Mayotte':ti,ab OR 'Madagascar'/exp OR 'Madagascar':ti,ab OR 'Sahel'/exp OR 'Sahel':ti,ab

3. Web of Science

(AB=(((nontyph* OR non-typh* OR group OR typhimurium OR enteritidis OR Heidelberg OR Dublin OR Choleraesuis OR Newport OR Virchow OR Concord OR Brancaster OR freetown OR Infantis OR Isangi) AND (salmonella OR salmonellosis)) OR iNTS)

AND

AB=(bacteremia OR sepsis OR septic* OR Invasive OR bloodstream* OR blood culture OR blood-culture OR hemoculture OR incidence OR epidemiolog* OR burden OR case OR infect* OR prevalen*)

AND

AB=(“Sub-Saharan Africa” OR “Subsaharan Africa” OR “Sub Saharan Africa” OR “Central Africa” OR Cameroon OR “Central African Republic” OR Chad OR Congo OR “Democratic Republic Congo” OR “Equatorial Guinea” OR Gabon OR “Sao Tome and Principe” OR “Eastern Africa” OR “East Africa” OR Burundi OR Djibouti OR Eritrea OR Ethiopia OR Kenya OR Rwanda OR Somalia OR “South Sudan” OR Sudan OR Tanzania OR Uganda OR “Southern Africa” OR “South Africa” OR Angola OR Botswana OR Lesotho OR Malawi OR Mozambique OR Namibia OR Swaziland OR Zambia OR Zimbabwe OR “Western Africa” OR “West Africa” OR Benin OR Burkina Faso OR “Cape Verde” OR “Cote d`Ivoire” OR Gambia OR Ghana OR Guinea OR “Guinea-Bissau” OR Liberia OR Mali OR Mauritania OR Niger OR Nigeria OR Senegal OR “Sierra Leone” OR Togo OR Comoros OR Mayotte OR Madagascar OR Sahel))

OR

(TI=(((nontyph* OR non-typh* OR group OR typhimurium OR enteritidis OR Heidelberg OR Dublin OR Choleraesuis OR Newport OR Virchow OR Concord OR Brancaster OR freetown OR Infantis OR Isangi) AND (salmonella OR salmonellosis)) OR iNTS)

AND

TI=(bacteremia OR sepsis OR septic* OR Invasive OR bloodstream* OR blood culture OR blood-culture OR hemoculture OR incidence OR epidemiolog* OR burden OR case OR infect* OR prevalen*)

AND

TI=("Sub-Saharan Africa" OR "Subsaharan Africa" OR "Sub Saharan Africa" OR "Central Africa" OR Cameroon OR "Central African Republic" OR Chad OR Congo OR "Democratic Republic Congo" OR "Equatorial Guinea" OR Gabon OR "Sao Tome and Principe" OR "Eastern Africa" OR "East Africa" OR Burundi OR Djibouti OR Eritrea OR Ethiopia OR Kenya OR Rwanda OR Somalia OR "South Sudan" OR Sudan OR Tanzania OR Uganda OR "Southern Africa" OR "South Africa" OR Angola OR Botswana OR Lesotho OR Malawi OR Mozambique OR Namibia OR Swaziland OR Zambia OR Zimbabwe OR "Western Africa" OR "West Africa" OR Benin OR Burkina Faso OR "Cape Verde" OR "Cote d`Ivoire" OR Gambia OR Ghana OR Guinea OR "Guinea-Bissau" OR Liberia OR Mali OR Mauritania OR Niger OR Nigeria OR Senegal OR "Sierra Leone" OR Togo OR Comoros OR Mayotte OR Madagascar OR Sahel))

S2. Explanatory covariates

For peer review only

Figure S1. Mean estimates for the prevalence of HIV infection among 15-45 year olds in sub-Saharan Africa in 2017 [1].

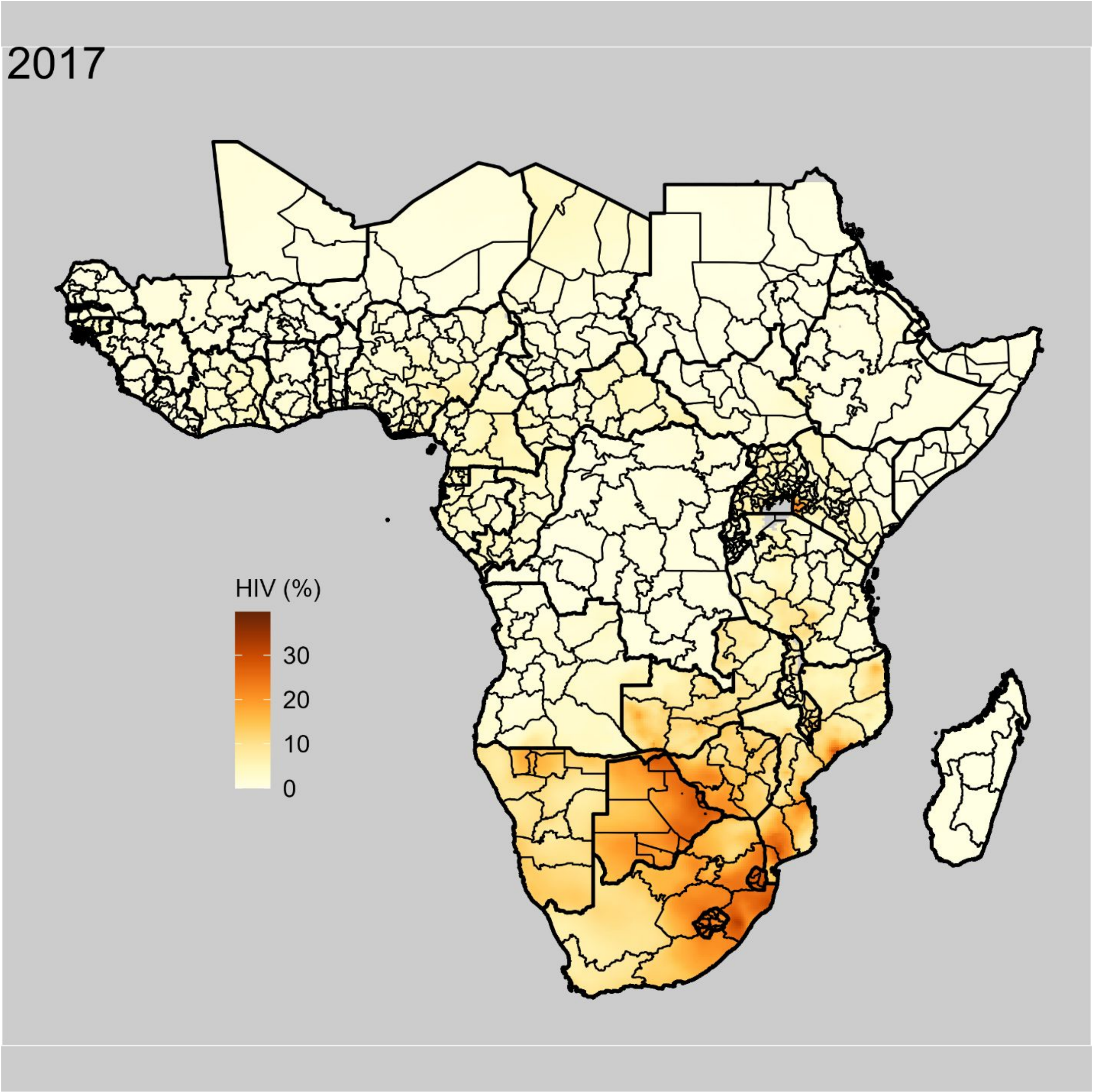

Figure S2. Incidence rate of *Plasmodium Falciparum* [2], 2017. The values indicate number of newly diagnosed Plasmodium falciparum cases per person in 2017.

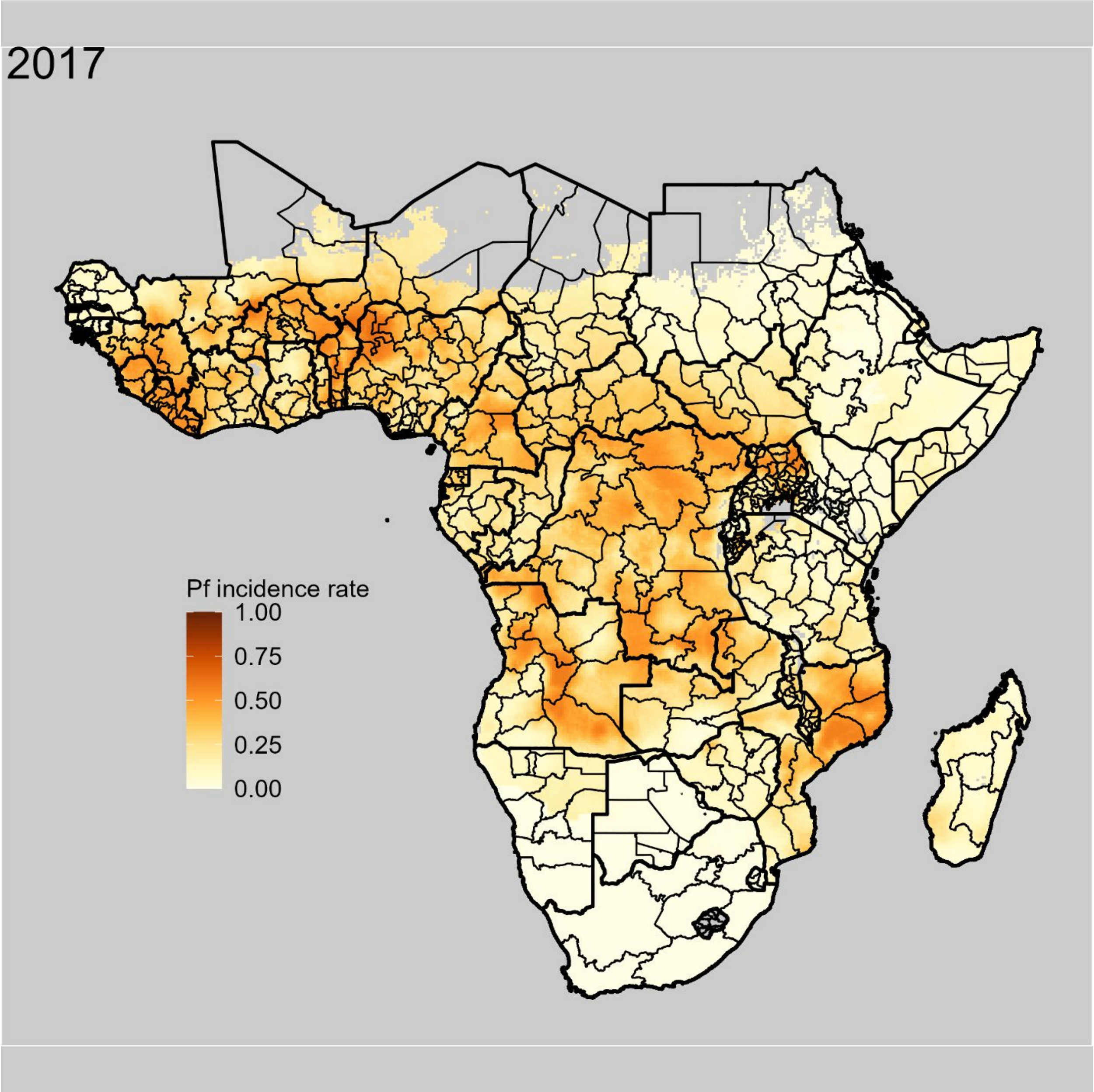

Figure S3. Prevalence of underweight [3].

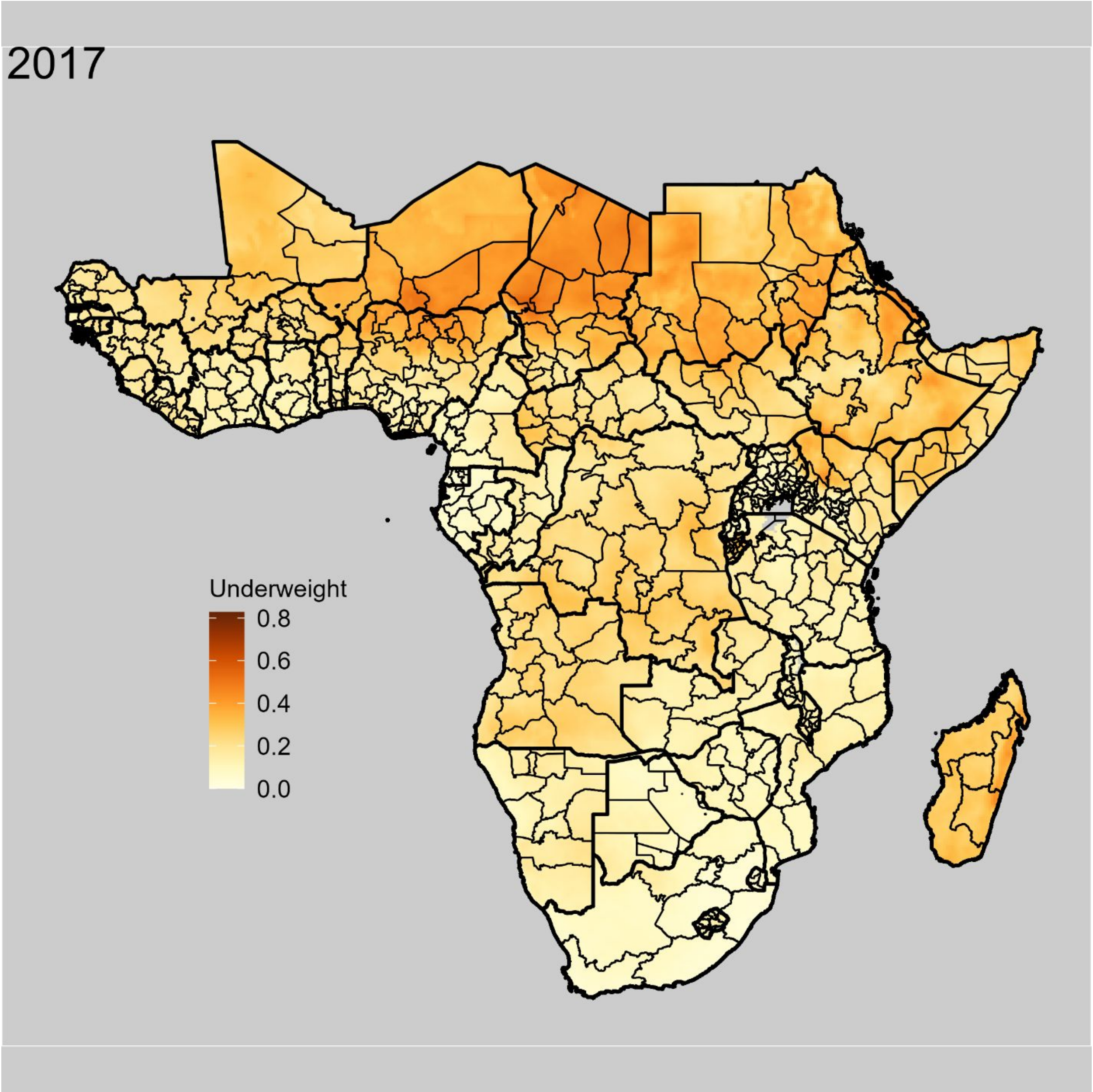

Figure S4. Percentage access to improved drinking water source. The values are based on the estimates on the mean access to any improved water sources on 5 km by 5 km grids [4]

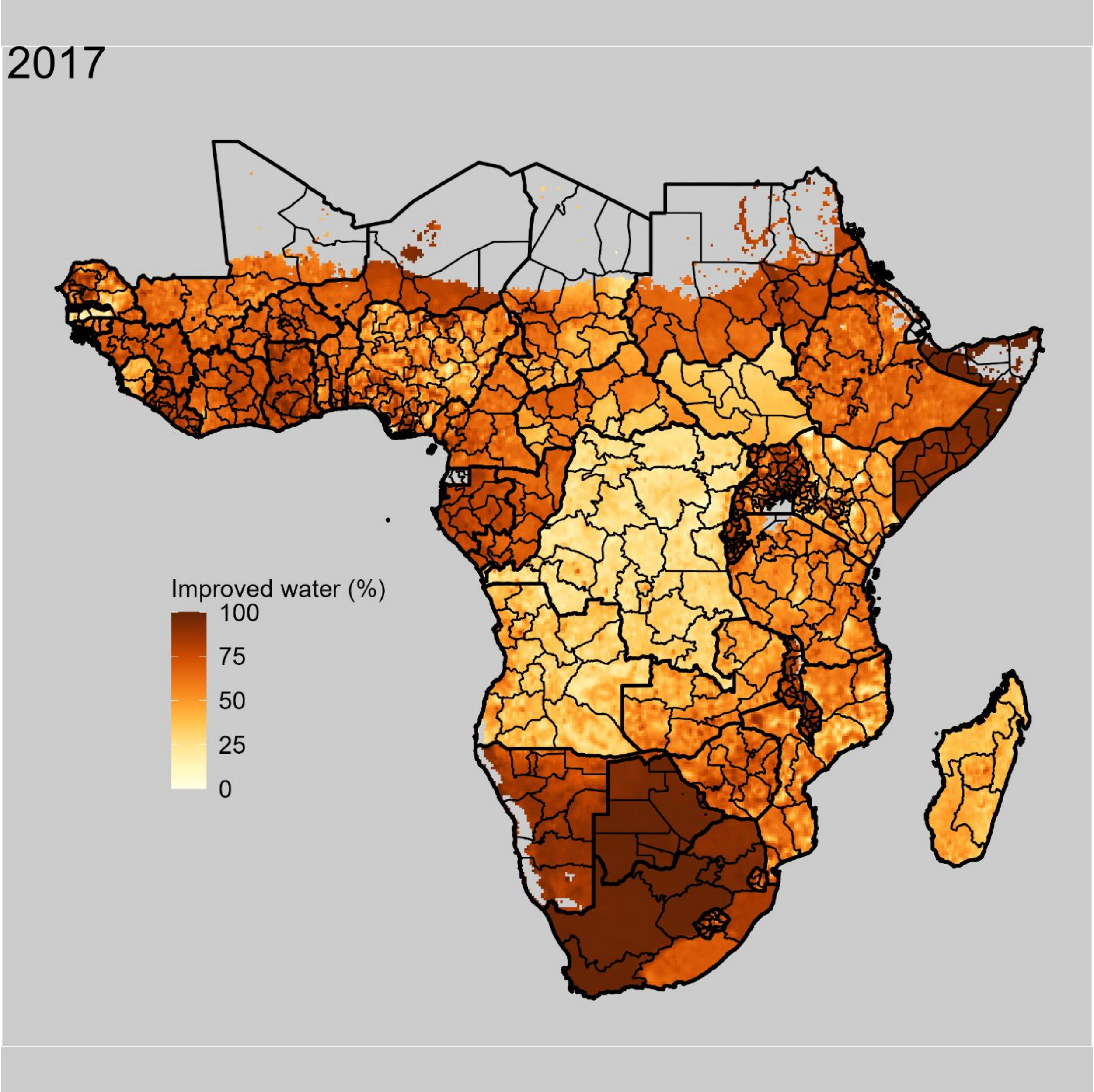

Figure S5. Percentage access to improved sanitation facilities. The values are based on the recent estimates on the mean access to any improved sanitation facilities on 5 km by 5 km grids [4].

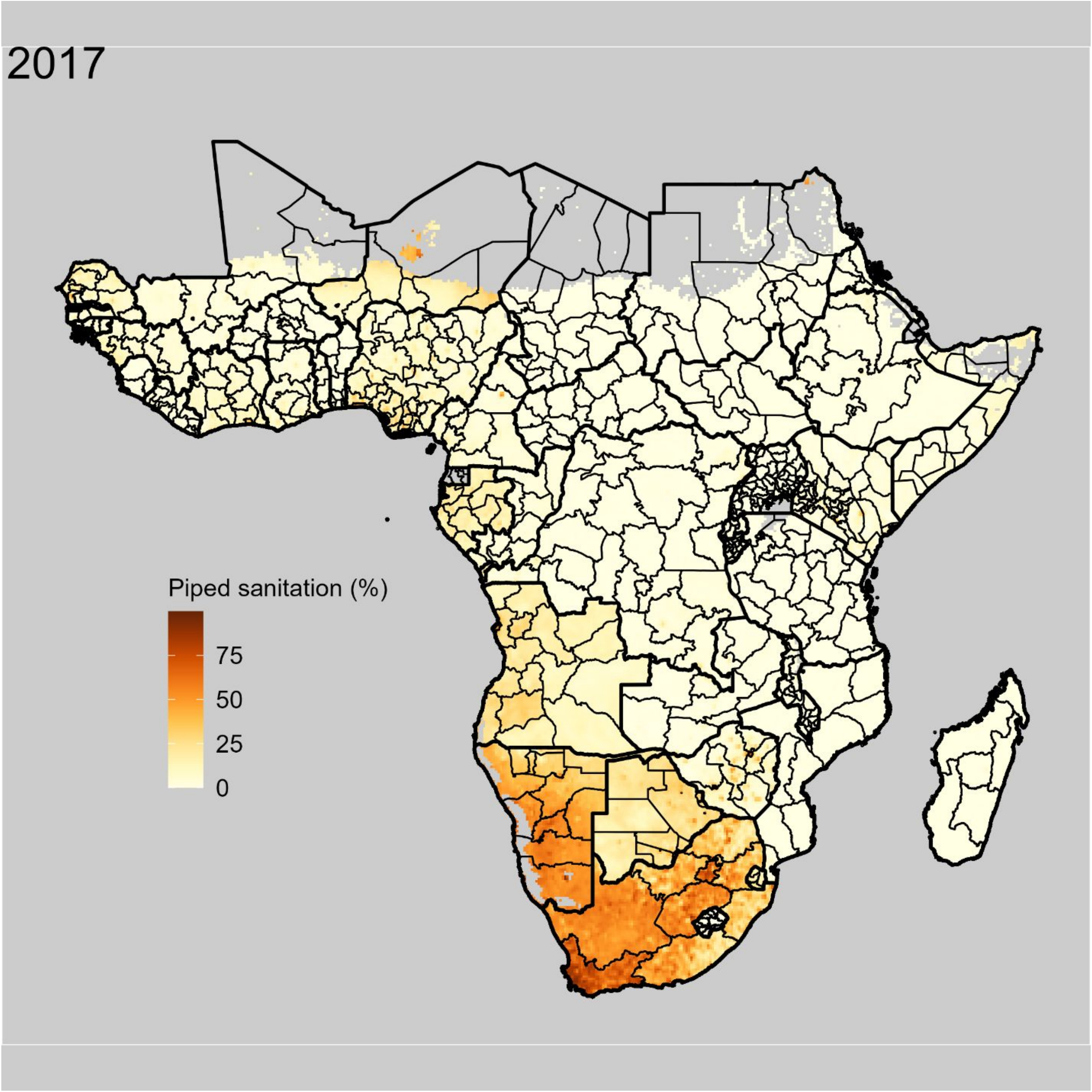

Figure S6. Travel time to the nearest healthcare facility. The values are based on a study that produce maps of travel time without access to motorized transport at 1 km by 1 km grids [5].

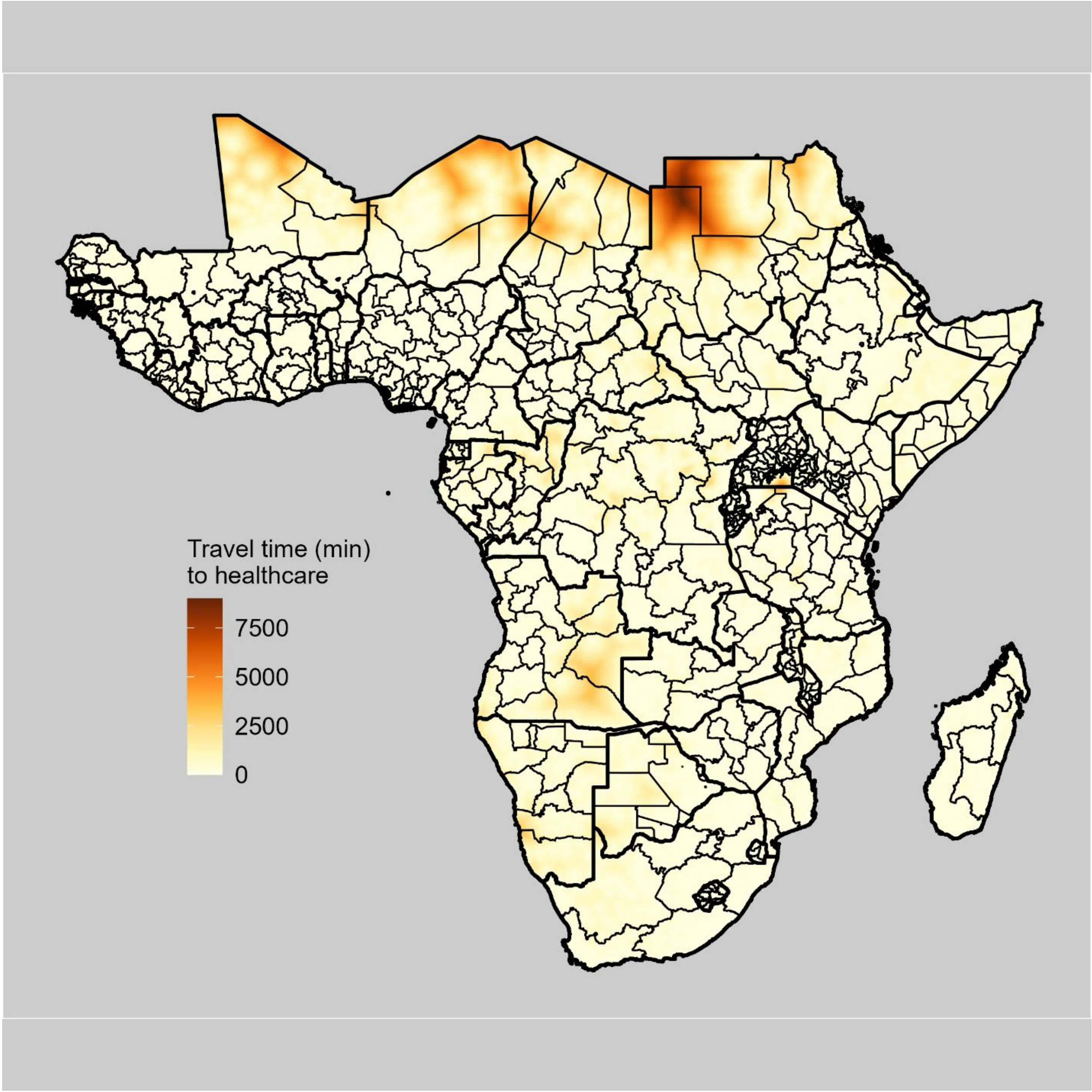

Table S1. Correlation among covariates. Pearson's *r* was computed using a data set of distance decay constant *k*=0.01. Highlighted cells indicate correlation coefficients among variables included in the final model.

	Pf incidence rate	Pf parasite rate	Improved water*	Open defecation	Piped sanitation*	Piped water	HIV*	Stunting	Surface water	Underweight*	Wasting
Pf incidence* rate	1	0.95	-0.35	-0.01	-0.46	-0.44	-0.15	0.3	0.04	0.08	-0.17
Pf parasite rate	0.95	1	-0.31	-0.05	-0.38	-0.36	-0.16	0.25	0.03	0.07	-0.13
Improved water*	-0.35	-0.31	1	-0.14	0.51	0.68	0.37	-0.45	-0.56	-0.39	-0.09
Open defecation	-0.01	-0.05	-0.14	1	-0.21	-0.09	-0.09	0.13	0.16	0.34	0.32
Piped sanitation*	-0.46	-0.38	0.51	-0.21	1	0.68	0.24	-0.29	-0.14	-0.28	-0.1
Piped water	-0.44	-0.36	0.68	-0.09	0.68	1	0.39	-0.35	-0.28	-0.35	-0.13
HIV*	-0.15	-0.16	0.37	-0.09	0.24	0.39	1	-0.17	-0.14	-0.57	-0.47
Stunting	0.3	0.25	-0.45	0.13	-0.29	-0.35	-0.17	1	0.34	0.58	0.08
Surface water	0.04	0.03	-0.56	0.16	-0.14	-0.28	-0.14	0.34	1	0.14	-0.11
Underweight*	0.08	0.07	-0.39	0.34	-0.28	-0.35	-0.57	0.58	0.14	1	0.76
Wasting	-0.17	-0.13	-0.09	0.32	-0.1	-0.13	-0.47	0.08	-0.11	0.76	1

* Covariates included in the final model

For peer review only

Figure S7. Receiver operating curve (ROC) from boosted regression tree model fits. The solid line represents the mean and dashed lines represent lower and upper bounds of the 95% confidence intervals. Area Under the ROC Curve (AUC) is 0.913 [95% CI: 0.911 to 0.915].

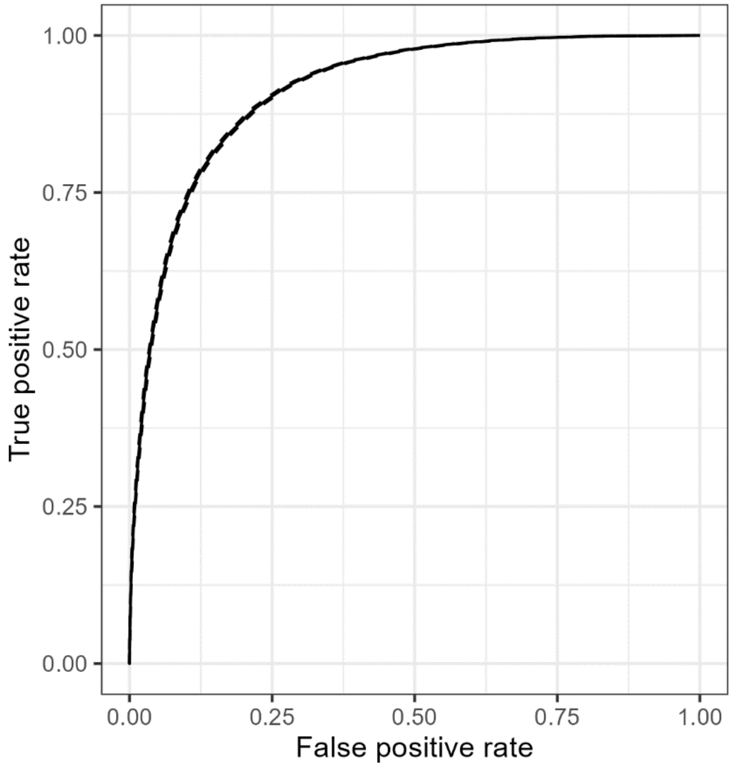

Table S2. Probability of iNTS occurrence for 2017 aggregated at the country level. Mean and the 95% confidence interval of the predicted probability from 400 simulation runs.

Country	Probability of occurrence
Angola	0.14 (95% CI: 0.07 to 0.25)
Burundi	0.32 (95% CI: 0.17 to 0.52)
Benin	0.25 (95% CI: 0.11 to 0.48)
Burkina Faso	0.29 (95% CI: 0.14 to 0.53)
Botswana	0.07 (95% CI: 0.03 to 0.18)
Central African Republic	0.47 (95% CI: 0.30 to 0.64)
Cote d'Ivoire	0.40 (95% CI: 0.24 to 0.61)
Cameroon	0.33 (95% CI: 0.19 to 0.50)
Congo, Democratic Republic of the	0.46 (95% CI: 0.26 to 0.68)
Congo, Republic of the	0.60 (95% CI: 0.40 to 0.78)

Djibouti	0.07 (95% CI: 0.03 to 0.13)
Eritrea	0.01 (95% CI: 0.01 to 0.03)
Ethiopia	0.06 (95% CI: 0.03 to 0.11)
Gabon	0.13 (95% CI: 0.03 to 0.41)
Ghana	0.66 (95% CI: 0.37 to 0.87)
Guinea	0.66 (95% CI: 0.41 to 0.86)
Gambia	0.42 (95% CI: 0.21 to 0.64)
Guinea-Bissau	0.59 (95% CI: 0.39 to 0.79)
Equatorial Guinea	0.02 (95% CI: 0.00 to 0.14)
Kenya	0.34 (95% CI: 0.19 to 0.51)
Liberia	0.48 (95% CI: 0.22 to 0.77)
Lesotho	0.25 (95% CI: 0.10 to 0.48)
Madagascar	0.03 (95% CI: 0.02 to 0.07)
Mali	0.38 (95% CI: 0.24 to 0.55)
Mozambique	0.58 (95% CI: 0.34 to 0.78)
Malawi	0.65 (95% CI: 0.49 to 0.81)
Namibia	0.10 (95% CI: 0.04 to 0.20)
Niger	0.02 (95% CI: 0.01 to 0.04)
Nigeria	0.21 (95% CI: 0.11 to 0.36)
Rwanda	0.38 (95% CI: 0.18 to 0.68)
Sudan	0.01 (95% CI: 0.00 to 0.01)
Senegal	0.30 (95% CI: 0.12 to 0.52)
Sierra Leone	0.23 (95% CI: 0.10 to 0.45)
Somalia	0.03 (95% CI: 0.01 to 0.07)
South Sudan	0.13 (95% CI: 0.07 to 0.25)
Swaziland	0.24 (95% CI: 0.08 to 0.74)
Chad	0.11 (95% CI: 0.06 to 0.19)
Togo	0.65 (95% CI: 0.42 to 0.82)
Tanzania, United Republic of	0.15 (95% CI: 0.06 to 0.33)
Uganda	0.23 (95% CI: 0.09 to 0.47)
South Africa	0.07 (95% CI: 0.03 to 0.18)
Zambia	0.49 (95% CI: 0.29 to 0.69)
Zimbabwe	0.40 (95% CI: 0.20 to 0.63)

For peer review only

Figure S8. Probability of iNTS occurrence from 2000 to 2020 every two years. Estimates represent the mean from 400 simulation runs. For animation, see https://github.com/kimfinale/iNTS_Mapping/tree/master/images.

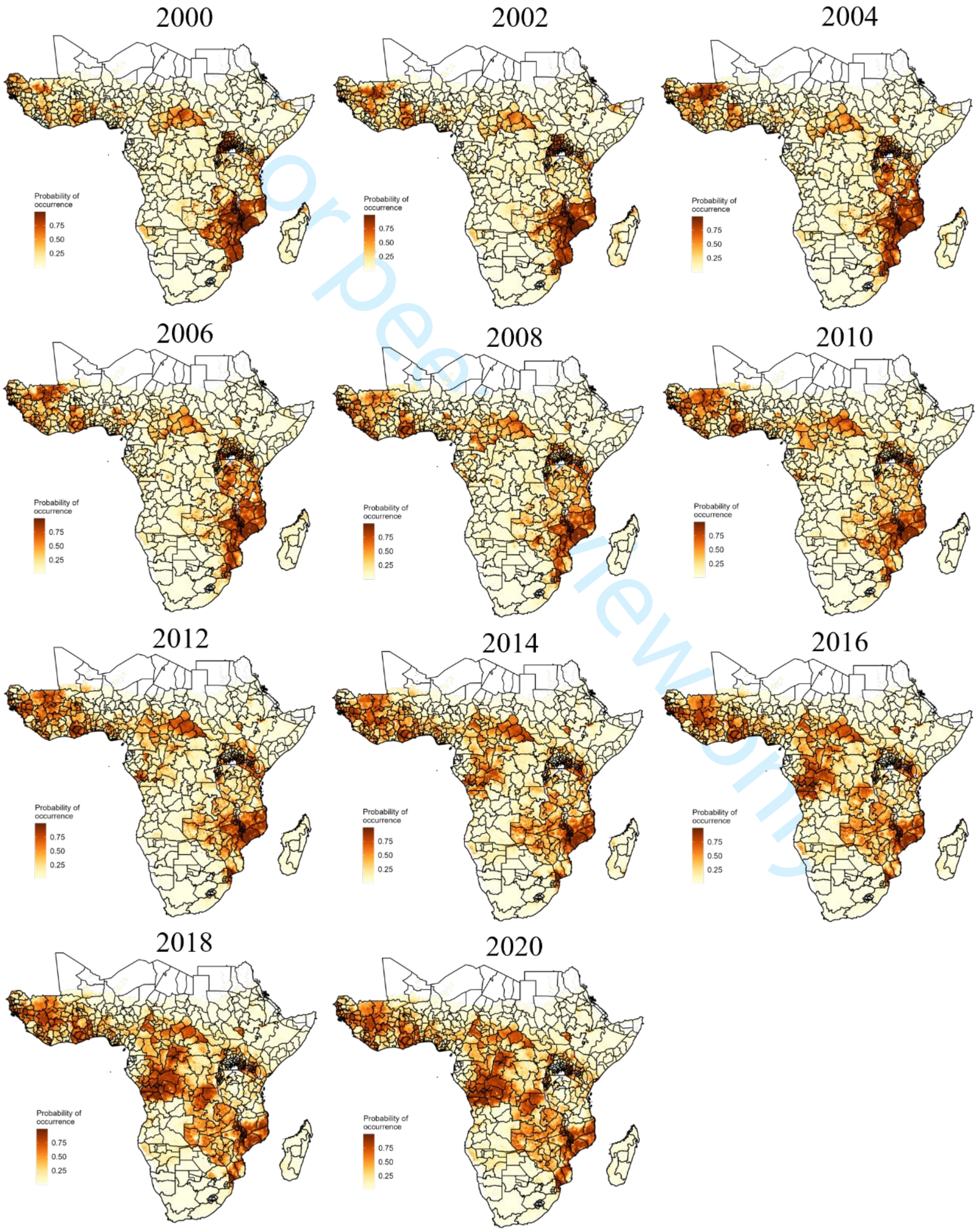

For peer review only

Table S3. Probability of iNTS occurrence aggregated at the regional level over 2000 - 2020. Values represent means from 400 simulation runs.

	Eastern Africa	Middle Africa	Southern Africa	Western Africa
2000	0.27	0.11	0.05	0.19
2001	0.28	0.11	0.06	0.19
2002	0.26	0.11	0.05	0.22
2003	0.27	0.11	0.05	0.24
2004	0.29	0.11	0.06	0.25
2005	0.28	0.12	0.05	0.25
2006	0.27	0.12	0.05	0.27
2007	0.27	0.14	0.05	0.27
2008	0.25	0.14	0.05	0.26
2009	0.25	0.16	0.04	0.28
2010	0.26	0.15	0.04	0.29
2011	0.25	0.16	0.04	0.30
2012	0.26	0.18	0.04	0.32
2013	0.26	0.20	0.04	0.32
2014	0.26	0.22	0.06	0.32
2015	0.25	0.25	0.04	0.33
2016	0.25	0.29	0.05	0.33
2017	0.24	0.34	0.08	0.33
2018	0.24	0.35	0.06	0.33
2019	0.24	0.35	0.05	0.33
2020	0.24	0.35	0.06	0.33

For peer review only

Table S4. Probability of iNTS occurrence aggregated at the country level over 2000 - 2020. Values represent means from 400 simulation runs.

Country	2000	2001	2002	2003	2004	2005	2006	2007	2008	2009	2010	2011	2012	2013	2014	2015	2016	2017	2018	2019	2020
Angola	0.05	0.05	0.05	0.05	0.05	0.05	0.05	0.05	0.05	0.05	0.05	0.05	0.06	0.08	0.09	0.11	0.13	0.14	0.14	0.14	0.14
Burundi	0.30	0.29	0.27	0.24	0.17	0.17	0.19	0.23	0.29	0.33	0.32	0.31	0.29	0.26	0.27	0.27	0.30	0.32	0.35	0.36	0.37
Benin	0.28	0.29	0.30	0.27	0.20	0.19	0.22	0.19	0.19	0.19	0.16	0.19	0.22	0.24	0.24	0.26	0.26	0.25	0.24	0.25	0.25
Burkina Faso	0.08	0.06	0.05	0.05	0.06	0.06	0.06	0.08	0.12	0.15	0.21	0.26	0.31	0.30	0.28	0.24	0.22	0.29	0.29	0.29	0.30
Botswana	0.02	0.02	0.01	0.01	0.01	0.01	0.01	0.01	0.02	0.03	0.04	0.02	0.02	0.02	0.04	0.02	0.04	0.07	0.04	0.03	0.04
CAR	0.45	0.42	0.41	0.42	0.42	0.42	0.44	0.45	0.46	0.50	0.48	0.46	0.55	0.53	0.52	0.50	0.51	0.47	0.46	0.46	0.46
Cote d'Ivoire	0.26	0.25	0.24	0.23	0.24	0.28	0.29	0.26	0.22	0.21	0.23	0.26	0.34	0.44	0.47	0.46	0.45	0.40	0.40	0.39	0.39
Cameroon	0.05	0.05	0.06	0.06	0.08	0.09	0.09	0.16	0.25	0.34	0.29	0.29	0.26	0.27	0.28	0.30	0.32	0.33	0.33	0.34	0.33
DR Congo,	0.09	0.09	0.09	0.10	0.10	0.10	0.10	0.10	0.10	0.11	0.11	0.13	0.15	0.17	0.20	0.25	0.34	0.46	0.48	0.48	0.48
Congo	0.05	0.05	0.05	0.07	0.07	0.08	0.09	0.10	0.12	0.15	0.19	0.23	0.28	0.38	0.52	0.63	0.65	0.60	0.59	0.59	0.59
Djibouti	0.02	0.02	0.03	0.04	0.03	0.05	0.05	0.04	0.03	0.04	0.03	0.03	0.03	0.03	0.04	0.05	0.06	0.07	0.08	0.11	0.12
Eritrea	0.04	0.05	0.04	0.04	0.02	0.02	0.02	0.02	0.01	0.01	0.01	0.01	0.01	0.01	0.01	0.01	0.01	0.01	0.01	0.02	0.02
Ethiopia	0.02	0.02	0.03	0.04	0.04	0.03	0.03	0.03	0.03	0.04	0.05	0.05	0.07	0.08	0.06	0.06	0.06	0.06	0.05	0.05	0.06
Gabon	0.03	0.04	0.06	0.04	0.07	0.08	0.12	0.21	0.17	0.14	0.12	0.11	0.13	0.11	0.11	0.12	0.12	0.13	0.14	0.14	0.13
Ghana	0.43	0.43	0.57	0.55	0.51	0.52	0.58	0.64	0.66	0.66	0.64	0.61	0.61	0.60	0.60	0.62	0.67	0.66	0.64	0.64	0.66
Guinea	0.27	0.33	0.44	0.48	0.42	0.38	0.40	0.45	0.50	0.55	0.56	0.56	0.61	0.64	0.66	0.66	0.66	0.66	0.67	0.68	0.68
Gambia	0.35	0.43	0.51	0.54	0.50	0.48	0.50	0.48	0.48	0.43	0.43	0.46	0.51	0.54	0.53	0.54	0.54	0.42	0.43	0.41	0.43
Guinea-Bissau	0.45	0.41	0.38	0.40	0.46	0.48	0.50	0.52	0.52	0.52	0.52	0.52	0.54	0.54	0.56	0.57	0.59	0.59	0.59	0.57	0.55
Equatorial Guinea	0.20	0.20	0.22	0.17	0.11	0.05	0.03	0.03	0.03	0.04	0.05	0.04	0.03	0.02	0.01	0.01	0.01	0.02	0.02	0.02	0.02
Kenya	0.19	0.20	0.20	0.20	0.19	0.20	0.20	0.22	0.27	0.27	0.30	0.31	0.36	0.34	0.32	0.32	0.33	0.34	0.32	0.30	0.30
Liberia	0.07	0.08	0.10	0.14	0.22	0.34	0.45	0.53	0.57	0.53	0.49	0.49	0.50	0.50	0.49	0.46	0.43	0.48	0.54	0.57	0.59
Lesotho	0.03	0.03	0.04	0.03	0.03	0.04	0.04	0.05	0.06	0.08	0.10	0.12	0.12	0.14	0.18	0.19	0.22	0.25	0.25	0.25	0.25
Madagascar	0.07	0.13	0.11	0.11	0.08	0.09	0.07	0.04	0.03	0.02	0.02	0.02	0.03	0.04	0.05	0.04	0.03	0.03	0.03	0.03	0.03
Mali	0.18	0.24	0.32	0.42	0.46	0.41	0.45	0.42	0.34	0.44	0.47	0.47	0.46	0.46	0.43	0.41	0.41	0.38	0.36	0.36	0.39
Mozambique	0.77	0.80	0.81	0.80	0.79	0.77	0.75	0.73	0.70	0.68	0.67	0.68	0.65	0.63	0.60	0.58	0.59	0.58	0.59	0.59	0.60
Malawi	0.88	0.88	0.87	0.84	0.82	0.80	0.76	0.70	0.65	0.62	0.60	0.64	0.64	0.63	0.64	0.63	0.64	0.65	0.64	0.64	0.66

For peer review only

Namibia	0.08	0.08	0.06	0.05	0.05	0.04	0.05	0.04	0.03	0.02	0.02	0.02	0.02	0.03	0.08	0.07	0.10	0.10	0.10	0.11	0.09
Niger	0.02	0.02	0.02	0.02	0.02	0.02	0.02	0.02	0.02	0.02	0.02	0.02	0.02	0.01	0.01	0.02	0.02	0.02	0.02	0.02	0.02
Nigeria	0.11	0.11	0.12	0.11	0.13	0.14	0.14	0.15	0.14	0.14	0.14	0.15	0.17	0.16	0.18	0.20	0.21	0.21	0.21	0.21	0.21
Rwanda	0.31	0.34	0.37	0.40	0.43	0.43	0.39	0.36	0.30	0.25	0.22	0.25	0.28	0.33	0.45	0.49	0.41	0.38	0.38	0.41	0.59
Sudan	0.00	0.00	0.00	0.00	0.00	0.00	0.00	0.00	0.00	0.00	0.00	0.00	0.00	0.01	0.01	0.01	0.01	0.01	0.01	0.01	0.01
Senegal	0.44	0.26	0.27	0.35	0.31	0.31	0.36	0.35	0.34	0.29	0.35	0.32	0.34	0.31	0.26	0.31	0.27	0.30	0.35	0.29	0.36
Sierra Leone	0.24	0.21	0.17	0.30	0.42	0.47	0.45	0.46	0.39	0.30	0.30	0.23	0.24	0.27	0.29	0.28	0.25	0.23	0.23	0.23	0.22
Somalia	0.12	0.10	0.09	0.09	0.09	0.08	0.07	0.05	0.04	0.03	0.03	0.02	0.02	0.02	0.02	0.02	0.02	0.03	0.03	0.04	0.03
South Sudan	0.07	0.07	0.08	0.07	0.07	0.07	0.08	0.08	0.09	0.11	0.12	0.12	0.12	0.12	0.13	0.13	0.13	0.13	0.13	0.14	0.14
Swaziland	0.53	0.59	0.63	0.66	0.70	0.66	0.64	0.61	0.57	0.54	0.49	0.45	0.42	0.41	0.36	0.31	0.26	0.24	0.25	0.23	0.23
Chad	0.06	0.06	0.05	0.04	0.04	0.04	0.08	0.10	0.09	0.08	0.08	0.08	0.09	0.10	0.11	0.10	0.10	0.11	0.11	0.11	0.11
Togo	0.14	0.17	0.17	0.18	0.19	0.20	0.22	0.23	0.26	0.30	0.37	0.39	0.44	0.45	0.53	0.56	0.60	0.65	0.65	0.65	0.65
Tanzania	0.22	0.21	0.19	0.33	0.51	0.53	0.48	0.40	0.36	0.32	0.30	0.28	0.26	0.28	0.28	0.25	0.20	0.15	0.15	0.15	0.15
Uganda	0.65	0.65	0.63	0.60	0.59	0.56	0.51	0.47	0.46	0.45	0.43	0.41	0.36	0.31	0.27	0.24	0.23	0.23	0.22	0.21	0.23
South Africa	0.03	0.06	0.06	0.07	0.07	0.06	0.06	0.06	0.05	0.05	0.05	0.05	0.05	0.05	0.05	0.03	0.03	0.07	0.05	0.03	0.03
Zambia	0.32	0.33	0.29	0.27	0.25	0.25	0.25	0.26	0.28	0.30	0.32	0.35	0.39	0.43	0.45	0.47	0.49	0.49	0.49	0.48	0.48
Zimbabwe	0.61	0.52	0.44	0.37	0.34	0.29	0.30	0.48	0.36	0.39	0.46	0.33	0.37	0.37	0.38	0.38	0.37	0.40	0.41	0.40	0.37

For peer review only

Figure S9. Probability of iNTS occurrence for 2000, 2010, and 2017 for k=0.01, k=0.02, k=0.005. Average radius for the catchment area for the secondary and tertiary hospital is 100 km (baseline), 50 km, and 200 km, respectively for k=0.01, k=0.02, and k=0.005.

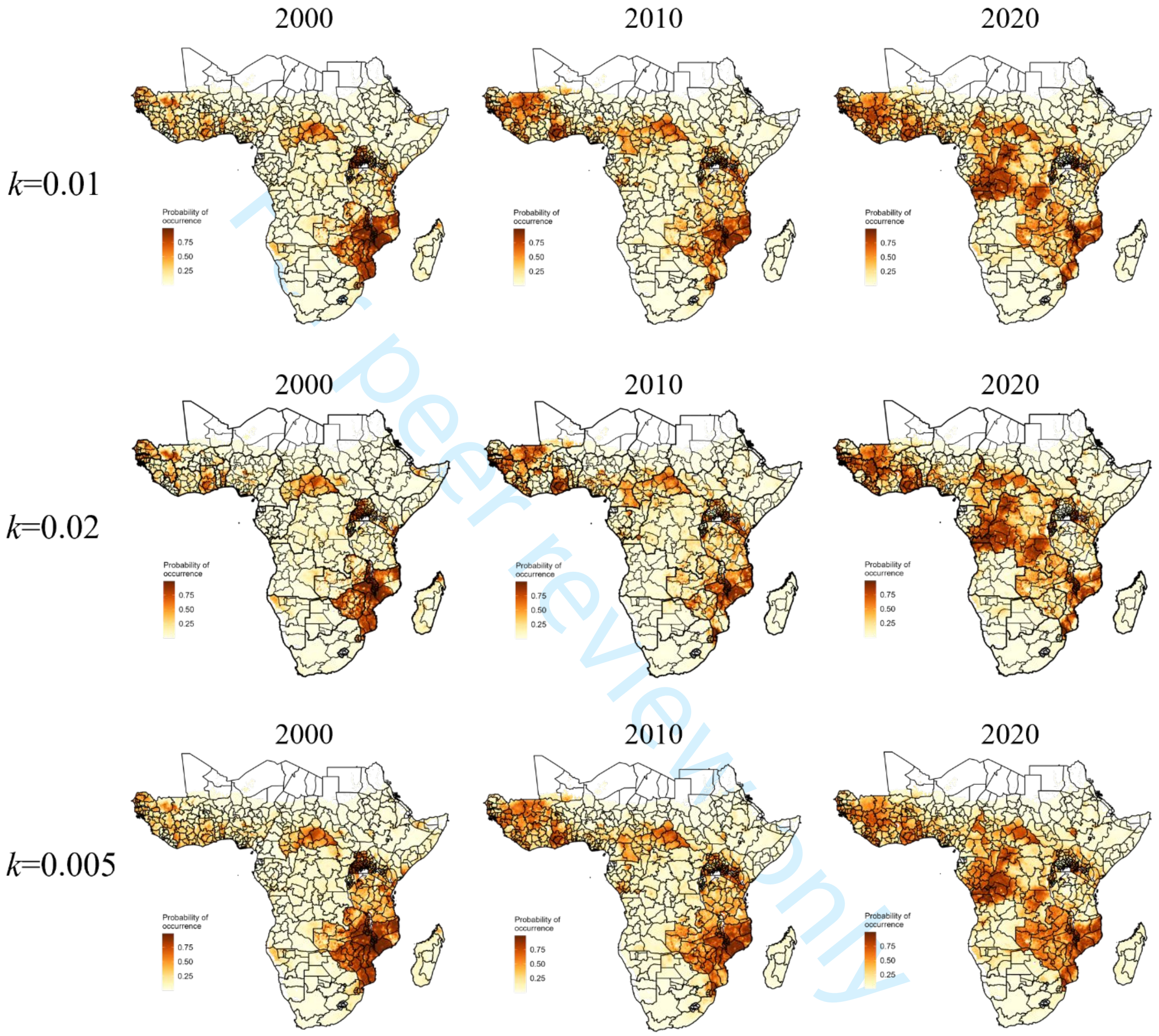

For peer review only

BMJ Open

Examining geospatial and temporal distribution of invasive non-typhoidal Salmonella disease occurrence in sub-Saharan Africa: A systematic review and modeling study

Journal:	*BMJ Open*
Manuscript ID	bmjopen-2023-080501.R1
Article Type:	Original research
Date Submitted by the Author:	02-Feb-2024
Complete List of Authors:	Kim, Jong-Hoon; International Vaccine Institute,<br>Tack, Bieke ; Institute of Tropical Medicine, Clinical Sciences<br>Fiorino, Fabio; University of Siena<br>Pettini, Elena; University of Siena<br>Marchello, Christian; New Zealand Ministry of Health<br>Jacobs, Jan; Institute of Tropical Medicine, Department of Clinical Sciences<br>Crump, John; University of Otago, Centre for International Health<br>Marks, Florian ; International Vaccine Institute, Epidemiology Unit; University of Cambridge, Department of Medicine<br>Vacc-iNTS Consortium, Collaborators; Vacc-iNTS Consortium,
**Primary Subject Heading**:	Epidemiology
Secondary Subject Heading:	Global health, Public health
Keywords:	Epidemiology < TROPICAL MEDICINE, Epidemiology < INFECTIOUS DISEASES, Risk Factors, PUBLIC HEALTH

SCHOLARONE™
Manuscripts

I, the Submitting Author has the right to grant and does grant on behalf of all authors of the Work (as defined in the below author licence), an exclusive licence and/or a non-exclusive licence for contributions from authors who are: i) UK Crown employees; ii) where BMJ has agreed a CC-BY licence shall apply, and/or iii) in accordance with the terms applicable for US Federal Government officers or employees acting as part of their official duties; on a worldwide, perpetual, irrevocable, royalty-free basis to BMJ Publishing Group Ltd ("BMJ") its licensees and where the relevant Journal is co-owned by BMJ to the co-owners of the Journal, to publish the Work in this journal and any other BMJ products and to exploit all rights, as set out in our licence.

The Submitting Author accepts and understands that any supply made under these terms is made by BMJ to the Submitting Author unless you are acting as an employee on behalf of your employer or a postgraduate student of an affiliated institution which is paying any applicable article publishing charge ("APC") for Open Access articles. Where the Submitting Author wishes to make the Work available on an Open Access basis (and intends to pay the relevant APC), the terms of reuse of such Open Access shall be governed by a Creative Commons licence – details of these licences and which Creative Commons licence will apply to this Work are set out in our licence referred to above.

Other than as permitted in any relevant BMJ Author's Self Archiving Policies, I confirm this Work has not been accepted for publication elsewhere, is not being considered for publication elsewhere and does not duplicate material already published. I confirm all authors consent to publication of this Work and authorise the granting of this licence.

For peer review only

Title

Examining geospatial and temporal distribution of invasive non-typhoidal *Salmonella* disease occurrence in sub-Saharan Africa: A systematic review and modeling study

Authors

Jong-Hoon Kim*, Bieke Tack, Fabio Fiorino, Elena Pettini, Christian S. Marchello, Jan Jacobs, John A. Crump, Florian Marks, on behalf of the Vacc-iNTS consortium collaborators*

*Corresponding author

Email: jonghoon.kim@ivi.int

Postal address: SNU Research Park, 1 Gwanak-ro, Gwanak-gu, Seoul 08826, Republic of Korea

Word count: 3,954

For peer review only

Abstract

Background: Invasive non-typhoidal *Salmonella* (iNTS) disease is a significant health concern in sub-Saharan Africa. While our knowledge of a larger-scale variation is growing, understanding of the subnational variation in iNTS disease occurrence is lacking, yet crucial for targeted intervention.

Method: We performed a systematic review of reported occurrences of iNTS disease in sub-Saharan Africa, consulting literature from PubMed, Embase, and Web of Science published since 2000. Eligibility for inclusion was not limited by study type but required that studies reported original data on human iNTS diseases based on culture of a normally sterile site, specifying subnational locations and the year, and were available as full-text articles. We excluded studies that diagnosed iNTS disease based on clinical indications, cultures from non-sterile sites, or serological testing. We estimated the probability of occurrence of iNTS disease for sub-Saharan Africa on 20 km × 20 km grids by exploring the association with geospatial covariates such as malaria, HIV, childhood growth failure, access to improved water, and sanitation using a boosted regression tree.

Results: We identified 130 unique references reporting human iNTS disease in 21 countries published from 2000 through 2020. Estimated probability of iNTS occurrence grids showed significant spatial heterogeneity at all levels (20 km by 20 km grids, subnational, country, and subregional levels) and temporal heterogeneity by year. For 2020, the probability of occurrence was higher in Middle Africa (0.34, 95% CI: 0.25 to 0.46), followed by Western Africa (0.33, 95% CI: 0.23 to 0.44), Eastern Africa (0.24, 95% CI: 0.17 to 0.33), and Southern Africa (0.08, 95% CI: 0.03 to 0.11). Temporal heterogeneity indicated that the probability of occurrence increased between 2000 and 2020 in countries such as Republic of the Congo (0.05 to 0.59) and Democratic Republic of the Congo (0.10 to 0.48) whereas it decreased in countries such as Uganda (0.65 to 0.23) or Zimbabwe (0.61 to 0.37).

Conclusion: The iNTS disease occurrence varied greatly across sub-Saharan Africa, with certain regions being disproportionately affected. Exploring regions at high risk for iNTS disease, despite the limitations

For peer review only

in our data, may inform focused resource allocation. This targeted approach may enhance efforts to combat iNTS disease in more affected areas.

Strengths and limitations of this study:

- We examined geospatial and temporal distribution of the probability of occurrence of invasive non-typhoidal *Salmonella* (iNTS) disease for sub-Saharan Africa on 20 km by 20 km grids.
- Occurrence data points are few with cumulative occurrences from 2000 through 2020 only representing around 3% of the 20 km by 20 km grids of sub-Saharan Africa.
- iNTS disease represents only a fraction of transmission of the pathogen and thus it is likely that the estimates of the occurrence of iNTS disease underestimate the transmission of the pathogen.

Keywords: Epidemiology; *Salmonella* infections; *Salmonella* Typhimurium; *Salmonella* Enteritidis; Machine Learning; sub-Saharan Africa

Introduction

Non-typhoidal *Salmonella* (NTS) are a leading cause of invasive bacterial disease in sub-Saharan Africa. Invasive non-typhoidal *Salmonella* (iNTS) disease was estimated to have caused 535,000 cases and 77,500 deaths in 2017 [1]. More than three-quarters of these cases and deaths occurred in sub-Saharan Africa, where the incidence rates were estimated at 44.8 per 100,000 person years observations and case fatality was estimated at 17.1% [2,3].

The geographic predominance of iNTS disease in sub-Saharan Africa can be partially explained by the prevalence of key host risk factors and the transmission of human-adapted NTS in the region [4,5]. While

For peer review only

NTS strains circulating in other settings typically cause self-limiting foodborne diarrheal disease, genetic adaptations observed in the African strains resulted in increased invasive disease [6] and possibly the establishment of a human reservoir [7]. Impaired immunity due to young age, recent or current *Plasmodium falciparum* malaria infection, anemia, malnutrition, and HIV infection renders the sub-Saharan African population susceptible to iNTS disease, in particular among infants and children under the age of five years [4]. In addition to these host risk factors, environmental risk factors for iNTS disease such as unimproved water sources contribute to the high incidence of iNTS disease in sub-Saharan Africa [7].

While existing studies provide estimates at the country, regional, and global levels mainly based on the incidence rates from population-based surveillance studies [1,2,8], they fail to provide geospatial heterogeneity of iNTS disease at the subnational level, which is crucial for prioritizing areas for targeted iNTS surveillance and control programs. We aimed to characterize the geospatial variation in probability of iNTS occurrence across sub-Saharan Africa (sSA) at a resolution of 20 km × 20 km using iNTS disease occurrences reported in the literature and geospatial data of host and environmental iNTS risk factors.

For peer review only

Methods

<u>iNTS occurrence data</u>

We sought data on iNTS occurrence and incidence rates in the peer-reviewed literature available in PubMed, Embase and Web of Science published from January 1, 2000 through June 30, 2021 in the English language. We designed the search strings to account for each database and varying usage of the terms, non-typhoidal *Salmonella*, and to restrict the topic to human infections in sub-Saharan Africa while adapting to the syntax of different databases (Supplementary Material S1). We conducted the review according to the Preferred Reporting Items for Systematic Reviews and Meta Analyses (PRISMA) [9].

We used Rayyan [10] for a collaborative review of the literature and collated the data in Google sheet. The PRISMA flowchart shows an overview of the literature review process (**Figure 1**).

Three authors (BT, FF, and EP) reviewed the literature and collated the data with at least two independent reviewers reviewing each article. Disagreements between the reviewers were resolved after discussion with the reviewers and the first author (JHK). Studies were eligible regardless of the study type (e.g., outbreak investigation, population-based surveillance, or case report) if they reported original findings of human iNTS disease defined as isolation of non-typhoidal *Salmonella* from a normally sterile site (e.g., blood, cerebrospinal fluid, bone marrow) in a patient with subnational location and year as a full text article. Conference proceedings were excluded. We excluded studies that used clinical indication (i.e., symptoms and signs), culture of a non-sterile site (e.g., stool), or serology to classify a iNTS disease. We excluded studies that did not report iNTS separately from *Salmonella* Typhi or *Salmonella* Paratyphi A, B, or C. Abstracted variables include year, location, diagnostic method, and the number of iNTS disease cases. If observations spanned multiple years, the year of occurrence for each iNTS disease case was selected uniformly at random among observation years, which leads to the same number of cases for each year on average.

Geo-positioning of iNTS occurrence

iNTS occurrence was defined as a 20 km × 20 km grid cell for which the spatial resolution was chosen by taking into account the feasibility to explore subnational heterogeneity, computational cost, and a previous study of the similar resolution [11]. The position was defined as a grid cell to which longitude and latitude of the area belongs based on the Google Maps (Alphabet Inc., Mountain View, California, US). If the record reported the subnational area in which iNTS was reported was smaller than a grid cell, we assumed that the iNTS disease occurred in the grid cell. On the other hand, if the subnational administrative units included more than one grid cell, we assumed that iNTS disease could occur on any of the grid cells within the boundary and used the covariate value averaged over the grid cells that fall on the area. If the occurrence was reported in a hospital, the grid cell for occurrence was selected randomly

For peer review only

assuming that the probability that the iNTS disease appeared at d km from the hospital is de^{-kd}, where the rate of exponential decay k was chosen differentially according to the catchment area of the hospital. The exponential decay of the probability of was based on the idea that the frequency of visits to a hospital decreases with the distance, more generally known as distance decay, from the hospital following an exponential decay [12]. We assumed that the catchment area was 20 km and 100 km for primary and secondary or tertiary hospitals, respectively (i.e., k was 0.05 and 0.01 for primary and secondary/tertiary hospitals, respectively), which implies that 63% of the cases occurred within the boundary of 20 km and 100 km, respectively, and around 86% of the cases occurred within the boundary of 40 km and 200 km, respectively. We conducted a sensitivity analysis by assuming average distance of 50 km and 200 km for the iNTS occurrence location from the tertiary hospital (i.e., 0.02 or 0.005 for k), respectively.

<u>Geospatial covariates</u>

Our study investigated factors that may affect the risk of iNTS disease across sub-Saharan Africa, focusing on a variety of host [13] and environmental factors [7]. We examined these factors at a detailed resolution of at least 20 km × 20 km across the continent. For host factors, we looked into the prevalence of HIV (as shown in **Figure S1**) [14], *Plasmodium falciparum* infection (**Figure S2**) [15], and child growth failure, including underweight conditions (**Figure S3**) [16]. An association between these factors and iNTS disease has been observed in multiple sub-Saharan African countries. For example, iNTS disease was more common in children with HIV in Kenya [17] and adults in Malawi [18] compared with HIV-uninfected persons. Similarly, children suffering from malnutrition in countries like Kenya [17], Mozambique [19,20], Ghana [21], and Tanzania [22] showed higher instances of iNTS disease compared to children without malnutrition. An association between iNTS disease and malaria has been widely reported, including in studies from Malawi [23] and Tanzania [22]. We also explored the impact of environmental factors, access to improved water sources (**Figure S4**) and sanitation facilities (**Figure S5**) [24], on iNTS disease This set of covariates was similar to that used in modeling the global burden of iNTS disease [1].

For peer review only

The HIV prevalence represented estimates of HIV prevalence among adults aged 15-45 years in sub-Saharan Africa, annually from 2000 through 2017, based on the review of various surveys including AIDS Indicator Survey, Demographic and Health Survey, Multiple Indicator Cluster Survey, Population-based HIV Impact Assessment Survey, and sentinel surveillance of women attending antenatal care clinics. The mean estimates were downloaded from the website for the Global Health Data Exchange (GHDx) [25]. The *P falciparum* parasite rate represents the proportion of children aged 2-10 years showing detectable *P. falciparum* parasite on a given year from 2000 through 2020 and estimated based on national surveys such as the Demographic and Health Survey data and the literature. The mean estimates were downloaded from the website for the Malaria Atlas Project [26]. Childhood growth failure measured the proportion of children with height-for-age, weight-for-height, and weight-for-age (expressed as stunting, wasting, and underweight, respectively) *z* score that was more than two standard deviations below the World Health Organization's median growth reference standards for a healthy population. The mean estimates for 2000-2017 were downloaded from the GHDx [27]. Access to improved drinking water source and access to improved sanitation facilities represent the proportion of population who have access to alternative levels of water and sanitation (e.g., access to piped water or access to improved water) from 2000 to 2017. The mean estimates were downloaded from GHDx [28]. All covariates were available at the resolution of 5 km by 5 km grids and were aggregated to 20 km by 20 km grids by computing the mean.

We matched the year of data collection and the year for geospatial covariates as closely as possible. All covariates were available for each year from 2000 to 2017 and were matched to occurrences observed in the same year. For occurrences observed before 2000, we used the covariates for the year 2000. For occurrences observed after 2017, we used the covariates for 2017 for water, sanitation, and childhood growth failure, as these covariates were only available up to 2017. However, we were able to use the covariates for Malaria and HIV through 2020, so we matched the years between the occurrences and

For peer review only

covariates for those covariates. Additionally, we matched the location of the data collection and the covariates at 20-km by 20-km grids.

Probability of occurrence

In this analysis, the outcome is the probability of iNTS occurrence estimated on 20-km by 20-km grids, with each grid serving as the unit of analysis. This approach parallels the classic logistic regression, where the log odds of an event's probability (in this case, probability of iNTS occurrence) within a grid is modeled based on a set of potential risk factors (i.e., predictors). These predictors, which are geospatial covariates mentioned previously, were also structured on 20-km by 20-km grids. However, a key distinction arises in how these predictors are combined. Unlike logistic regression, which employs a linear combination of predictors, this model does not assume a linear relationship between covariates and outcome variables and can characterize complex interactions by utilizing the boosted regression tree boosted regression tree (BRT) model [29]. The BRT model integrates strengths of regression trees, where relationship between an outcome and predictors is modeled through recursive binary splits, and boosting, where many models are combined to improve predictive performance. The BRT framework could use background (or pseudo-absence) data as a substitute for true absence data. Background data [30] represented random grid cells and were sampled such that probability of being selected increased with decreasing travel time to the healthcare facilities based on the travel time to healthcare facilities [31] (**Figure S6**). This choice of background sampling strategy may mitigate a potential bias in our dataset that may overrepresent occurrences in areas near healthcare facilities. In the BRT, each regression tree includes only explanatory covariates that improve predictions when added over the existing regression trees.

For peer review only

We measured the relative importance of a covariate as proposed in the previous study [29] by measuring how often a variables is selected for splitting individuals trees, weighted by the squared improvement to the model as a result of each split, and averaged over all trees.

We grouped the covariates into five categories: HIV, malaria, child growth failure, water, and sanitation. Several variants (e.g., access to improved water source vs. access to piped water) were available for all categories except for HIV (**Table S1).** We chose one variable from each category that has the highest influence on the prediction of iNTS occurrence based on the relative importance of covariates from the model containing all covariates to improve interpretability considering that covariates in the same category often have high correlation (e.g., *Plasmodium falciparum* parasite prevalence and incidence rate has Pearson's r of 0.95, see **Table S1** for details).

<u>Model fitting and validation</u>

Key parameters in the BRT model include learning rate (LR), tree complexity (TC), and the number of trees. LR, also known as the shrinkage parameter, determines the contribution of each tree to the growing model. The TC controls whether interactions are fitted with a model with a TC of one fitting an additive model, and a TC of two fitting a model with up to two-way interactions, and so on [29]. We determined the values of these two parameters (i.e., LR of 0.05 and TC of 5) through a grid search in which the number of trees was optimized through 10-fold cross-validation for each set of TC and LR by taking the model performance and computation time into account. For example, we chose TC of 5 over TC of 10 by considering increasing the TC to 10 improved prediction by 2% but the computation time increased by a factor of two. In the 10-fold cross-validation the dataset was randomly divided into 10 subsets and models were trained using 9 subsets and were tested against the 10th subset. This process was repeated for each of the 10 subsets and the number of trees was optimized by minimizing average prediction deviance. The set of hyperparameters with optimized number of regression trees that produced the lowest deviance was selected as the final model. We identified the final model for each of 400 different data sets.

For peer review only

The predictive capacity of the final model was evaluated by calculating the receiver operating characteristic (ROC) curve and an area under the ROC curve (AUC) [32]. The models were implemented in statistical software R (version 4.1.3). Raster images were processed for modeling using raster package and the BRT model were implemented using gbm and dismo packages. All figures were prepared using ggplot2 package. All the codes and datasets are available on GitHub [33].

Summarizing results

Because of the random nature of the occurrence data (e.g., location for the occurrence reported in the hospital was randomly assigned by the decay function), we created 20 occurrence data sets. For each of the occurrence data sets, we created 20 different background data sets. Probability of occurrence (mean, 2.5th percentile, and 97.5th percentile) was predicted by summarizing these 400 BRT model simulations based on 400 different data set for every 20 km by 20 km grid cell. The probability of occurrence was aggregated to the administrative units (e.g., districts and provinces) as well as the 20 km by 20 km grid cells. The administrative unit areas were defined based on shapefiles from the Global Administrative Areas (GADM) website [34]. We calculated annual estimates of the probability of occurrence for each year from 2000 to 2017, utilizing the covariates specific to each year. Additionally, we produced forecasts for the years 2018 to 2020. During this period, the malaria parasite rate was the only covariate that varied annually. The other four covariates were kept constant, using their values as observed in 2017.

Patient and Public Involvement

This study does not involve any human participants.

Results

We identified 131 unique articles reporting cases of human iNTS disease. Years of publication ranged from 2000 through 2020 (**Figure 1**) while the study years ranged from 1979 through 2020. The number of reports were heterogeneous across year and country with overall 31,016 iNTS disease occurrences reported from 19 countries, and no report of iNTS disease occurrence in the remaining 26 countries of

For peer review only

sub-Saharan Africa, which does not necessarily mean the absence of occurrences (**Figure 2A**). Of iNTS disease occurrence reports, 25 (19%) were from Kenya, 25 (19%) from Malawi, 13 (10%) from Ghana, 10 (8%) from Tanzania, and 58 (44%) from other African countries. Overall, 42,699 iNTS disease cases for which year and location were clearly identified were reported from 2000 through 2020 with variation by year and country (**Figure 2B**). Malawi was the most substantial contributor (*n*=19,075, 62%) followed by Kenya (*n*=3539, 11%), Mali (*n*=2215, 7%), and Mozambique (*n*=1947, 6%). A majority of studies (*n*=123, 94%) included blood culture for confirmation of iNTS and the next most common diagnostic methods were culture of cerebrospinal fluid culture (*n*=14, 11%) followed by culture of pleural (*n*=2, 2%) or peritoneal (*n*=1, <1%) fluids. Around 67% (*n*=88) of the iNTS disease occurrence reports were from tertiary hospitals while about 13% (*n*=17) came from the primary hospitals and 20% (*n*=26) of the reports clearly indicated the number of administrative units from which the patients came. These reports led to an average of 5,504 iNTS disease occurrences on 20 km by 20 km grids, representing around 3% of 180,490 grid cells comprising sub-Saharan Africa. A majority of occurrence focused on the same set of countries (i.e., Malawi, Kenya, Mali, and Mozambique) that contributed most in terms of the number of cases (**Figure 3**).

<u>Probability of iNTS occurrence</u>

Of the 11 geospatial covariates that we explored, we selected five for the final model: *P falciparum* incidence rate, access to improved water, access to piped sanitation, prevalence of HIV, and prevalence of underweight (**Table S1**). The Pearson correlation coefficient (*r*) among those five covariates ranged from 0.08 to 0.57. The BRT model predicted well the occurrence of iNTS with the receiver operating characteristic (ROC) curve indicating an area under the ROC Curve (AUC) score of around 0.9 (**Figure S7**). Estimated probability of iNTS occurrence on 20 km by 20 km grids for 2017 showed significant spatial heterogeneity (**Figure 4A-4C**). The probability of occurrence of iNTS aggregated at the subregional level was highest in Middle Africa (0.34, 95% CI: 0.25 to 0.46) followed by Western Africa (0.33, 95% CI: 0.23 to 0.44), Eastern Africa (0.24, 95% CI: 0.17 to 0.33), and Southern Africa (0.08, 95%

For peer review only

263 CI: 0.03 to 0.11) while high-risk grid cells appear to be scattered across the continent. Aggregated at the
264 country level, probability of occurrence was highest at Malawi and lowest in Niger (**Table S2**).
265 Probability of occurrence averaged across administrative unit level 1 (i.e., province) showed similar
266 geospatial variations (**Figure 4D**).

267 Contribution of covariates

268 Geospatial covariates showed varying contributions with underweight having the highest contribution
269 (27.2% [95% CI: 26.2 to 28.1]) followed by HIV (23.8% [95% CI:23.1 to 24.5]), malaria (20.1% [95%
270 CI:18.4 to 21.6]), sanitation (18.7% [95% CI:17.6 to 20.3]), and drinking water (10.2% [95% CI: 9.6 to
271 11.0]) (**Figure 5A**). The relationship between the probability of iNTS disease occurrence and the
272 covariate proved to be complex (**Figure 5B**). The overall relationship between the probability of
273 occurrence and the risk factors such as *P falciparum* incidence rate, HIV prevalence, and underweight
274 prevalence appeared to have a positive relationship.

275 Temporal heterogeneity of the probability of occurrence

276 The spatial distribution of the probability of occurrence varied by year. For 2000, probability of
277 occurrence was predicted to be highest around Malawi with the country-aggregated value to be 0.88 [95%
278 CI: 0.77 to 0.94]. The next was Mozambique (0.77; 95% CI: 0.62 to 0.87) followed by Uganda (0.65;
279 95% CI: 0.43 to 0.82) and Zimbabwe (0.62; 95% CI: 0.35 to 0.83) (**Figure 6A** and **6D**). In 2010,
280 Mozambique (0.67; 95% CI: 0.47 to 0.83) had the highest probability of occurrence. Countries in Western
281 Africa such as Ghana (0.64; 95% CI: 0.43 to 0.81), Guinea (0.56; ; 95% CI: 0.34 to 0.75), Guinea-Bissau
282 (0.52; 95% CI: 0.33 to 0.71), and Liberia (0.49; 95% CI: 0.29 to 0.71) had high probability of occurrence,
283 with the largest increase between 2000 and 2010 observed in Liberia (0.07 vs. 0.49) (**Figure 6A, 6B,** and
284 **6D**). From 2000 through 2020, probability of occurrence aggregated at the country level increased most in
285 Republic of the Congo (0.05 vs. 0.59) while decreased most in Uganda (0.65 vs. 0.23) (**Figure 6C and**
286 **6D**).

For peer review only

Discussion

We examined geospatial and temporal distribution of the probability of occurrence iNTS for sub-Saharan Africa on 20 km by 20 km grids using the data collated from an extensive literature review and high-resolution geospatial covariates representing potential risk factors for iNTS disease. Estimated probability of iNTS disease occurrence showed substantial geospatial heterogeneity on 20 km by 20 km grid cells, subnational, and national levels and also temporal variation when measured by year. In 2017, the year for which most recent estimates for the modeled covariates were available, the estimated probability of occurrence was higher in Middle Africa and Western Africa and lower in Eastern Africa and Southern Africa.

Our study is the first to characterize the probability of iNTS occurrence at the sub-national levels. While predicted incidence rates at the national level are available [1], these predictions were based on a small number of surveillance studies. Having a means to identify sub-national high-risk areas using occurrence data as well as surveillance data is an important addition to the existing knowledge of iNTS epidemiology. The findings may be useful for designing effective and efficient intervention programs. Candidate vaccines for iNTS are under active development [35] and deployment strategies are critical for successful intervention and high return on investment. The current study may help identify areas of potential high burden and target for vaccination. Geospatial variation may vary over time (**Figure S8**, **Table S3**, and **Table S3**) and it will be necessary to keep updating the estimates using more recent information. In a similar vein, our study may help identify areas lacking data for improved surveillance. While surveillance studies provide the most reliable disease occurrence and incidence data, they are resource-intensive, and it is important to conduct surveillance in the area that could provide the most valuable information such as the Middle Africa (e.g., Democratic Republic of the Congo and Republic of the Congo) in which the occurrence data are scarce while the probability of occurrence was high for years since round 2016 (**Figure 6D**, **Figure S8**, and **Table S4**).

For peer review only

Our modeling analyses indicate that geospatial estimates of the host risk factors such as infection with HIV, malaria, or underweight are useful for predicting the geospatial distribution of iNTS occurrence while water and sanitation status may also have some smaller predictive capacity. In this regard, developing a risk index of iNTS based on a similar set of variables to what we have used has been attempted for nine sub-Saharan countries [36].

Our study had a number of limitations. First, the data points were few and especially we lacked data on the absence of iNTS disease. While occurrence data points greatly expanded those available from population-based incidence studies, cumulative occurrences from 2000 through 2020 only represented around 3% of the 20 km by 20 km grids of sub-Saharan Africa. A limited number of countries, primarily Malawi, contributed the majority of data, with some countries reporting none or few cases, potentially contributing to bias. Nonetheless, our model demonstrates high accuracy, indicated by an ROC curve area of 0.91 (Figure S7). The predictions (Figure 4) show areas with high iNTS disease probability, such as northern Central African Republic, western Zambia, and Cameroon, despite having little or no reported cases. Second, the reported location of iNTS disease occurrence was restricted to hospital-level or high-level administrative units and does not accurately reflect the location of NTS transmission. Hence, the estimated probability of occurrence might not reflect the true probability of iNTS pathogen transmission. Our approach was to assign a catchment area to the hospital and assume that occurrence could happen on any of the grid cells according to a decay function representing decreasing probability of visits with increasing distance from the hospital. This resulted in a dataset that reflected more frequent occurrences in areas close to the hospital than in areas far away. To mitigate this potential bias, we selected background points biased towards shorter distance to healthcare facilities. While occurrences were randomly located based on the exponential decay function, the geospatial and temporal patterns appeared to be robust against different parameterizations for the decay function (**Figure S9**). In addition, iNTS disease represented only a fraction of transmission of the pathogen [37] and thus it is likely that the estimates based on the disease will underestimate the true transmission of the pathogen. Third, the

For peer review only

frequency of occurrence is likely to be influenced by the frequency of publication in that occurrence is only possible when they are reported in the peer-reviewed journal. Therefore, areas in which iNTS was not studied or reported for any reason were underrepresented. Therefore, studies such as population-based surveillance will need to verify the inferred probability of occurrence. Fourth, although the BRT is a machine learning method known for its effective predictive capabilities, interpreting the association between explanatory covariates and the outcome, probability of occurrence of iNTS disease, remains challenging, as illustrated in **Figure 5B**. However, the findings on the variables are generally consistent with our knowledge on the risk factors for iNTS disease and we could still combine the knowledge on the probability of occurrence and the potential risk factors to prioritize our resources for more effective control of disease.

Conclusion

Our analysis suggests that the occurrence of iNTS disease varies greatly across sub-Saharan Africa, with certain regions being disproportionately affected. This geospatial heterogeneity is crucial to understand to effectively combat the iNTS disease. By identifying areas with high probability of occurrence, targeted surveillance can be conducted to better understand the incidence and the drivers of the disease in those regions. This information can then be used to develop intervention programs to prevent the spread of iNTS. Ultimately, a deeper understanding of the geographical distribution of iNTS can help to reduce its impact and improve public health outcomes in sub-Saharan Africa.

Funding

This project has received funding from the EU Horizon 2020 research and innovation programme under the project Vacc-iNTS (grant agreement number 815439).

Author contribution

For peer review only

Conceptualization: Jong-Hoon Kim, Florian Marks, Vacc-iNTS consortium

Data curation: Bieke Tack, Fabio Fiorino, Elena Pettini, Christian S. Marchello

Formal analysis: Jong-Hoon Kim

Funding acquisition: Florian Marks, Vacc-iNTS consortium collaborators

Investigation: Jong-Hoon Kim, Bieke Tack, Jan Jacobs, John A. Crump

Methodology: Jong-Hoon Kim, Bieke Tack

Resources: Jong-Hoon Kim, Florian Marks Vacc-iNTS consortium

Software: Jong-Hoon Kim

Supervision: Jong-Hoon Kim, Florian Marks, Vacc-iNTS consortium

Validation: Jong-Hoon Kim, Bieke Tack, Jan Jacobs, John A. Crump

Visualization: Jong-Hoon Kim

Writing – original draft: Jong-Hoon Kim, Bieke Tack, Jan Jacobs, John A. Crump

Writing – review & editing: Jong-Hoon Kim, Bieke Tack, Fabio Fiorino, Elena Pettini, Jan Jacobs, John A. Crump, Florian Marks, Vacc-iNTS consortium

Acknowledgement

We thank Vacc-iNTS team for technical support and continuous encouragement. Vacc-iNTS consortium collaborators include: Francis Agyapong (Kwame Nkrumah University of Science and Technology Kumasi), Francesco Berlanda Scorza (GSK Vaccines Institute for Global Health), Gianluca Breghi (Fondazione Achille Sclavo), Rocío Canals (GSK Vaccines Institute for Global Health), Melita A Gordon (University of Liverpool), Brama Hanumunthadu (University of Oxford), Samuel Kariuki (Kenya Medical Research Institute), Stefano Malvolti (MM Global Health Consulting), Carsten Mantel (MM Global Health Consulting), Donata Medaglini (Università di Siena and Sclavo Vaccines Association), Esther Muthumbi (KEMRI-Wellcome Trust Research Programme), Mercy Ngetich (Kenya Medical Research Institute), Tonney S. Nyirenda (University of Malawi), Ellis Owusu-Dabo (Kwame Nkrumah University of Science and Technology Kumasi), Maheshi Ramasamy (University of Oxford), J. Anthony

For peer review only

Scott (KEMRI-Wellcome Trust Research Programme), Bassiahi Abdramane Soura (University of Ouagadougou), Tiziana Spadafina (Sclavo Vaccines Association)

Competing interests

The authors declare that they have no competing interests.

Ethics statements

Patient consent for publication

Not applicable.

Data availability

All the codes and datasets are available on GitHub (https://github.com/kimfinale/iNTS_Mapping) and available upon request.

References

1. Stanaway JD, Parisi A, Sarkar K, Blacker BF, Reiner RC, Hay SI, et al. The global burden of non-typhoidal salmonella invasive disease: a systematic analysis for the Global Burden of Disease Study 2017. Lancet Infect Dis. 2019;19: 1312–1324. doi:10.1016/S1473-3099(19)30418-9

2. Marchello CS, Fiorino F, Pettini E, Crump JA, Vacc-iNTS Consortium Collaborators. Incidence of non-typhoidal Salmonella invasive disease: A systematic review and meta-analysis. J Infect. 2021;83: 523–532. doi:10.1016/j.jinf.2021.06.029

3. Marchello CS, Birkhold M, Crump JA, Vacc-iNTS consortium collaborators. Complications and mortality of non-typhoidal salmonella invasive disease: a global systematic review and meta-analysis. Lancet Infect Dis. 2022;22: 692–705. doi:10.1016/S1473-3099(21)00615-0

4. Crump JA, Sjölund-Karlsson M, Gordon MA, Parry CM. Epidemiology, Clinical Presentation, Laboratory Diagnosis, Antimicrobial Resistance, and Antimicrobial Management of Invasive Salmonella Infections. Clin Microbiol Rev. 2015;28: 901–937. doi:10.1128/CMR.00002-15

5. Gilchrist JJ, MacLennan CA. Invasive Nontyphoidal Salmonella Disease in Africa. EcoSal Plus. 2019;8. doi:10.1128/ecosalplus.ESP-0007-2018

6. Kingsley RA, Msefula CL, Thomson NR, Kariuki S, Holt KE, Gordon MA, et al. Epidemic multiple drug resistant Salmonella Typhimurium causing invasive disease in sub-Saharan Africa have a distinct genotype. Genome Res. 2009;19: 2279–2287. doi:10.1101/gr.091017.109

For peer review only

7. Chirwa EB, Dale H, Gordon MA, Ashton PM. What is the Source of Infections Causing Invasive Nontyphoidal Salmonella Disease? Open Forum Infectious Diseases. 2023;10: ofad086. doi:10.1093/ofid/ofad086

8. Ao TT, Feasey NA, Gordon MA, Keddy KH, Angulo FJ, Crump JA. Global burden of invasive nontyphoidal Salmonella disease, 2010(1). Emerg Infect Dis. 2015;21. doi:10.3201/eid2106.140999

9. Moher D, Liberati A, Tetzlaff J, Altman DG, PRISMA Group. Preferred reporting items for systematic reviews and meta-analyses: the PRISMA statement. PLoS Med. 2009;6: e1000097. doi:10.1371/journal.pmed.1000097

10. Ouzzani M, Hammady H, Fedorowicz Z, Elmagarmid A. Rayyan-a web and mobile app for systematic reviews. Syst Rev. 2016;5: 210. doi:10.1186/s13643-016-0384-4

11. Lessler J, Moore SM, Luquero FJ, McKay HS, Grais R, Henkens M, et al. Mapping the burden of cholera in sub-Saharan Africa and implications for control: an analysis of data across geographical scales. The Lancet. 2018;391: 1908–1915. doi:10.1016/S0140-6736(17)33050-7

12. Stock R. Distance and the utilization of health facilities in rural Nigeria. Soc Sci Med. 1983;17: 563–570. doi:10.1016/0277-9536(83)90298-8

13. Uche IV, MacLennan CA, Saul A. A Systematic Review of the Incidence, Risk Factors and Case Fatality Rates of Invasive Nontyphoidal Salmonella (iNTS) Disease in Africa (1966 to 2014). Baker S, editor. PLoS Negl Trop Dis. 2017;11: e0005118. doi:10.1371/journal.pntd.0005118

14. Dwyer-Lindgren L, Cork MA, Sligar A, Steuben KM, Wilson KF, Provost NR, et al. Mapping HIV prevalence in sub-Saharan Africa between 2000 and 2017. Nature. 2019;570: 189–193. doi:10.1038/s41586-019-1200-9

15. Weiss DJ, Lucas TCD, Nguyen M, Nandi AK, Bisanzio D, Battle KE, et al. Mapping the global prevalence, incidence, and mortality of Plasmodium falciparum, 2000-17: a spatial and temporal modelling study. Lancet. 2019;394: 322–331. doi:10.1016/S0140-6736(19)31097-9

16. Kinyoki DK, Osgood-Zimmerman AE, Pickering BV, Schaeffer LE, Marczak LB, Lazzar-Atwood A, et al. Mapping child growth failure across low- and middle-income countries. Nature. 2020;577: 231–234. doi:10.1038/s41586-019-1878-8

17. Berkley JA, Lowe BS, Mwangi I, Williams T, Bauni E, Mwarumba S, et al. Bacteremia among children admitted to a rural hospital in Kenya. N Engl J Med. 2005;352: 39–47. doi:10.1056/NEJMoa040275

18. Peters RPH, Zijlstra EE, Schijffelen MJ, Walsh AL, Joaki G, Kumwenda JJ, et al. A prospective study of bloodstream infections as cause of fever in Malawi: clinical predictors and implications for management. Trop Med Int Health. 2004;9: 928–934. doi:10.1111/j.1365-3156.2004.01288.x

19. Mandomando I, Macete E, Sigaúque B, Morais L, Quintó L, Sacarlal J, et al. Invasive non-typhoidal Salmonella in Mozambican children. Trop Med Int Health. 2009;14: 1467–1474. doi:10.1111/j.1365-3156.2009.02399.x

For peer review only

20. Sigaúque B, Roca A, Mandomando I, Morais L, Quintó L, Sacarlal J, et al. Community-acquired bacteremia among children admitted to a rural hospital in Mozambique. Pediatr Infect Dis J. 2009;28: 108–113. doi:10.1097/INF.0b013e318187a87d

21. Nielsen MV, Sarpong N, Krumkamp R, Dekker D, Loag W, Amemasor S, et al. Incidence and characteristics of bacteremia among children in rural Ghana. PLoS One. 2012;7: e44063. doi:10.1371/journal.pone.0044063

22. Biggs HM, Lester R, Nadjm B, Mtove G, Todd JE, Kinabo GD, et al. Invasive Salmonella infections in areas of high and low malaria transmission intensity in Tanzania. Clin Infect Dis. 2014;58: 638–647. doi:10.1093/cid/cit798

23. Graham SM, Walsh AL, Molyneux EM, Phiri AJ, Molyneux ME. Clinical presentation of non-typhoidal Salmonella bacteraemia in Malawian children. Trans R Soc Trop Med Hyg. 2000;94: 310–314. doi:10.1016/s0035-9203(00)90337-7

24. Deshpande A, Miller-Petrie MK, Lindstedt PA, Baumann MM, Johnson KB, Blacker BF, et al. Mapping geographical inequalities in access to drinking water and sanitation facilities in low-income and middle-income countries, 2000–17. Lancet Glob Health. 2020;8: e1162–e1185. doi:10.1016/S2214-109X(20)30278-3

25. Africa HIV Prevalence Geospatial Estimates 2000-2017. Available: https://ghdx.healthdata.org/record/ihme-data/africa-hiv-prevalence-geospatial-estimates-2000-2017

26. Malaria Atlas Project. Available: https://malariaatlas.org/

27. Low- and Middle-Income Country Child Growth Failure Geospatial Estimates 2000-2017. Available: https://ghdx.healthdata.org/record/ihme-data/lmic-child-growth-failure-geospatial-estimates-2000-2017

28. Low- and Middle-Income Country Drinking Water and Sanitation Facilities Access Geospatial Estimates 2000-2017. Available: https://ghdx.healthdata.org/record/ihme-data/lmic-wash-access-geospatial-estimates-2000-2017

29. Elith J, Leathwick JR, Hastie T. A working guide to boosted regression trees. Journal of Animal Ecology. 2008;77: 802–813. doi:10.1111/j.1365-2656.2008.01390.x

30. Barbet-Massin M, Jiguet F, Albert CH, Thuiller W. Selecting pseudo-absences for species distribution models: how, where and how many? Methods in Ecology and Evolution. 2012;3: 327–338. doi:10.1111/j.2041-210X.2011.00172.x

31. Weiss DJ, Nelson A, Vargas-Ruiz CA, Gligorić K, Bavadekar S, Gabrilovich E, et al. Global maps of travel time to healthcare facilities. Nat Med. 2020;26: 1835–1838. doi:10.1038/s41591-020-1059-1

32. Bradley AP. The use of the area under the ROC curve in the evaluation of machine learning algorithms. Pattern Recognition. 1997;30: 1145–1159. doi:10.1016/S0031-3203(96)00142-2

For peer review only

33. Kim J-H. Examining geospatial and temporal distribution of invasive non-typhoidal Salmonella disease occurrence in sub-Saharan Africa: A systematic review and modeling study. Available: https://github.com/kimfinale/iNTS_Mapping

34. Global Administrative Areas. [cited 1 Feb 2022]. Available: www.gadm.org

35. GlaxoSmithKline. A Phase 1/2a, Observer-blind, Randomized, Controlled, Two-stage, Multi-country Study to Evaluate the Safety, Reactogenicity, and Immune Response of the Trivalent Vaccine Against Invasive Nontyphoidal Salmonella (iNTS) and Typhoid Fever in Healthy European and African Adults. clinicaltrials.gov; 2022 Oct. Report No.: NCT05480800. Available: https://clinicaltrials.gov/ct2/show/NCT05480800

36. Lee J-S, Mogasale V, Marks F, Kim J. Geographical distribution of risk factors for invasive non-typhoidal Salmonella at the subnational boundary level in sub-Saharan Africa. BMC Infectious Diseases. 2021;21: 529. doi:10.1186/s12879-021-06198-1

37. Invasive multidrug-resistant non-typhoidal Salmonella infections in Africa: zoonotic or anthroponotic transmission? In: ScienceOpen [Internet]. [cited 7 Apr 2023]. doi:10.1099/jmm.0.46375-0

For peer review only

Figure legends

Figure 1. PRISMA flow diagram for the systematic review of geospatial distribution of invasive non-typhoidal Salmonella (iNTS) occurrence in sub-Saharan Africa, 2000-2020.

Figure 2. Data of human iNTS infection, sub-Saharan Africa, 2000-2020. (A) represents the number of reports by country and year of publication. (B) represents the number of reported cases by country and year of data collection. CAR = Central African Republic; DR Congo = Democratic Republic of the Congo

Figure 3. iNTS Occurrence on 20 km by 20 km grids. (A) and (B) represent typical occurrences observed in 2010 and during the entire search period (2000-2020), respectively. Thick and thin lines indicate borders of the administrative unit level 0 (i.e., country) and the administrative unit level 1 (i.e., province).

Figure 4. Estimated probability of iNTS disease occurrence, sub-Saharan Africa, 2017. Thick and thin lines indicate borders of country and province, respectively. (A), (B), and (C) show mean, 2.5^{th} and 97.5^{th} values on 20 km by 20 km grids. (D) shows the median values aggregated at the administrative unit level 1 (i.e., province or districts).

Figure 5. Impact of covariates on the probability of occurrence. Results are based on 400 simulation runs. (A) represents relative importance of covariates on the probability of occurrence. Bar plots and error bars indicate mean with 95% confidence intervals. (B) represents probability of occurrence in response to variables. The units for sanitation, water, and HIV prevalence are expressed as percentages, ranging from 0 to 100%. In contrast, *Plasmodium falciparum* (Pf) incidence and underweight prevalence are measured

For peer review only

as proportions, with values ranging from 0 to 1. Smoothed line (blue) and 95% confidence interval (red) bands were based on LOESS (LOcally Estimated Scatterplot Smoothing).

Figure 6. Probability of occurrence at the 20 km by 20 km grid level and averaged across the country level. (A), (B), and (C) represent the mean probability of occurrence for 2000, 2010, and 2020, respectively. (D) represents the values aggregated at the country level for 2000-2020. CAR=Central African Republic; DR Congo = Democratic Republic of the Congo; Congo=Republic of the Congo

For peer review only

Figure 1. PRISMA flow diagram for the systematic review of geospatial distribution of invasive non-typhoidal Salmonella (iNTS) occurrence in sub-Saharan Africa, 2000-2020.

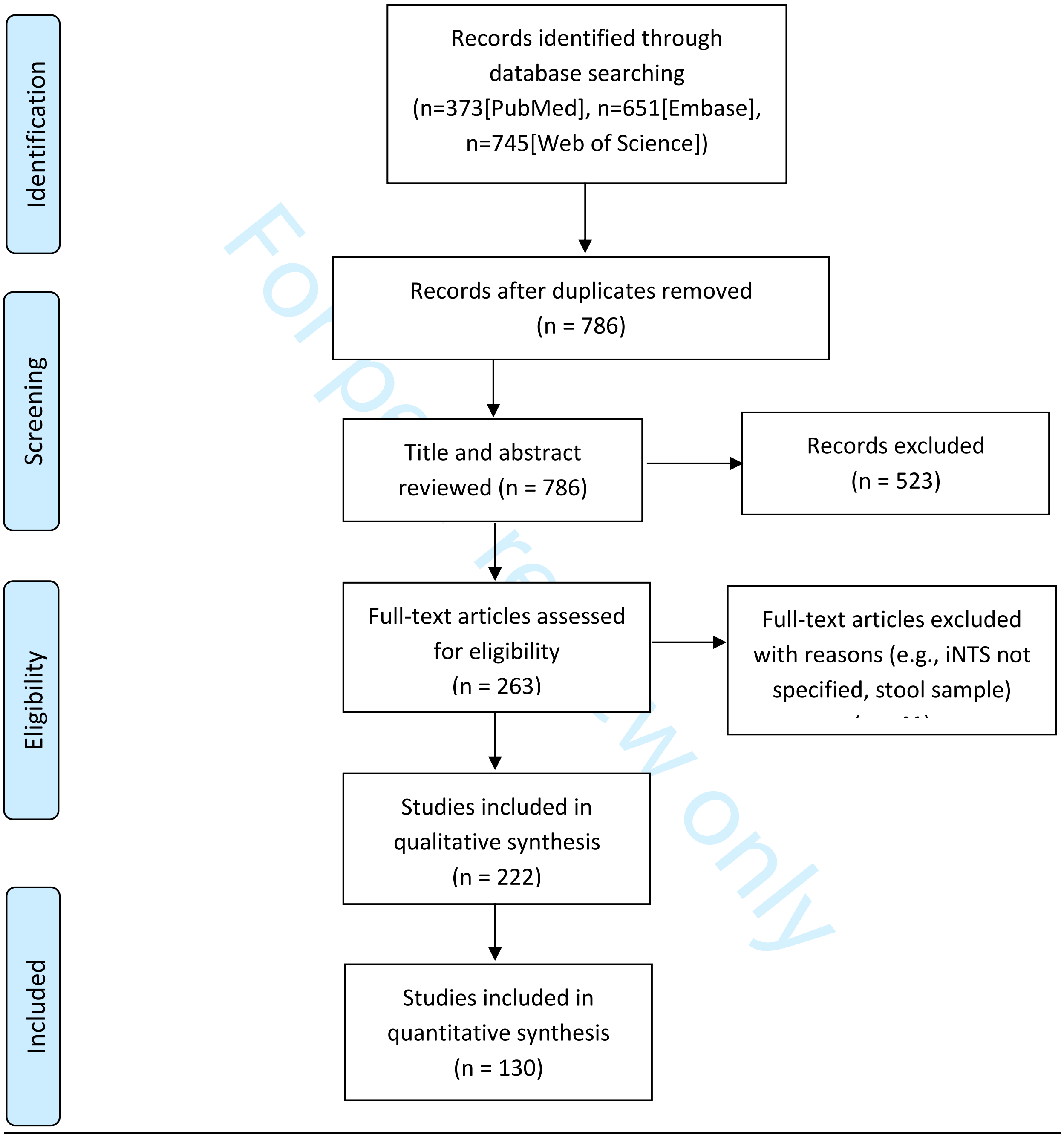

For peer review only

For peer review only - http://bmjopen.bmj.com/site/about/guidelines.xhtml

Figure 2. Data of human iNTS infection, sub-Saharan Africa, 2000-2020. (A) represents the number of reports by country and year of publication. (B) represents the number of reported cases by country and year of data collection. CAR = Central African Republic; DR Congo = Democratic Republic of the Congo

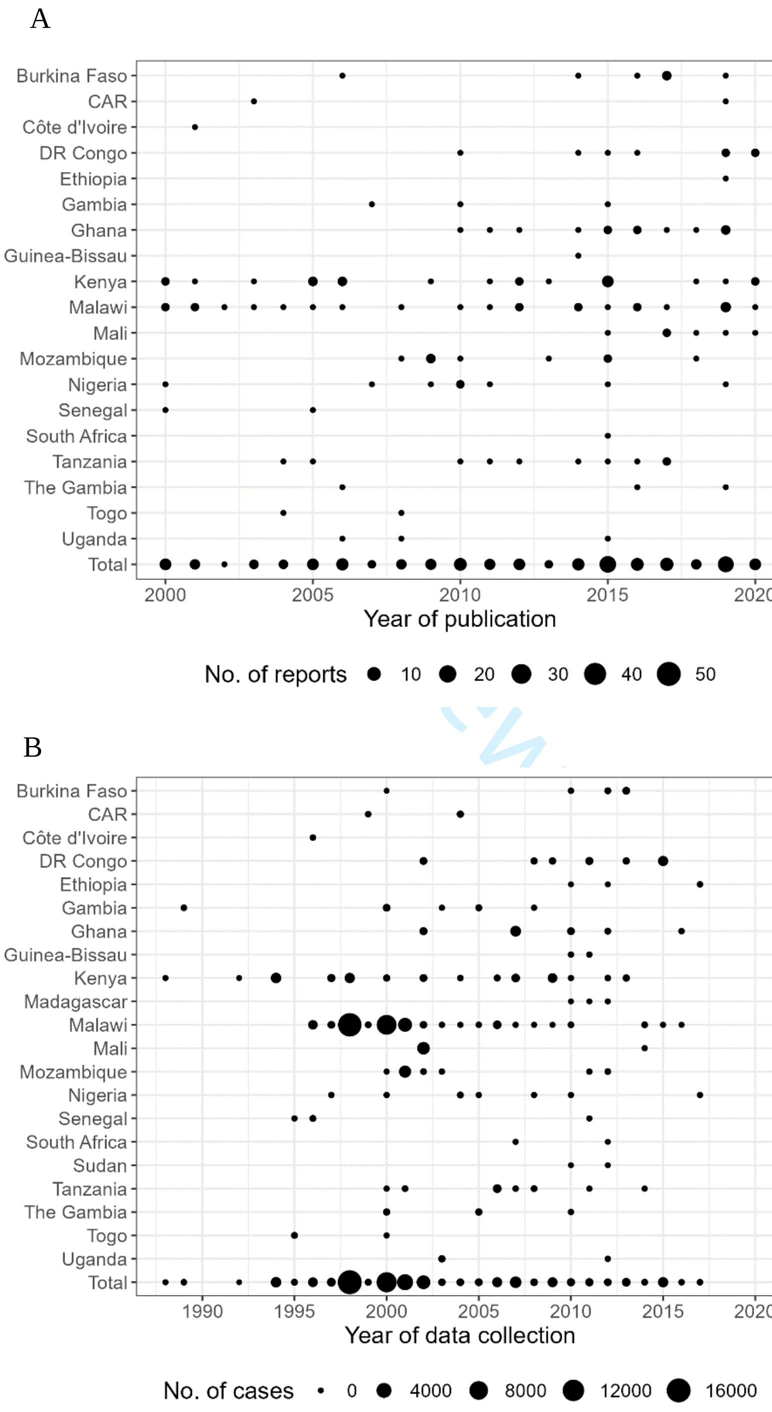

Figure 3. iNTS Occurrence on 20 km by 20 km grids. (A) and (B) represent typical occurrences observed in 2010 and during the entire search period (2000-2020), respectively. Thick and thin lines indicate borders of the administrative unit level 0 (i.e., country) and the administrative unit level 1 (i.e., province).

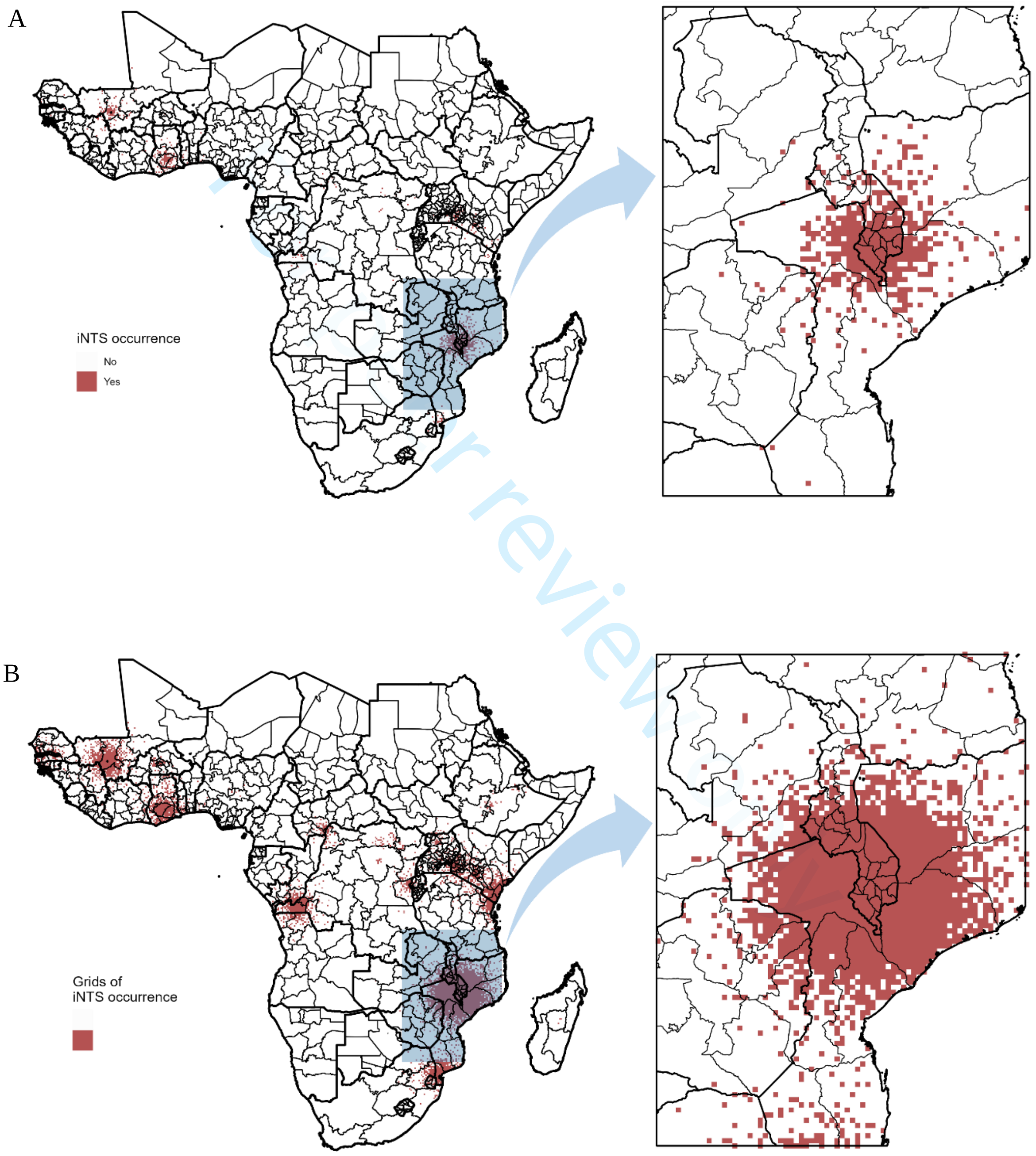

Figure 4. Estimated probability of iNTS disease occurrence, sub-Saharan Africa, 2017. Thick and thin lines indicate borders of country and province, respectively. (A), (B), and (C) show mean, 2.5th and 97.5th values on 20 km by 20 km grids. (D) shows the median values aggregated at the administrative unit level 1 (i.e., province or districts).

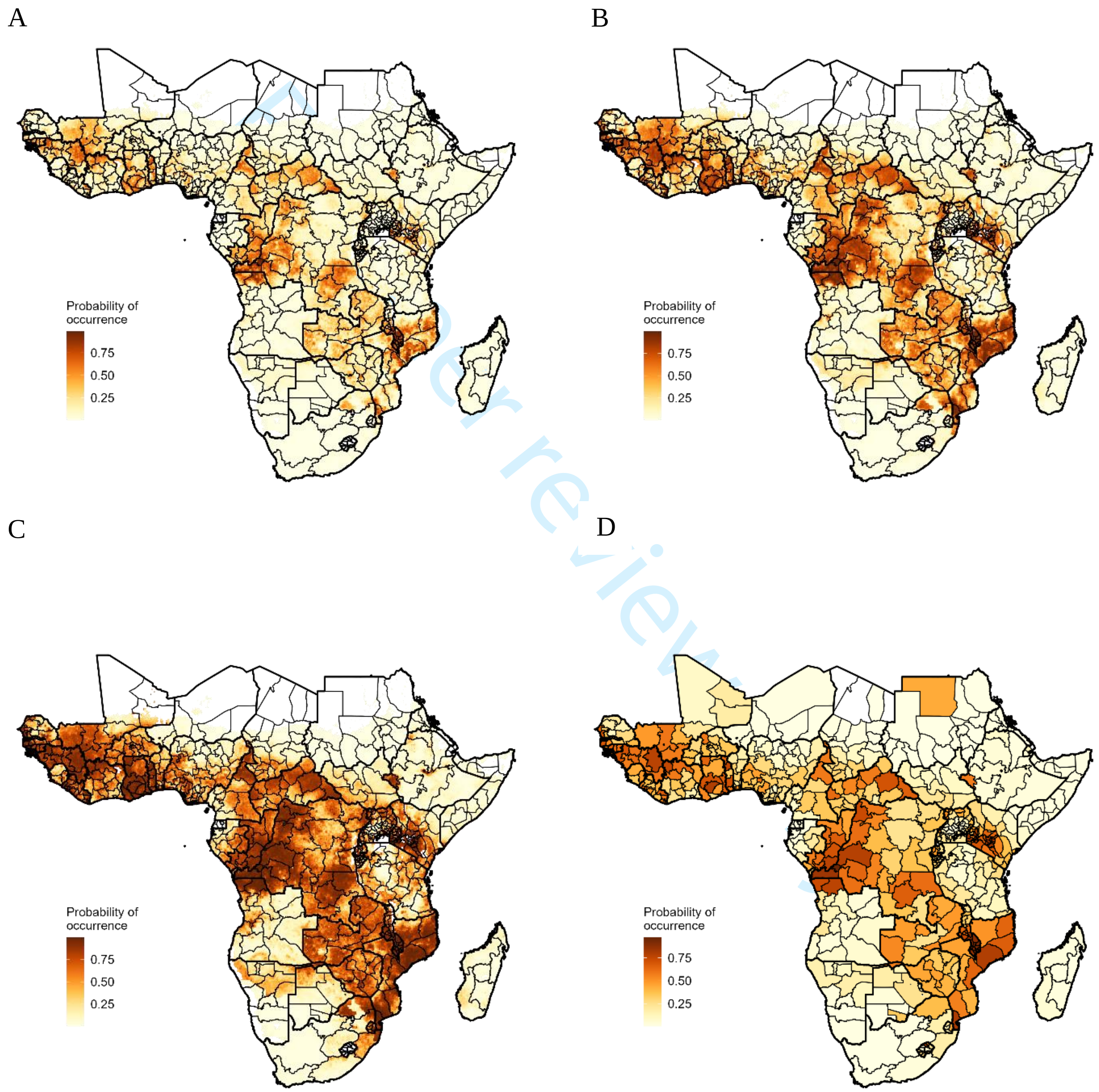

Figure 5. Impact of covariates on the probability of occurrence. Results are based on 400 simulation runs. (A) represents relative importance of covariates on the probability of occurrence. Bar plots and error bars indicate mean with 95% confidence intervals. (B) represents probability of occurrence in response to variables. The units for sanitation, water, and HIV prevalence are expressed as percentages, ranging from 0 to 100%. In contrast, *Plasmodium falciparum* (Pf) incidence and underweight prevalence are measured as proportions, with values ranging from 0 to 1. Smoothed line (blue) and 95% confidence interval (red) bands were based on LOESS (LOcally Estimated Scatterplot Smoothing).

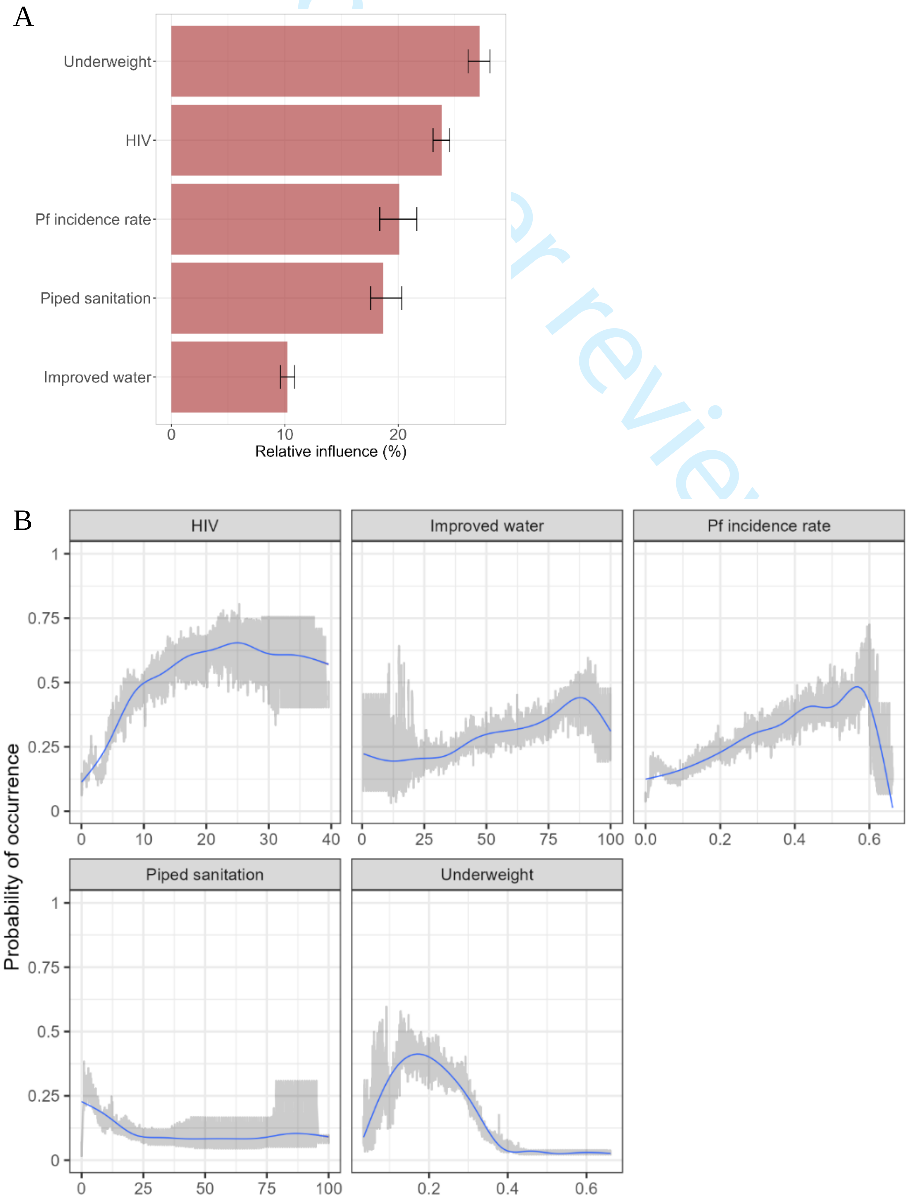

Figure 6. Probability of occurrence at the 20 km by 20 km grid level and averaged across the country level. (A), (B), and (C) represent the mean probability of occurrence for 2000, 2010, and 2020, respectively. (D) represents the values aggregated at the country level for 2000-2020. CAR=Central African Republic; DR Congo = Democratic Republic of the Congo; Congo=Republic of the Congo

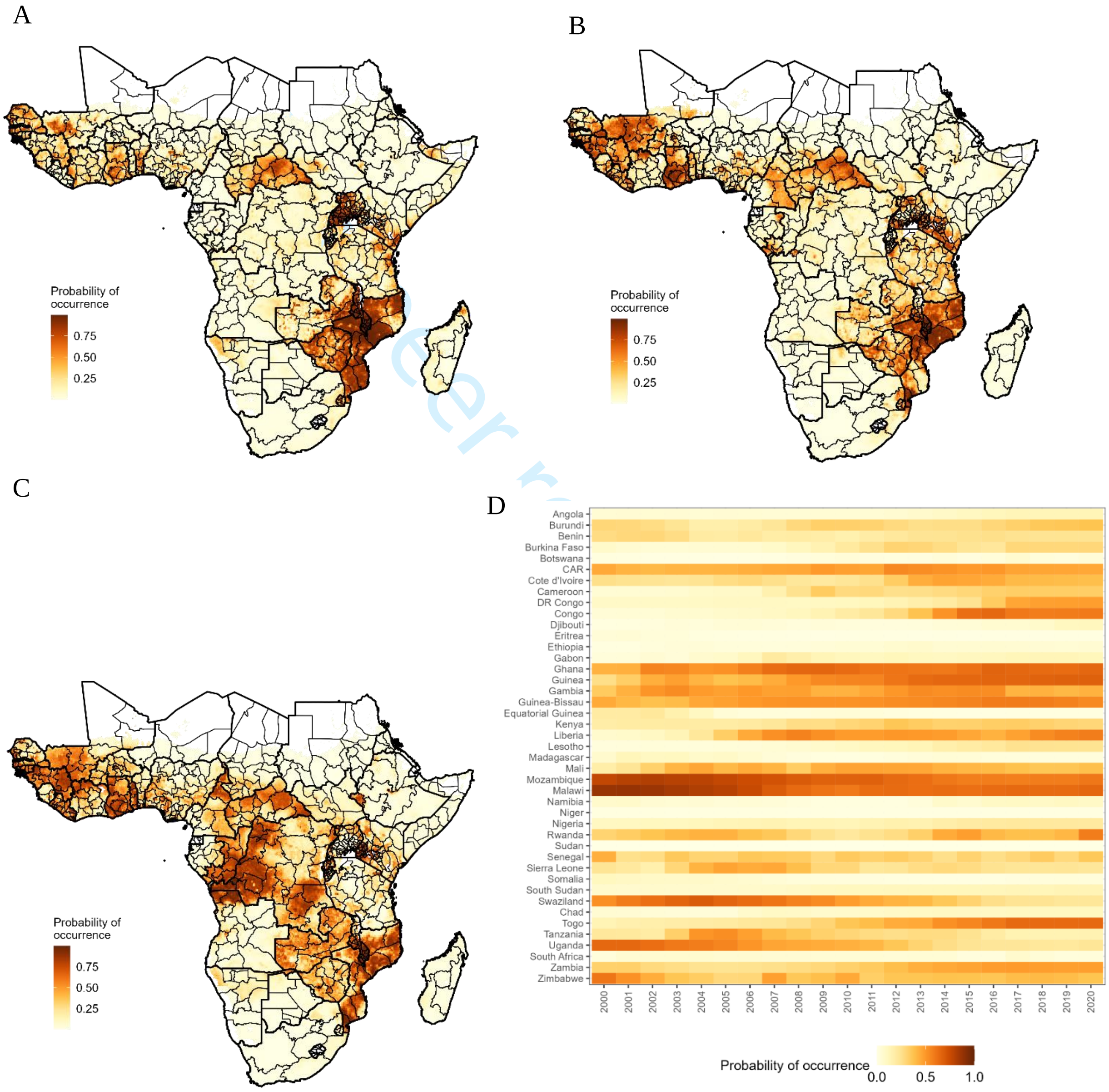

Supplementary Material

S1. Search terms

1. PubMed

(((nontyph*[Tiab] OR non-typh*[Tiab] OR group[Tiab] OR typhimurium[Tiab] OR enteritidis[Tiab] OR Heidelberg[Tiab] OR Dublin[Tiab] OR Choleraesuis[Tiab] OR Newport[Tiab] OR Virchow[Tiab] OR Concord[Tiab] OR Brancaster[Tiab] OR freetown[Tiab] OR Infantis[Tiab] OR Isangi[Tiab]) AND (salmonella[Tiab] OR salmonellosis[Tiab])) OR iNTS[Tiab])

AND

("Bacteremia"[Mesh] OR bacteremia [Tiab] OR bacteraemia [Tiab] OR "Sepsis"[Mesh] OR sepsis [Tiab] OR septic*[Tiab] OR "Invasive"[Tiab] OR bloodstream*[Tiab] OR “blood culture”[Tiab] OR “blood-culture”[Tiab] OR hemoculture OR incidence OR epidemiolog* OR burden OR case OR infect* OR prevalen*[Tiab])

AND

hasabstract

AND

“Humans”[Mesh]

AND

("2000/01/01"[Dp] : "2020/12/31"[Dp])

AND

("Africa South of the Sahara"[Mesh] OR "sub-saharan Africa"[Tiab] OR "subsaharan Africa"[Tiab] OR "Sub Saharan Africa"[Tiab] OR "Africa, Central"[Mesh] OR "Central Africa"[Tiab] OR "Cameroon"[Mesh] OR "Cameroon"[Tiab] OR "Central African Republic"[Mesh] OR "Central African Republic"[Tiab] OR "Chad"[Mesh] OR "Chad"[Tiab] OR "Congo"[Mesh] OR "Congo"[Tiab] OR "Democratic Republic of the Congo"[Mesh] OR "Democratic Republic of the Congo"[Tiab] OR "Equatorial Guinea"[Mesh] OR "Equatorial Guinea"[Tiab] OR "Gabon"[Mesh] OR "Gabon"[Tiab] OR "Sao Tome and Principe"[Mesh] OR "Sao Tome and Principe"[Tiab] OR "Africa, Eastern"[Mesh] OR "Eastern Africa"[Tiab] OR "East Africa"[Tiab] OR "Burundi"[Mesh] OR "Burundi"[Tiab] OR "Djibouti"[Mesh] OR "Djibouti"[Tiab] OR "Eritrea"[Mesh] OR "Eritrea"[Tiab] OR "Ethiopia"[Mesh] OR "Ethiopia" [Tiab] OR "Kenya"[Mesh] OR "Kenya" [Tiab] OR "Rwanda"[Mesh] OR "Rwanda" [Tiab] OR "Somalia"[Mesh] OR "Somalia"[Tiab] OR "South Sudan"[Mesh] OR "South Sudan"[Tiab] OR "Sudan"[Mesh] OR "Sudan"[Tiab] OR "Tanzania"[Mesh] OR "Tanzania"[Tiab] OR "Uganda"[Mesh] OR "Uganda"[Tiab] OR "Africa,

For peer review only

Southern"[Mesh] OR "Southern Africa"[Tiab] OR "South Africa"[Tiab] OR "Angola"[Mesh] OR "Angola"[Tiab] OR "Botswana"[Mesh] OR "Botswana"[Tiab] OR "Lesotho"[Mesh] OR "Lesotho"[Tiab] OR "Malawi"[Mesh] OR "Malawi"[Tiab] OR "Mozambique"[Mesh] OR "Mozambique"[Tiab] OR "Namibia"[Mesh] OR "Namibia"[Tiab] OR "South Africa"[Mesh] OR "South Africa"[Tiab] OR "Swaziland"[Mesh] OR "Swaziland"[Tiab] OR "Zambia"[Mesh] OR "Zambia"[Tiab] OR "Zimbabwe"[Mesh] OR "Zimbabwe"[Tiab] OR "Africa, Western"[Mesh] OR "Western Africa"[Tiab] OR "West Africa"[Tiab] OR "Benin"[Mesh] OR "Benin"[Tiab] OR "Burkina Faso"[Mesh] OR "Burkina Faso"[Tiab] OR "Cabo Verde"[Mesh] OR "Cabo Verde"[Tiab] OR "Cote d'Ivoire"[Mesh] OR "Cote d'Ivoire"[Tiab] OR "Gambia"[Mesh] OR "Gambia"[Tiab] OR "Ghana"[Mesh] OR "Ghana"[Tiab] OR "Guinea"[Mesh] OR "Guinea"[Tiab] OR "Guinea-Bissau"[Mesh] OR "Guinea-Bissau"[Tiab] OR "Liberia"[Mesh] OR "Liberia"[Tiab] OR "Mali"[Mesh] OR "Mali"[Tiab] OR "Mauritania"[Mesh] OR "Mauritania"[Tiab] OR "Niger"[Mesh] OR "Niger"[Tiab] OR "Nigeria"[Mesh] OR "Nigeria"[Tiab] OR "Senegal"[Mesh] OR "Senegal"[Tiab] OR "Sierra Leone"[Mesh] OR "Sierra Leone"[Tiab] OR "Togo"[Mesh] OR "Togo"[Tiab] OR "Comoros"[Mesh] OR "Comoros"[Tiab] OR "Mayotte"[Tiab] OR "Madagascar"[Mesh] OR "Madagascar"[Tiab] OR "Sahel"[Tiab])

2. Embase

(((‘nontyph*’:ti,ab OR ‘non-typh*’:ti,ab OR ‘group’:ti,ab OR ‘typhimurium’:ti,ab OR ‘enteritidis’:ti,ab OR ‘Heidelberg’:ti,ab OR ‘Dublin’:ti,ab OR ‘Choleraesuis’:ti,ab OR ‘Newport’:ti,ab OR ’Virchow’:ti,ab OR ‘Concord’:ti,ab OR ‘Brancaster’:ti,ab OR ‘freetown’:ti,ab OR ‘Infantis’:ti,ab OR ‘Isangi’:ti,ab) AND (‘salmonella’:ti,ab OR ‘salmonellosis’:ti,ab)) OR ‘iNTS’:ti,ab)

AND

(‘bacteremia’:ti,ab OR ‘sepsis’:ti,ab OR ‘septic*’:ti,ab OR ‘Invasive’:ti,ab OR ‘bloodstream*’:ti,ab OR ‘blood culture’:ti,ab OR ‘blood-culture’:ti,ab OR ‘hemoculture’:ti,ab OR ‘incidence’:ti,ab OR ‘epidemiolog*’:ti,ab OR ‘burden’:ti,ab OR ‘case’:ti,ab OR ‘infect*’:ti,ab OR ‘prevalen*’:ti,ab)

AND

[2000-2020]/py

AND

'Africa south of the Sahara'/exp OR ‘Sub-Saharan Africa’:ti,ab OR ‘Subsaharan Africa’:ti,ab OR ‘Sub Saharan Africa’:ti,ab OR 'Central Africa'/exp OR 'Central Africa':ti,ab OR 'Cameroon'/exp OR 'Cameroon':ti,ab OR 'Central African Republic'/exp OR 'Central African Republic':ti,ab OR 'Chad'/exp OR 'Chad':ti,ab OR 'Congo'/exp OR 'Congo':ti,ab OR 'Democratic Republic Congo'/exp OR 'Democratic Republic Congo':ti,ab OR 'Equatorial Guinea'/exp OR 'Equatorial Guinea':ti,ab OR 'Gabon'/exp OR 'Gabon':ti,ab OR 'Sao Tome and Principe'/exp OR 'Sao Tome and Principe':ti,ab OR ‘Eastern Africa’:ti,ab OR ‘East Africa’:ti,ab OR 'Burundi'/exp OR 'Burundi':ti,ab OR 'Djibouti'/exp OR 'Djibouti':ti,ab OR 'Eritrea'/exp OR 'Eritrea':ti,ab OR 'Ethiopia'/exp OR 'Ethiopia':ti,ab OR 'Kenya'/exp OR 'Kenya':ti,ab OR 'Rwanda'/exp OR 'Rwanda':ti,ab OR 'Somalia'/exp OR 'Somalia':ti,ab OR 'South Sudan'/exp OR 'South

For peer review only

Sudan':ti,ab OR 'Sudan'/exp OR 'Sudan':ti,ab OR 'Tanzania'/exp OR 'Tanzania':ti,ab OR 'Uganda'/exp OR 'Uganda':ti,ab OR 'Southern Africa':ti,ab OR 'South Africa':ti,ab OR 'Angola'/exp OR 'Angola':ti,ab OR 'Botswana'/exp OR 'Botswana':ti,ab OR 'Lesotho'/exp OR 'Lesotho':ti,ab OR 'Malawi'/exp OR 'Malawi':ti,ab OR 'Mozambique'/exp OR 'Mozambique':ti,ab OR 'Namibia'/exp OR 'Namibia':ti,ab OR 'South Africa'/exp OR 'South Africa':ti,ab OR 'Swaziland'/exp OR 'Swaziland':ti,ab OR 'Zambia'/exp OR 'Zambia':ti,ab OR 'Zimbabwe'/exp OR 'Zimbabwe':ti,ab OR 'Western Africa':ti,ab OR 'West Africa':ti,ab OR 'Benin'/exp OR 'Benin':ti,ab OR 'Burkina Faso'/exp OR 'Burkina Faso':ti,ab OR 'Cape Verde'/exp OR 'Cape Verde':ti,ab OR 'Cote d`Ivoire'/exp OR 'Cote d`Ivoire':ti,ab OR 'Gambia'/exp OR 'Gambia':ti,ab OR 'Ghana'/exp OR 'Ghana':ti,ab OR 'Guinea'/exp OR 'Guinea':ti,ab OR 'Guinea-Bissau'/exp OR 'Guinea-Bissau':ti,ab OR 'Liberia'/exp OR 'Liberia':ti,ab OR 'Mali'/exp OR 'Mali':ti,ab OR 'Mauritania'/exp OR 'Mauritania':ti,ab OR 'Niger'/exp OR 'Niger':ti,ab OR 'Nigeria'/exp OR 'Nigeria':ti,ab OR 'Senegal'/exp OR 'Senegal':ti,ab OR 'Sierra Leone'/exp OR 'Sierra Leone':ti,ab OR 'Togo'/exp OR 'Togo':ti,ab OR 'Comoros'/exp OR 'Comoros':ti,ab OR 'Mayotte'/exp OR 'Mayotte':ti,ab OR 'Madagascar'/exp OR 'Madagascar':ti,ab OR 'Sahel'/exp OR 'Sahel':ti,ab

3. Web of Science

(AB=(((nontyph* OR non-typh* OR group OR typhimurium OR enteritidis OR Heidelberg OR Dublin OR Choleraesuis OR Newport OR Virchow OR Concord OR Brancaster OR freetown OR Infantis OR Isangi) AND (salmonella OR salmonellosis)) OR iNTS)

AND

AB=(bacteremia OR sepsis OR septic* OR Invasive OR bloodstream* OR blood culture OR blood-culture OR hemoculture OR incidence OR epidemiolog* OR burden OR case OR infect* OR prevalen*)

AND

AB=("Sub-Saharan Africa" OR "Subsaharan Africa" OR "Sub Saharan Africa" OR "Central Africa" OR Cameroon OR "Central African Republic" OR Chad OR Congo OR "Democratic Republic Congo" OR "Equatorial Guinea" OR Gabon OR "Sao Tome and Principe" OR "Eastern Africa" OR "East Africa" OR Burundi OR Djibouti OR Eritrea OR Ethiopia OR Kenya OR Rwanda OR Somalia OR "South Sudan" OR Sudan OR Tanzania OR Uganda OR "Southern Africa" OR "South Africa" OR Angola OR Botswana OR Lesotho OR Malawi OR Mozambique OR Namibia OR Swaziland OR Zambia OR Zimbabwe OR "Western Africa" OR "West Africa" OR Benin OR Burkina Faso OR "Cape Verde" OR "Cote d`Ivoire" OR Gambia OR Ghana OR Guinea OR "Guinea-Bissau" OR Liberia OR Mali OR Mauritania OR Niger OR Nigeria OR Senegal OR "Sierra Leone" OR Togo OR Comoros OR Mayotte OR Madagascar OR Sahel))

OR

(TI=(((nontyph* OR non-typh* OR group OR typhimurium OR enteritidis OR Heidelberg OR Dublin OR Choleraesuis OR Newport OR Virchow OR Concord OR Brancaster OR freetown OR Infantis OR Isangi) AND (salmonella OR salmonellosis)) OR iNTS)

AND

For peer review only - http://bmjopen.bmj.com/site/about/guidelines.xhtml

TI=(bacteremia OR sepsis OR septic* OR Invasive OR bloodstream* OR blood culture OR blood-culture OR hemoculture OR incidence OR epidemiolog* OR burden OR case OR infect* OR prevalen*)

AND

TI=("Sub-Saharan Africa" OR "Subsaharan Africa" OR "Sub Saharan Africa" OR "Central Africa" OR Cameroon OR "Central African Republic" OR Chad OR Congo OR "Democratic Republic Congo" OR "Equatorial Guinea" OR Gabon OR "Sao Tome and Principe" OR "Eastern Africa" OR "East Africa" OR Burundi OR Djibouti OR Eritrea OR Ethiopia OR Kenya OR Rwanda OR Somalia OR "South Sudan" OR Sudan OR Tanzania OR Uganda OR "Southern Africa" OR "South Africa" OR Angola OR Botswana OR Lesotho OR Malawi OR Mozambique OR Namibia OR Swaziland OR Zambia OR Zimbabwe OR "Western Africa" OR "West Africa" OR Benin OR Burkina Faso OR "Cape Verde" OR "Cote d`Ivoire" OR Gambia OR Ghana OR Guinea OR "Guinea-Bissau" OR Liberia OR Mali OR Mauritania OR Niger OR Nigeria OR Senegal OR "Sierra Leone" OR Togo OR Comoros OR Mayotte OR Madagascar OR Sahel))

S2. Explanatory covariates

For peer review only

Figure S1. Mean estimates for the prevalence of HIV infection among 15-45 year olds in sub-Saharan Africa in 2017 [1].

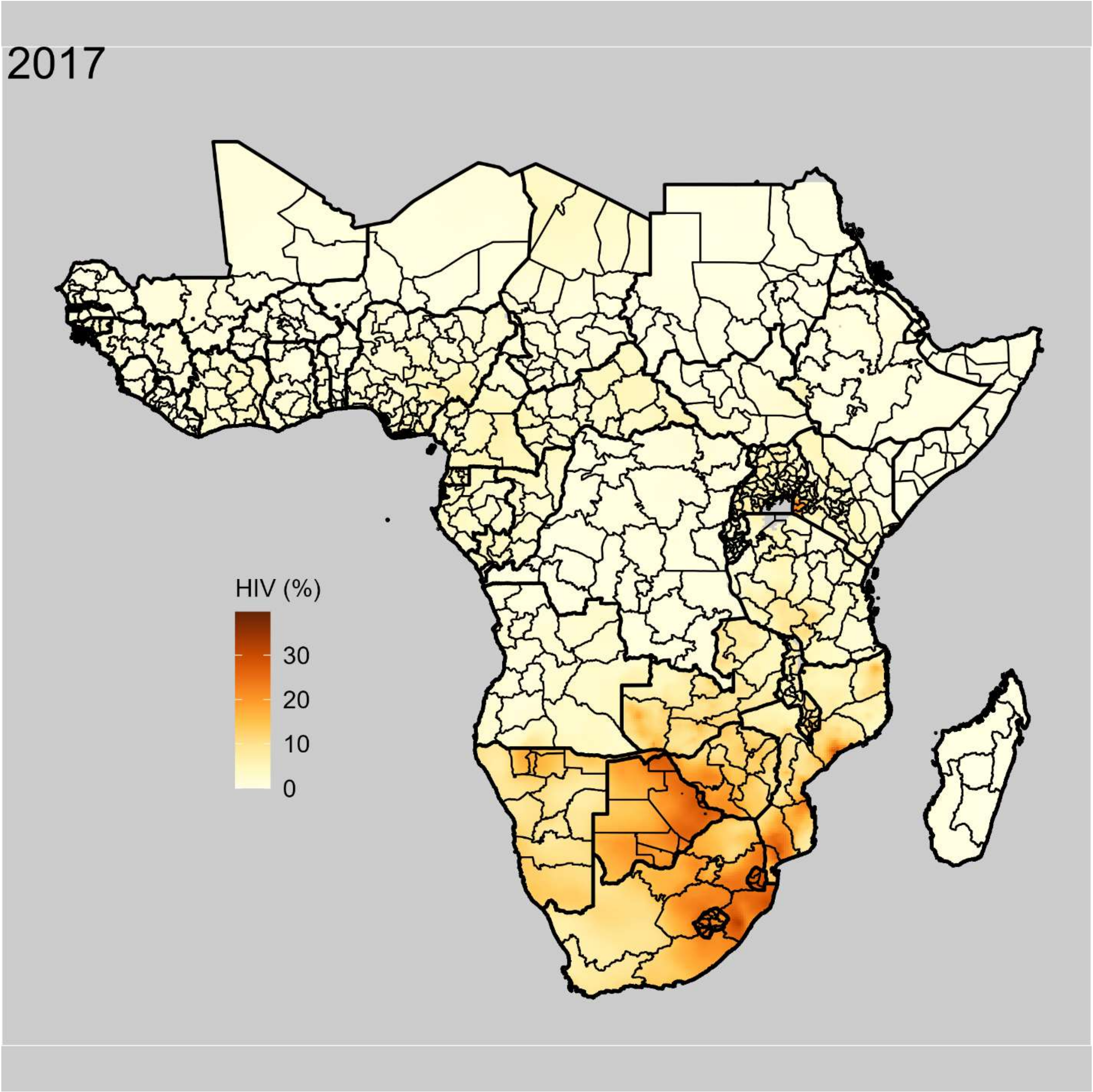

Figure S2. Incidence rate of *Plasmodium Falciparum* [2], 2017. The values indicate the number of newly diagnosed Plasmodium falciparum cases per person in 2017. Areas for which data are not available are not colored.

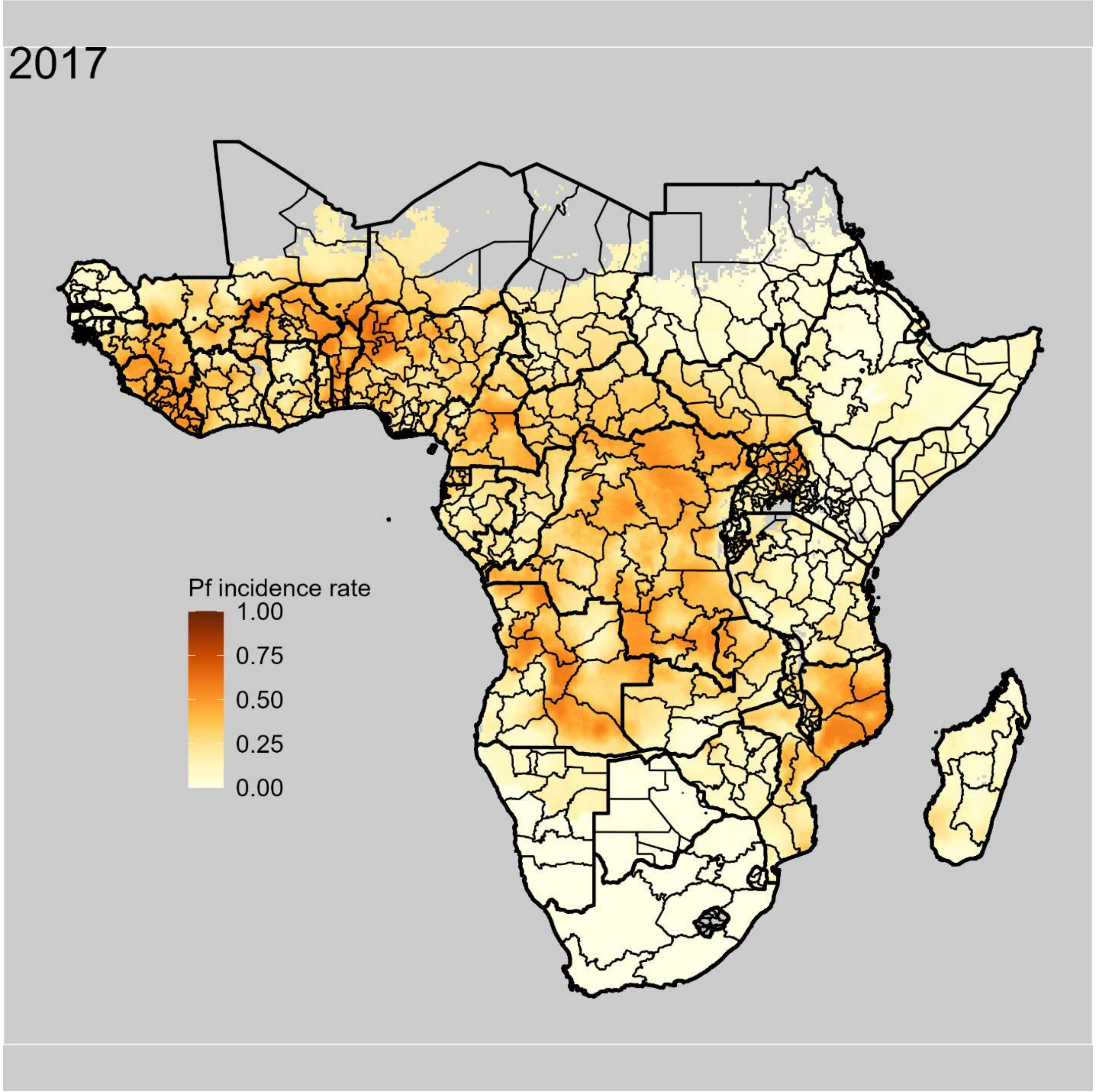

Figure S3. Prevalence of underweight [3].

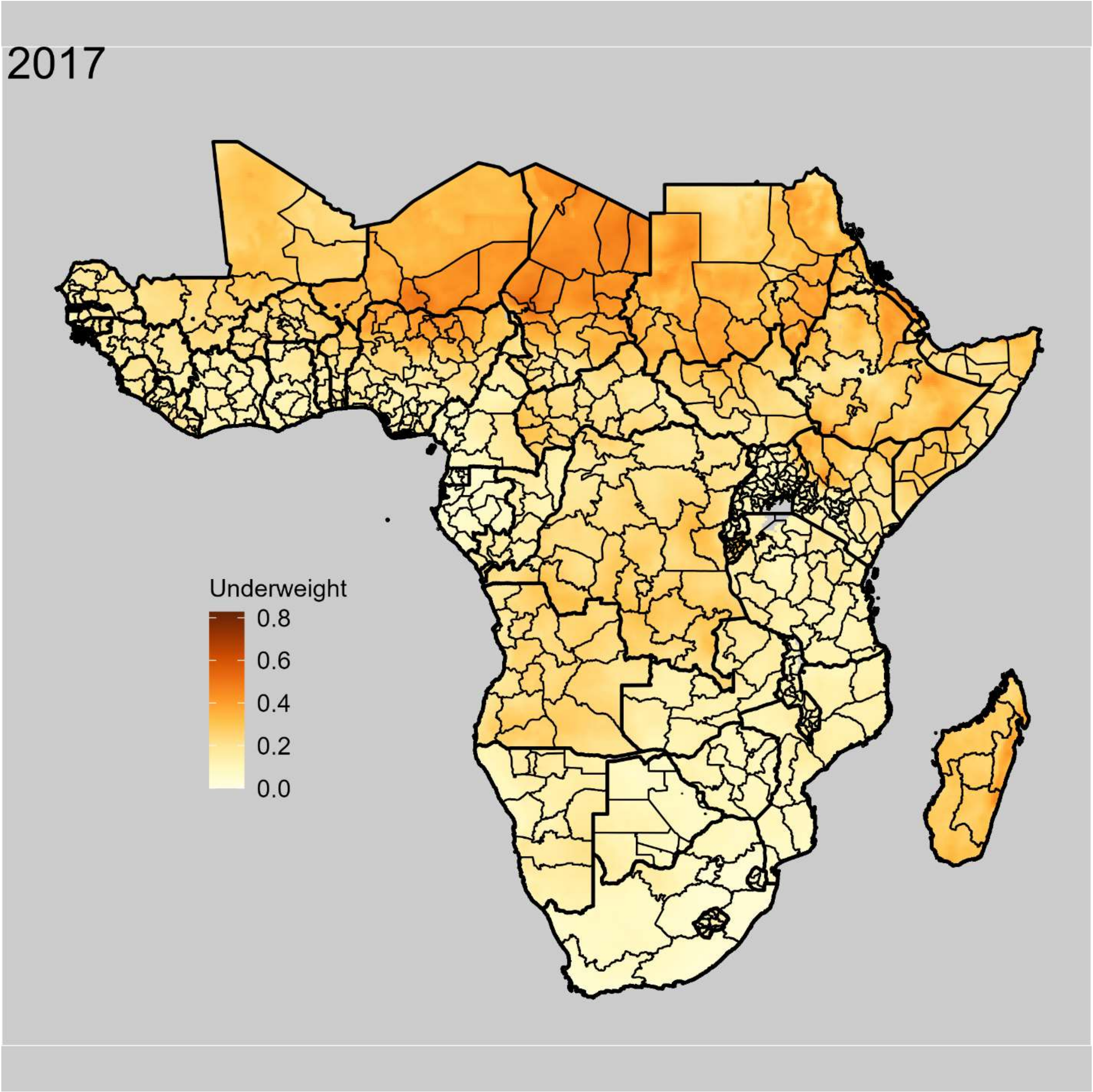

Figure S4. Percentage access to improved drinking water source. The values are based on the estimates on the mean access to any improved water sources on 5 km by 5 km grids [4]. Areas for which data are not available are not colored.

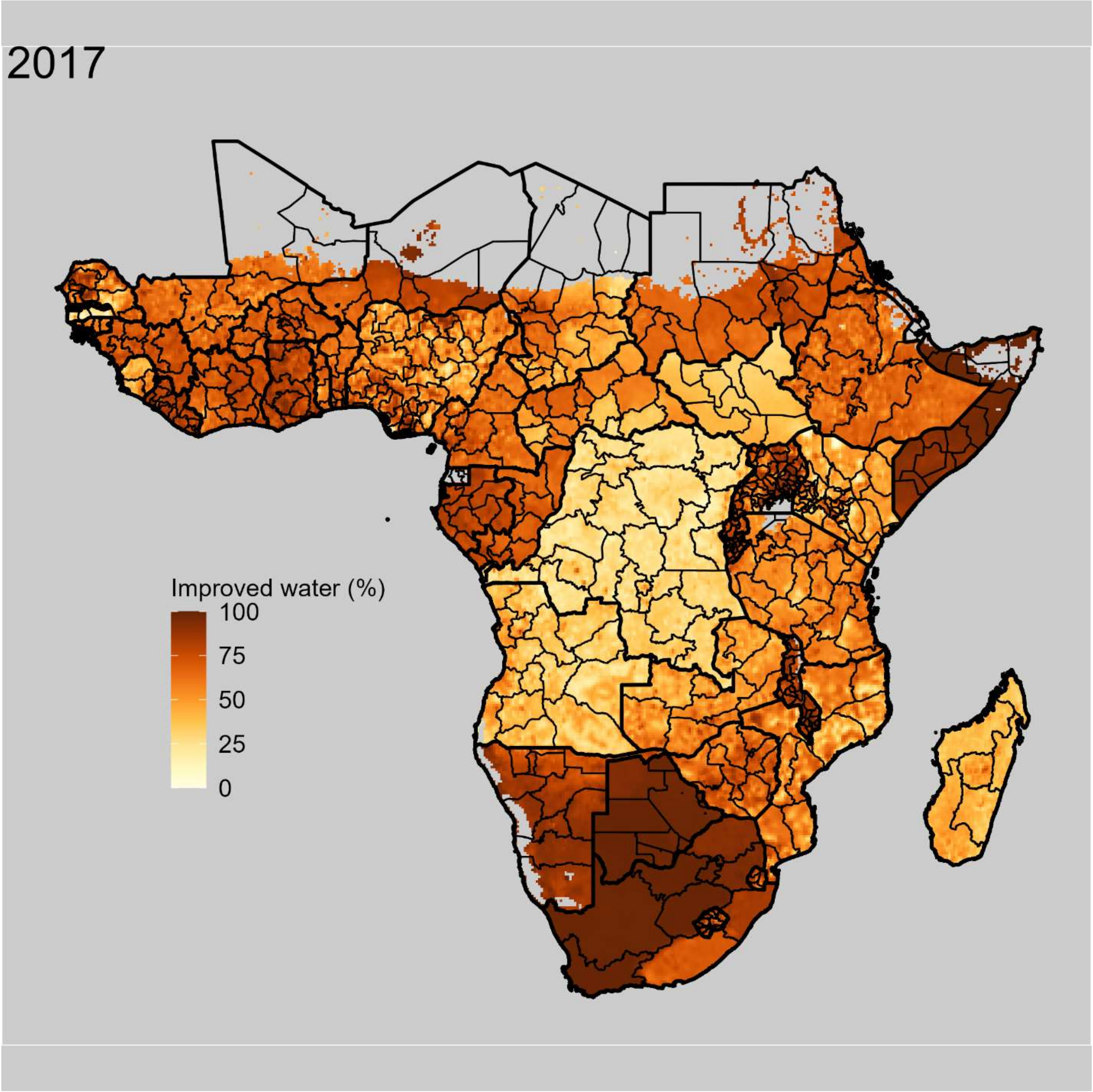

Figure S5. Percentage access to improved sanitation facilities. The values are based on the recent estimates on the mean access to any improved sanitation facilities on 5 km by 5 km grids [4]. Areas for which data are not available are not colored.

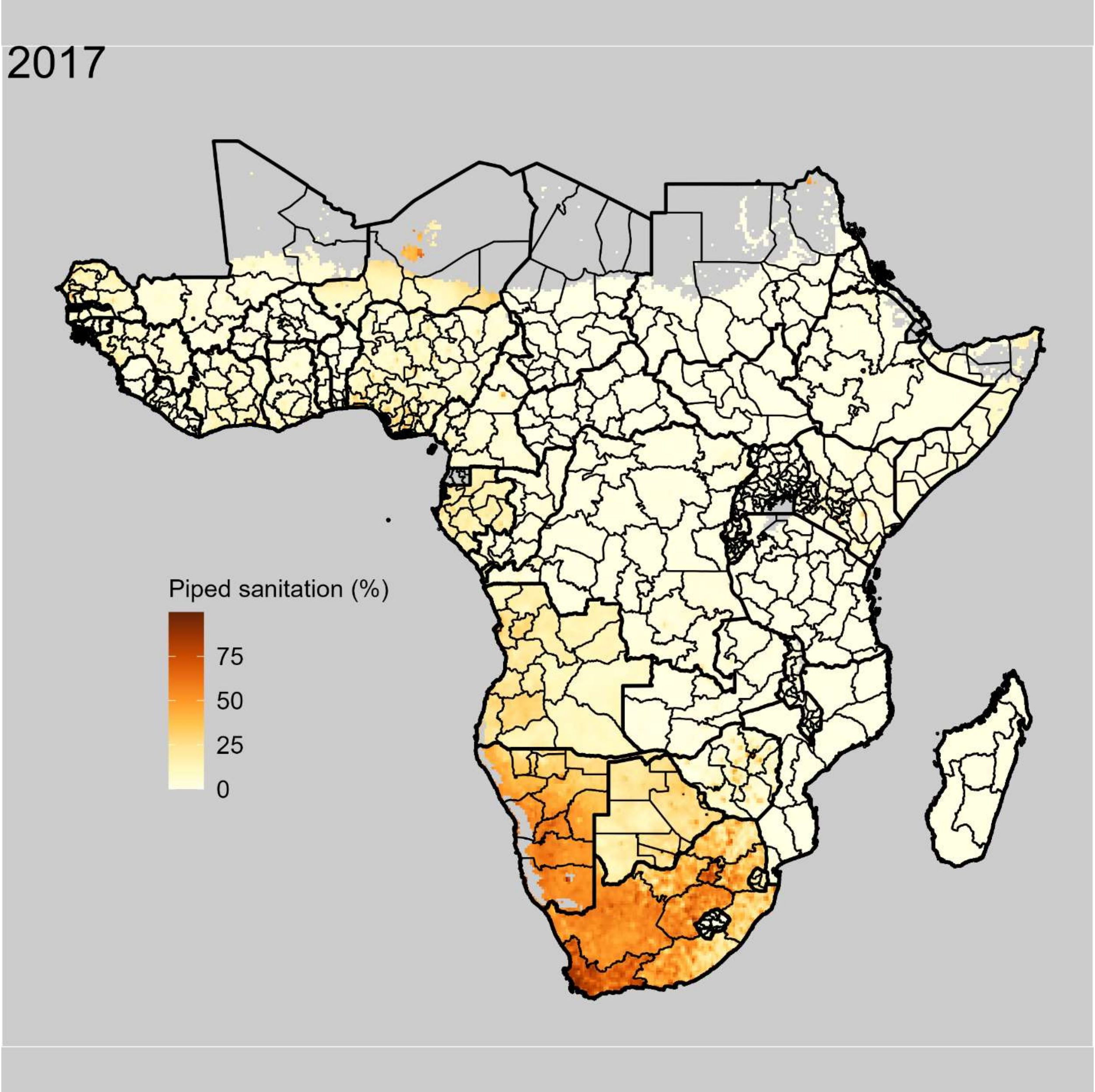

Figure S6. Travel time to the nearest healthcare facility. The values are based on a study that produce maps of travel time without access to motorized transport at 1 km by 1 km grids [5].

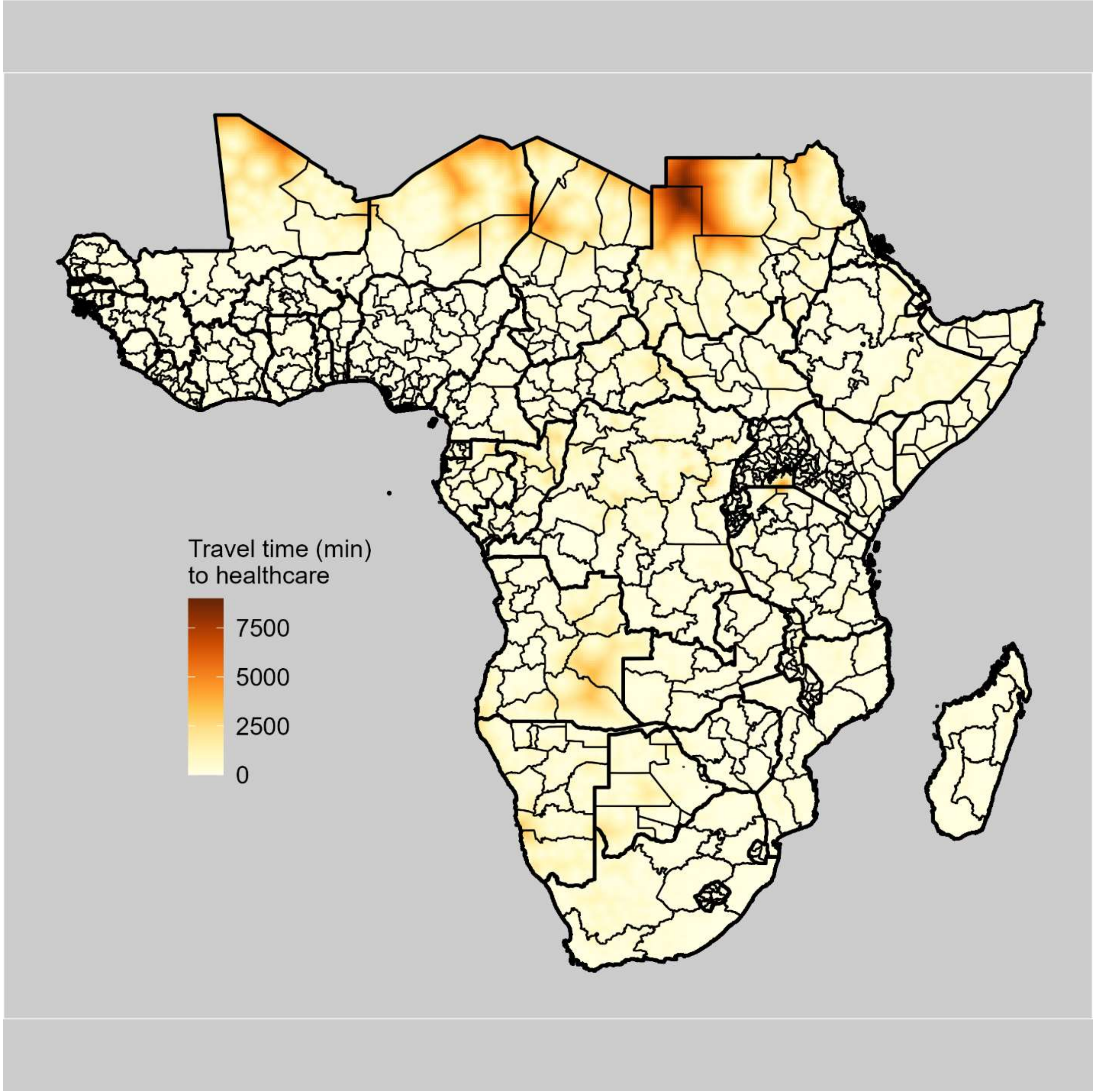

Table S1. Correlation among covariates. Pearson's *r* was computed using a data set of distance decay constant *k*=0.01. Highlighted cells indicate correlation coefficients among variables included in the final model.

	Pf incidence rate	Pf parasite rate	Improved water*	Open defecation	Piped sanitation*	Piped water	HIV*	Stunting	Surface water	Underweight*	Wasting
Pf incidence* rate	1	0.95	-0.35	-0.01	-0.46	-0.44	-0.15	0.3	0.04	0.08	-0.17
Pf parasite rate	0.95	1	-0.31	-0.05	-0.38	-0.36	-0.16	0.25	0.03	0.07	-0.13
Improved water*	-0.35	-0.31	1	-0.14	0.51	0.68	0.37	-0.45	-0.56	-0.39	-0.09
Open defecation	-0.01	-0.05	-0.14	1	-0.21	-0.09	-0.09	0.13	0.16	0.34	0.32
Piped sanitation*	-0.46	-0.38	0.51	-0.21	1	0.68	0.24	-0.29	-0.14	-0.28	-0.1
Piped water	-0.44	-0.36	0.68	-0.09	0.68	1	0.39	-0.35	-0.28	-0.35	-0.13
HIV*	-0.15	-0.16	0.37	-0.09	0.24	0.39	1	-0.17	-0.14	-0.57	-0.47
Stunting	0.3	0.25	-0.45	0.13	-0.29	-0.35	-0.17	1	0.34	0.58	0.08
Surface water	0.04	0.03	-0.56	0.16	-0.14	-0.28	-0.14	0.34	1	0.14	-0.11
Underweight*	0.08	0.07	-0.39	0.34	-0.28	-0.35	-0.57	0.58	0.14	1	0.76
Wasting	-0.17	-0.13	-0.09	0.32	-0.1	-0.13	-0.47	0.08	-0.11	0.76	1

* Covariates included in the final model

For peer review only

Figure S7. Receiver operating curve (ROC) from boosted regression tree model fits. The solid line represents the mean and dashed lines represent lower and upper bounds of the 95% confidence intervals. Area Under the ROC Curve (AUC) is 0.913 [95% CI: 0.911 to 0.915].

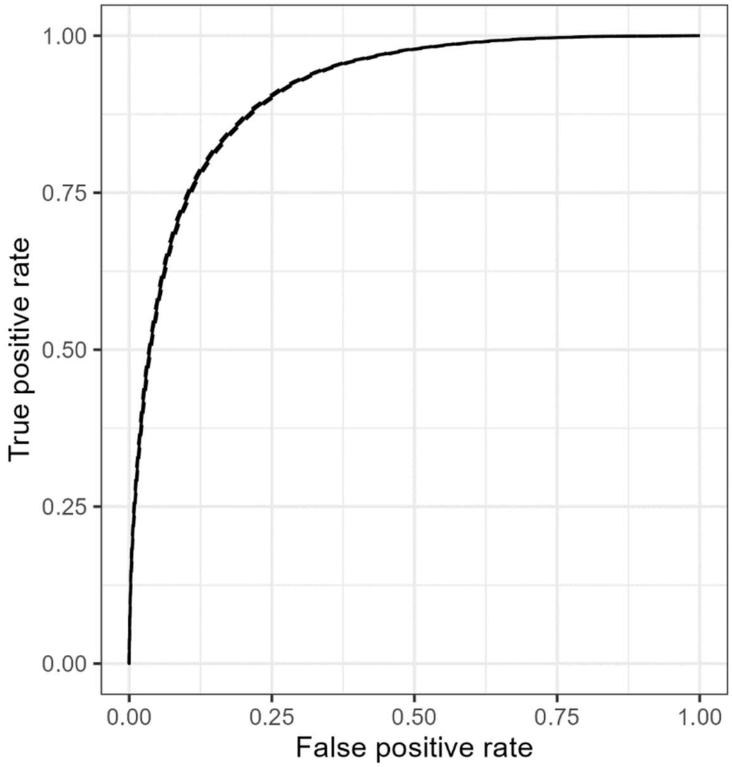

Table S2. Probability of iNTS occurrence for 2017 aggregated at the country level. Mean and the 95% confidence interval of the predicted probability from 400 simulation runs.

Country	Probability of occurrence
Angola	0.14 (95% CI: 0.07 to 0.25)
Burundi	0.32 (95% CI: 0.17 to 0.52)
Benin	0.25 (95% CI: 0.11 to 0.48)
Burkina Faso	0.29 (95% CI: 0.14 to 0.53)
Botswana	0.07 (95% CI: 0.03 to 0.18)
Central African Republic	0.47 (95% CI: 0.30 to 0.64)
Cote d'Ivoire	0.40 (95% CI: 0.24 to 0.61)
Cameroon	0.33 (95% CI: 0.19 to 0.50)
Congo, Democratic Republic of the	0.46 (95% CI: 0.26 to 0.68)
Congo, Republic of the	0.60 (95% CI: 0.40 to 0.78)

Djibouti	0.07 (95% CI: 0.03 to 0.13)
Eritrea	0.01 (95% CI: 0.01 to 0.03)
Ethiopia	0.06 (95% CI: 0.03 to 0.11)
Gabon	0.13 (95% CI: 0.03 to 0.41)
Ghana	0.66 (95% CI: 0.37 to 0.87)
Guinea	0.66 (95% CI: 0.41 to 0.86)
Gambia	0.42 (95% CI: 0.21 to 0.64)
Guinea-Bissau	0.59 (95% CI: 0.39 to 0.79)
Equatorial Guinea	0.02 (95% CI: 0.00 to 0.14)
Kenya	0.34 (95% CI: 0.19 to 0.51)
Liberia	0.48 (95% CI: 0.22 to 0.77)
Lesotho	0.25 (95% CI: 0.10 to 0.48)
Madagascar	0.03 (95% CI: 0.02 to 0.07)
Mali	0.38 (95% CI: 0.24 to 0.55)
Mozambique	0.58 (95% CI: 0.34 to 0.78)
Malawi	0.65 (95% CI: 0.49 to 0.81)
Namibia	0.10 (95% CI: 0.04 to 0.20)
Niger	0.02 (95% CI: 0.01 to 0.04)
Nigeria	0.21 (95% CI: 0.11 to 0.36)
Rwanda	0.38 (95% CI: 0.18 to 0.68)
Sudan	0.01 (95% CI: 0.00 to 0.01)
Senegal	0.30 (95% CI: 0.12 to 0.52)
Sierra Leone	0.23 (95% CI: 0.10 to 0.45)
Somalia	0.03 (95% CI: 0.01 to 0.07)
South Sudan	0.13 (95% CI: 0.07 to 0.25)
Swaziland	0.24 (95% CI: 0.08 to 0.74)
Chad	0.11 (95% CI: 0.06 to 0.19)
Togo	0.65 (95% CI: 0.42 to 0.82)
Tanzania, United Republic of	0.15 (95% CI: 0.06 to 0.33)
Uganda	0.23 (95% CI: 0.09 to 0.47)
South Africa	0.07 (95% CI: 0.03 to 0.18)
Zambia	0.49 (95% CI: 0.29 to 0.69)
Zimbabwe	0.40 (95% CI: 0.20 to 0.63)

For peer review only

Figure S8. Probability of iNTS occurrence from 2000 to 2020 every two years. Estimates represent the mean from 400 simulation runs. Areas for which data are not available are not colored. For animation, see https://github.com/kimfinale/iNTS_Mapping/tree/master/images.

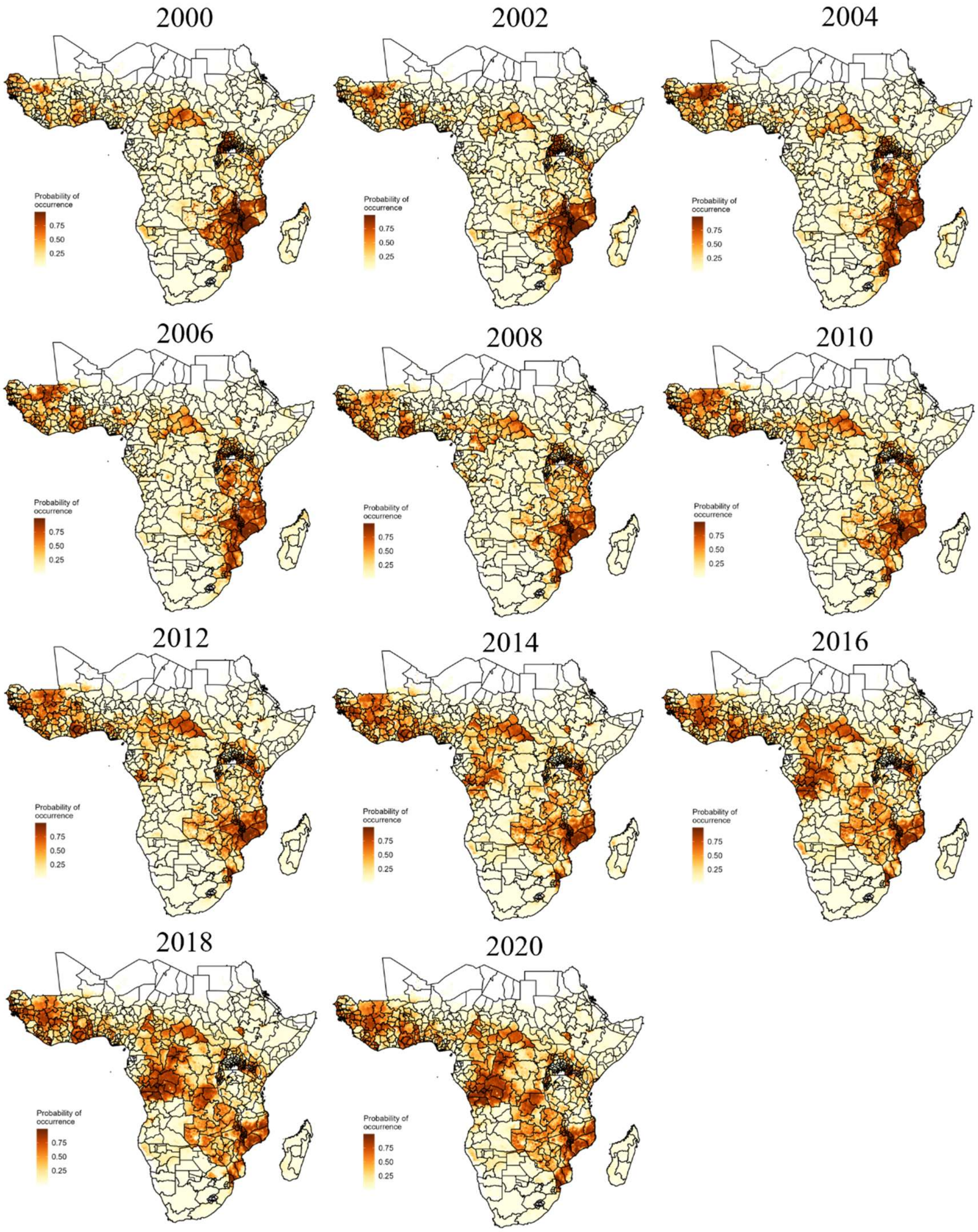

Table S3. Probability of iNTS occurrence aggregated at the regional level over 2000 - 2020. Values represent means from 400 simulation runs.

	Eastern Africa	Middle Africa	Southern Africa	Western Africa
2000	0.27	0.11	0.05	0.19
2001	0.28	0.11	0.06	0.19
2002	0.26	0.11	0.05	0.22
2003	0.27	0.11	0.05	0.24
2004	0.29	0.11	0.06	0.25
2005	0.28	0.12	0.05	0.25
2006	0.27	0.12	0.05	0.27
2007	0.27	0.14	0.05	0.27
2008	0.25	0.14	0.05	0.26
2009	0.25	0.16	0.04	0.28
2010	0.26	0.15	0.04	0.29
2011	0.25	0.16	0.04	0.30
2012	0.26	0.18	0.04	0.32
2013	0.26	0.20	0.04	0.32
2014	0.26	0.22	0.06	0.32
2015	0.25	0.25	0.04	0.33
2016	0.25	0.29	0.05	0.33
2017	0.24	0.34	0.08	0.33
2018	0.24	0.35	0.06	0.33
2019	0.24	0.35	0.05	0.33
2020	0.24	0.35	0.06	0.33

For peer review only

Table S4. Probability of iNTS occurrence aggregated at the country level over 2000 - 2020. Values represent means from 400 simulation runs.

Country	2000	2001	2002	2003	2004	2005	2006	2007	2008	2009	2010	2011	2012	2013	2014	2015	2016	2017	2018	2019	2020
Angola	0.05	0.05	0.05	0.05	0.05	0.05	0.05	0.05	0.05	0.05	0.05	0.05	0.06	0.08	0.09	0.11	0.13	0.14	0.14	0.14	0.14
Burundi	0.30	0.29	0.27	0.24	0.17	0.17	0.19	0.23	0.29	0.33	0.32	0.31	0.29	0.26	0.27	0.27	0.30	0.32	0.35	0.36	0.37
Benin	0.28	0.29	0.30	0.27	0.20	0.19	0.22	0.19	0.19	0.19	0.16	0.19	0.22	0.24	0.24	0.26	0.26	0.25	0.24	0.25	0.25
Burkina Faso	0.08	0.06	0.05	0.05	0.06	0.06	0.06	0.08	0.12	0.15	0.21	0.26	0.31	0.30	0.28	0.24	0.22	0.29	0.29	0.29	0.30
Botswana	0.02	0.02	0.01	0.01	0.01	0.01	0.01	0.01	0.02	0.03	0.04	0.02	0.02	0.02	0.04	0.02	0.04	0.07	0.04	0.03	0.04
CAR	0.45	0.42	0.41	0.42	0.42	0.42	0.44	0.45	0.46	0.50	0.48	0.46	0.55	0.53	0.52	0.50	0.51	0.47	0.46	0.46	0.46
Cote d'Ivoire	0.26	0.25	0.24	0.23	0.24	0.28	0.29	0.26	0.22	0.21	0.23	0.26	0.34	0.44	0.47	0.46	0.45	0.40	0.40	0.39	0.39
Cameroon	0.05	0.05	0.06	0.06	0.08	0.09	0.09	0.16	0.25	0.34	0.29	0.29	0.26	0.27	0.28	0.30	0.32	0.33	0.33	0.34	0.33
DR Congo,	0.09	0.09	0.09	0.10	0.10	0.10	0.10	0.10	0.10	0.11	0.11	0.13	0.15	0.17	0.20	0.25	0.34	0.46	0.48	0.48	0.48
Congo	0.05	0.05	0.05	0.07	0.07	0.08	0.09	0.10	0.12	0.15	0.19	0.23	0.28	0.38	0.52	0.63	0.65	0.60	0.59	0.59	0.59
Djibouti	0.02	0.02	0.03	0.04	0.03	0.05	0.05	0.04	0.03	0.04	0.03	0.03	0.03	0.03	0.04	0.05	0.06	0.07	0.08	0.11	0.12
Eritrea	0.04	0.05	0.04	0.04	0.02	0.02	0.02	0.02	0.01	0.01	0.01	0.01	0.01	0.01	0.01	0.01	0.01	0.01	0.01	0.02	0.02
Ethiopia	0.02	0.02	0.03	0.04	0.04	0.03	0.03	0.03	0.03	0.04	0.05	0.05	0.07	0.08	0.06	0.06	0.06	0.06	0.05	0.05	0.06
Gabon	0.03	0.04	0.06	0.04	0.07	0.08	0.12	0.21	0.17	0.14	0.12	0.11	0.13	0.11	0.11	0.12	0.12	0.13	0.14	0.14	0.13
Ghana	0.43	0.43	0.57	0.55	0.51	0.52	0.58	0.64	0.66	0.66	0.64	0.61	0.61	0.60	0.60	0.62	0.67	0.66	0.64	0.64	0.66
Guinea	0.27	0.33	0.44	0.48	0.42	0.38	0.40	0.45	0.50	0.55	0.56	0.56	0.61	0.64	0.66	0.66	0.66	0.66	0.67	0.68	0.68
Gambia	0.35	0.43	0.51	0.54	0.50	0.48	0.50	0.48	0.48	0.43	0.43	0.46	0.51	0.54	0.53	0.54	0.54	0.42	0.43	0.41	0.43
Guinea-Bissau	0.45	0.41	0.38	0.40	0.46	0.48	0.50	0.52	0.52	0.52	0.52	0.52	0.54	0.54	0.56	0.57	0.59	0.59	0.59	0.57	0.55
Equatorial Guinea	0.20	0.20	0.22	0.17	0.11	0.05	0.03	0.03	0.03	0.04	0.05	0.04	0.03	0.02	0.01	0.01	0.01	0.02	0.02	0.02	0.02
Kenya	0.19	0.20	0.20	0.20	0.19	0.20	0.20	0.22	0.27	0.27	0.30	0.31	0.36	0.34	0.32	0.32	0.33	0.34	0.32	0.30	0.30
Liberia	0.07	0.08	0.10	0.14	0.22	0.34	0.45	0.53	0.57	0.53	0.49	0.49	0.50	0.50	0.49	0.46	0.43	0.48	0.54	0.57	0.59
Lesotho	0.03	0.03	0.04	0.03	0.03	0.04	0.04	0.05	0.06	0.08	0.10	0.12	0.12	0.14	0.18	0.19	0.22	0.25	0.25	0.25	0.25
Madagascar	0.07	0.13	0.11	0.11	0.08	0.09	0.07	0.04	0.03	0.02	0.02	0.02	0.03	0.04	0.05	0.04	0.03	0.03	0.03	0.03	0.03
Mali	0.18	0.24	0.32	0.42	0.46	0.41	0.45	0.42	0.34	0.44	0.47	0.47	0.46	0.46	0.43	0.41	0.41	0.38	0.36	0.36	0.39
Mozambique	0.77	0.80	0.81	0.80	0.79	0.77	0.75	0.73	0.70	0.68	0.67	0.68	0.65	0.63	0.60	0.58	0.59	0.58	0.59	0.59	0.60
Malawi	0.88	0.88	0.87	0.84	0.82	0.80	0.76	0.70	0.65	0.62	0.60	0.64	0.64	0.63	0.64	0.63	0.64	0.65	0.64	0.64	0.66

For peer review only - http://bmjopen.bmj.com/site/about/guidelines.xhtml

Namibia	0.08	0.08	0.06	0.05	0.05	0.04	0.05	0.04	0.03	0.02	0.02	0.02	0.02	0.03	0.08	0.07	0.10	0.10	0.10	0.11	0.09
Niger	0.02	0.02	0.02	0.02	0.02	0.02	0.02	0.02	0.02	0.02	0.02	0.02	0.02	0.01	0.01	0.02	0.02	0.02	0.02	0.02	0.02
Nigeria	0.11	0.11	0.12	0.11	0.13	0.14	0.14	0.15	0.14	0.14	0.14	0.15	0.17	0.16	0.18	0.20	0.21	0.21	0.21	0.21	0.21
Rwanda	0.31	0.34	0.37	0.40	0.43	0.43	0.39	0.36	0.30	0.25	0.22	0.25	0.28	0.33	0.45	0.49	0.41	0.38	0.38	0.41	0.59
Sudan	0.00	0.00	0.00	0.00	0.00	0.00	0.00	0.00	0.00	0.00	0.00	0.00	0.00	0.01	0.01	0.01	0.01	0.01	0.01	0.01	0.01
Senegal	0.44	0.26	0.27	0.35	0.31	0.31	0.36	0.35	0.34	0.29	0.35	0.32	0.34	0.31	0.26	0.31	0.27	0.30	0.35	0.29	0.36
Sierra Leone	0.24	0.21	0.17	0.30	0.42	0.47	0.45	0.46	0.39	0.30	0.30	0.23	0.24	0.27	0.29	0.28	0.25	0.23	0.23	0.23	0.22
Somalia	0.12	0.10	0.09	0.09	0.09	0.08	0.07	0.05	0.04	0.03	0.03	0.02	0.02	0.02	0.02	0.02	0.02	0.03	0.03	0.04	0.03
South Sudan	0.07	0.07	0.08	0.07	0.07	0.07	0.08	0.08	0.09	0.11	0.12	0.12	0.12	0.12	0.13	0.13	0.13	0.13	0.13	0.14	0.14
Swaziland	0.53	0.59	0.63	0.66	0.70	0.66	0.64	0.61	0.57	0.54	0.49	0.45	0.42	0.41	0.36	0.31	0.26	0.24	0.25	0.23	0.23
Chad	0.06	0.06	0.05	0.04	0.04	0.04	0.08	0.10	0.09	0.08	0.08	0.08	0.09	0.10	0.11	0.10	0.10	0.11	0.11	0.11	0.11
Togo	0.14	0.17	0.17	0.18	0.19	0.20	0.22	0.23	0.26	0.30	0.37	0.39	0.44	0.45	0.53	0.56	0.60	0.65	0.65	0.65	0.65
Tanzania	0.22	0.21	0.19	0.33	0.51	0.53	0.48	0.40	0.36	0.32	0.30	0.28	0.26	0.28	0.28	0.25	0.20	0.15	0.15	0.15	0.15
Uganda	0.65	0.65	0.63	0.60	0.59	0.56	0.51	0.47	0.46	0.45	0.43	0.41	0.36	0.31	0.27	0.24	0.23	0.23	0.22	0.21	0.23
South Africa	0.03	0.06	0.06	0.07	0.07	0.06	0.06	0.06	0.05	0.05	0.05	0.05	0.05	0.05	0.05	0.03	0.03	0.07	0.05	0.03	0.03
Zambia	0.32	0.33	0.29	0.27	0.25	0.25	0.25	0.26	0.28	0.30	0.32	0.35	0.39	0.43	0.45	0.47	0.49	0.49	0.49	0.48	0.48
Zimbabwe	0.61	0.52	0.44	0.37	0.34	0.29	0.30	0.48	0.36	0.39	0.46	0.33	0.37	0.37	0.38	0.38	0.37	0.40	0.41	0.40	0.37

For peer review only

Figure S9. Probability of iNTS occurrence for 2000, 2010, and 2017 for *k*=0.01, *k*=0.02, *k*=0.005. Average radius for the catchment area for the secondary and tertiary hospital is 100 km (baseline), 50 km, and 200 km, respectively for *k*=0.01, *k*=0.02, and *k*=0.005. Areas for which data are not available are not colored.

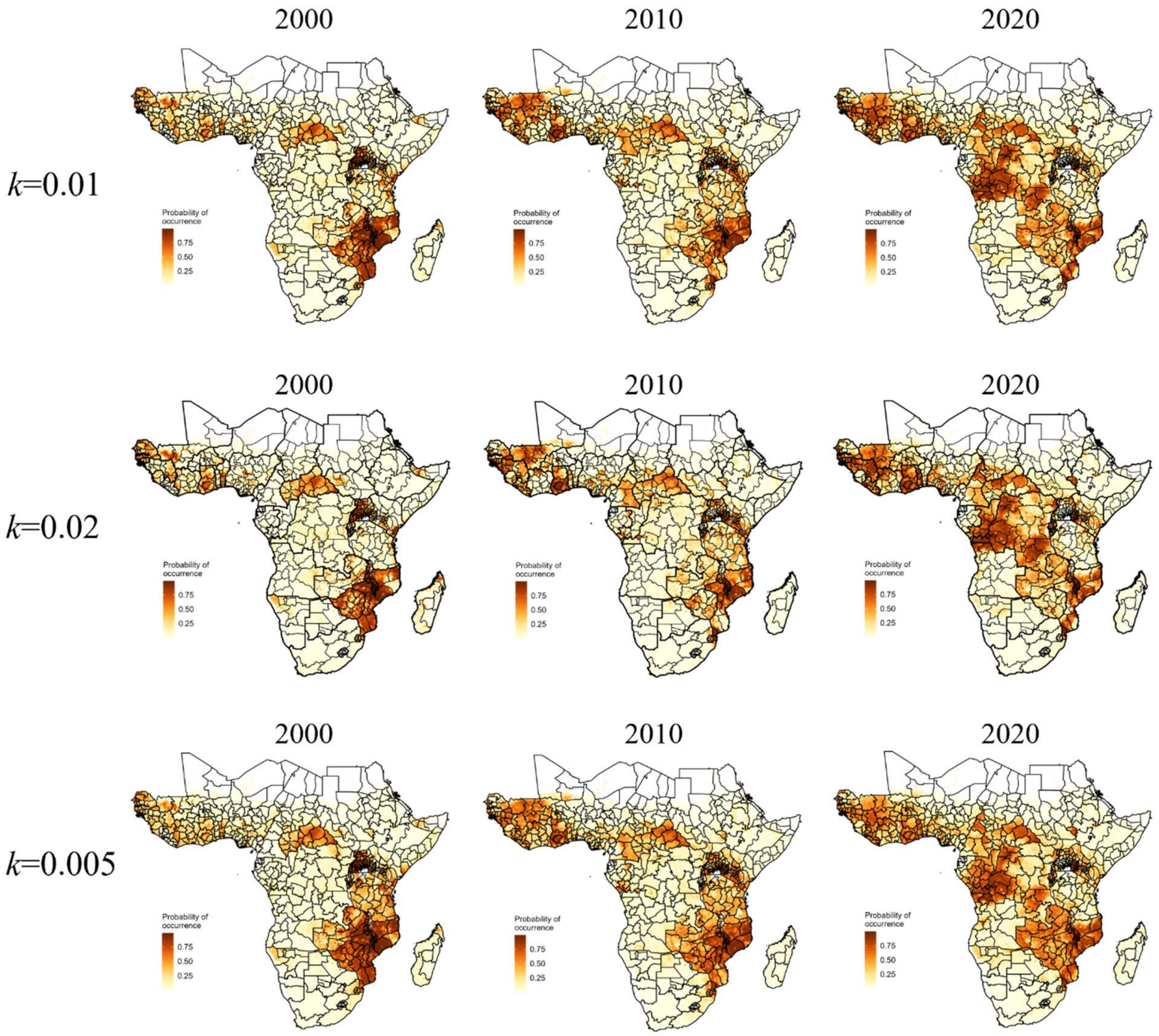

PRISMA 2020 for Abstracts Checklist

Section and Topic	Item #	Checklist item	Reported (Yes/No)
**TITLE**			
Title	1	Identify the report as a systematic review.	Yes
**BACKGROUND**			
Objectives	2	Provide an explicit statement of the main objective(s) or question(s) the review addresses.	Yes
**METHODS**			
Eligibility criteria	3	Specify the inclusion and exclusion criteria for the review.	Yes
Information sources	4	Specify the information sources (e.g. databases, registers) used to identify studies and the date when each was last searched.	Yes
Risk of bias	5	Specify the methods used to assess risk of bias in the included studies.	No
Synthesis of results	6	Specify the methods used to present and synthesise results.	Yes
**RESULTS**			
Included studies	7	Give the total number of included studies and participants and summarise relevant characteristics of studies.	Yes
Synthesis of results	8	Present results for main outcomes, preferably indicating the number of included studies and participants for each. If meta-analysis was done, report the summary estimate and confidence/credible interval. If comparing groups, indicate the direction of the effect (i.e. which group is favoured).	Yes
**DISCUSSION**			
Limitations of evidence	9	Provide a brief summary of the limitations of the evidence included in the review (e.g. study risk of bias, inconsistency and imprecision).	Yes
Interpretation	10	Provide a general interpretation of the results and important implications.	Yes
**OTHER**			
Funding	11	Specify the primary source of funding for the review.	No
Registration	12	Provide the register name and registration number.	No

From: Page MJ, McKenzie JE, Bossuyt PM, Boutron I, Hoffmann TC, Mulrow CD, et al. The PRISMA 2020 statement: an updated guideline for reporting systematic reviews. BMJ 2021;372:n71. doi: 10.1136/bmj.n71

For more information, visit: http://www.prisma-statement.org/

For peer review only

PRISMA 2020 Checklist

Section and Topic	Item #	Checklist item	Location where item is reported
**TITLE**			
Title	1	Identify the report as a systematic review.	Line 2
**ABSTRACT**			
Abstract	2	See the PRISMA 2020 for Abstracts checklist.	
**INTRODUCTION**			
Rationale	3	Describe the rationale for the review in the context of existing knowledge.	Lines 76-79
Objectives	4	Provide an explicit statement of the objective(s) or question(s) the review addresses.	Lines 79-81
**METHODS**			
Eligibility criteria	5	Specify the inclusion and exclusion criteria for the review and how studies were grouped for the syntheses.	Lines 95-101
Information sources	6	Specify all databases, registers, websites, organisations, reference lists and other sources searched or consulted to identify studies. Specify the date when each source was last searched or consulted.	Lines 85-87
Search strategy	7	Present the full search strategies for all databases, registers and websites, including any filters and limits used.	Lines 87-89
Selection process	8	Specify the methods used to decide whether a study met the inclusion criteria of the review, including how many reviewers screened each record and each report retrieved, whether they worked independently, and if applicable, details of automation tools used in the process.	Lines 93-95
Data collection process	9	Specify the methods used to collect data from reports, including how many reviewers collected data from each report, whether they worked independently, any processes for obtaining or confirming data from study investigators, and if applicable, details of automation tools used in the process.	Lines 93-95
Data items	10a	List and define all outcomes for which data were sought. Specify whether all results that were compatible with each outcome domain in each study were sought (e.g. for all measures, time points, analyses), and if not, the methods used to decide which results to collect.	Lines 100-103
	10b	List and define all other variables for which data were sought (e.g. participant and intervention characteristics, funding sources). Describe any assumptions made about any missing or unclear information.	Lines 103-105
Study risk of bias assessment	11	Specify the methods used to assess risk of bias in the included studies, including details of the tool(s) used, how many reviewers assessed each study and whether they worked independently, and if applicable, details of automation tools used in the process.	NA
Effect measures	12	Specify for each outcome the effect measure(s) (e.g. risk ratio, mean difference) used in the synthesis or presentation of results.	Lines 166-188
Synthesis methods	13a	Describe the processes used to decide which studies were eligible for each synthesis (e.g. tabulating the study intervention characteristics and comparing against the planned groups for each synthesis (item #5)).	Lines 95-100
	13b	Describe any methods required to prepare the data for presentation or synthesis, such as handling of missing summary statistics, or data conversions.	Lines 106-125
	13c	Describe any methods used to tabulate or visually display results of individual studies and syntheses.	Lines 213-225
	13d	Describe any methods used to synthesize results and provide a rationale for the choice(s). If meta-analysis was performed, describe the model(s), method(s) to identify the presence and extent of statistical heterogeneity, and software package(s) used.	Lines 213-225
	13e	Describe any methods used to explore possible causes of heterogeneity among study results (e.g. subgroup analysis, meta-regression).	Lines 189-212
	13f	Describe any sensitivity analyses conducted to assess robustness of the synthesized results.	Lines 189-212

For peer review only - http://bmjopen.bmj.com/site/about/guidelines.xhtml
For peer review only

PRISMA 2020 Checklist

Section and Topic	Item #	Checklist item	Location where item is reported
Reporting bias assessment	14	Describe any methods used to assess risk of bias due to missing results in a synthesis (arising from reporting biases).	NA
Certainty assessment	15	Describe any methods used to assess certainty (or confidence) in the body of evidence for an outcome.	Lines 190-212
**RESULTS**			
Study selection	16a	Describe the results of the search and selection process, from the number of records identified in the search to the number of studies included in the review, ideally using a flow diagram.	Lines 229-234
	16b	Cite studies that might appear to meet the inclusion criteria, but which were excluded, and explain why they were excluded.	NA
Study characteristics	17	Cite each included study and present its characteristics.	NA
Risk of bias in studies	18	Present assessments of risk of bias for each included study.	NA
Results of individual studies	19	For all outcomes, present, for each study: (a) summary statistics for each group (where appropriate) and (b) an effect estimate and its precision (e.g. confidence/credible interval), ideally using structured tables or plots.	NA
Results of syntheses	20a	For each synthesis, briefly summarise the characteristics and risk of bias among contributing studies.	NA
	20b	Present results of all statistical syntheses conducted. If meta-analysis was done, present for each the summary estimate and its precision (e.g. confidence/credible interval) and measures of statistical heterogeneity. If comparing groups, describe the direction of the effect.	NA
	20c	Present results of all investigations of possible causes of heterogeneity among study results.	NA
	20d	Present results of all sensitivity analyses conducted to assess the robustness of the synthesized results.	NA
Reporting biases	21	Present assessments of risk of bias due to missing results (arising from reporting biases) for each synthesis assessed.	NA
Certainty of evidence	22	Present assessments of certainty (or confidence) in the body of evidence for each outcome assessed.	NA
**DISCUSSION**			
Discussion	23a	Provide a general interpretation of the results in the context of other evidence.	Lines 282-288
	23b	Discuss any limitations of the evidence included in the review.	Lines 309-335
	23c	Discuss any limitations of the review processes used.	NA
	23d	Discuss implications of the results for practice, policy, and future research.	Lines 338-344
**OTHER INFORMATION**			
Registration and protocol	24a	Provide registration information for the review, including register name and registration number, or state that the review was not registered.	NA
	24b	Indicate where the review protocol can be accessed, or state that a protocol was not prepared.	NA
	24c	Describe and explain any amendments to information provided at registration or in the protocol.	NA
Support	25	Describe sources of financial or non-financial support for the review, and the role of the funders or sponsors in the review.	Lines 356-347

For peer review only - http://bmjopen.bmj.com/site/about/guidelines.xhtml

PRISMA 2020 Checklist

Section and Topic	Item #	Checklist item	Location where item is reported
Competing interests	26	Declare any competing interests of review authors.	NA
Availability of data, code and other materials	27	Report which of the following are publicly available and where they can be found: template data collection forms; data extracted from included studies; data used for all analyses; analytic code; any other materials used in the review.	Line 212

From: Page MJ, McKenzie JE, Bossuyt PM, Boutron I, Hoffmann TC, Mulrow CD, et al. The PRISMA 2020 statement: an updated guideline for reporting systematic reviews. BMJ 2021;372:n71. doi: 10.1136/bmj.n71

For more information, visit: http://www.prisma-statement.org/

For peer review only
